# Inhibition of profibrotic microRNA-21 affects platelets and their releasate

**DOI:** 10.1172/jci.insight.123335

**Published:** 2018-11-02

**Authors:** Temo Barwari, Seda Eminaga, Ursula Mayr, Ruifang Lu, Paul C. Armstrong, Melissa V. Chan, Mahnaz Sahraei, Marta Fernández-Fuertes, Thomas Moreau, Javier Barallobre-Barreiro, Marc Lynch, Xiaoke Yin, Christian Schulte, Ferheen Baig, Raimund Pechlaner, Sarah R. Langley, Anna Zampetaki, Peter Santer, Martin Weger, Roberto Plasenzotti, Markus Schosserer, Johannes Grillari, Stefan Kiechl, Johann Willeit, Ajay M. Shah, Cedric Ghevaert, Timothy D. Warner, Carlos Fernández-Hernando, Yajaira Suárez, Manuel Mayr

**Affiliations:** 1 King’s British Heart Foundation Centre, King’s College London, London, United Kingdom.; 2 Blizard Institute, Barts and The London School of Medicine and Dentistry, Queen Mary University of London, London, United Kingdom.; 3 Department of Comparative Medicine and Vascular Biology and Therapeutics Program, Yale University School of Medicine, New Haven, Connecticut, USA.; 4 Department of Haematology, University of Cambridge, National Health Blood Service Centre, Cambridge, United Kingdom.; 5 Department of Neurology, Medical University Innsbruck, Innsbruck, Austria.; 6 Duke-NUS Medical School, Singapore.; 7 National Heart Centre Singapore, Singapore.; 8 Department of Laboratory Medicine and; 9 Department of Internal Medicine, Bruneck Hospital, Bruneck, Italy.; 10 Medical University of Vienna, Institute of Biomedical Research, Vienna, Austria.; 11 Christian Doppler Laboratory on Biotechnology of Skin Aging, Department of Biotechnology, BOKU — University of Natural Resources and Life Sciences, Vienna, Austria.

**Keywords:** Cardiology, Cell Biology, Fibrosis, Noncoding RNAs, Platelets

## Abstract

Fibrosis is a major contributor to organ disease for which no specific therapy is available. MicroRNA-21 (miR-21) has been implicated in the fibrogenetic response, and inhibitors of miR-21 are currently undergoing clinical trials. Here, we explore how miR-21 inhibition may attenuate fibrosis using a proteomics approach. Transfection of miR-21 mimic or inhibitor in murine cardiac fibroblasts revealed limited effects on extracellular matrix (ECM) protein secretion. Similarly, miR-21–null mouse hearts showed an unaltered ECM composition. Thus, we searched for additional explanations as to how miR-21 might regulate fibrosis. In plasma samples from the community-based Bruneck Study, we found a marked correlation of miR-21 levels with several platelet-derived profibrotic factors, including TGF-β1. Pharmacological miR-21 inhibition with an antagomiR reduced the platelet release of TGF-β1 in mice. Mechanistically, Wiskott-Aldrich syndrome protein, a negative regulator of platelet TGF-β1 secretion, was identified as a direct target of miR-21. miR-21–null mice had lower platelet and leukocyte counts compared with littermate controls but higher megakaryocyte numbers in the bone marrow. Thus, to our knowledge this study reports a previously unrecognized effect of miR-21 inhibition on platelets. The effect of antagomiR-21 treatment on platelet TGF-β1 release, in particular, may contribute to the antifibrotic effects of miR-21 inhibitors.

## Abstract 2

MicroRNA-21 inhibition may convey its therapeutic benefits in fibrosis through its action in bone marrow cells rather than targeting fibroblasts directly.

## Graphical abstract

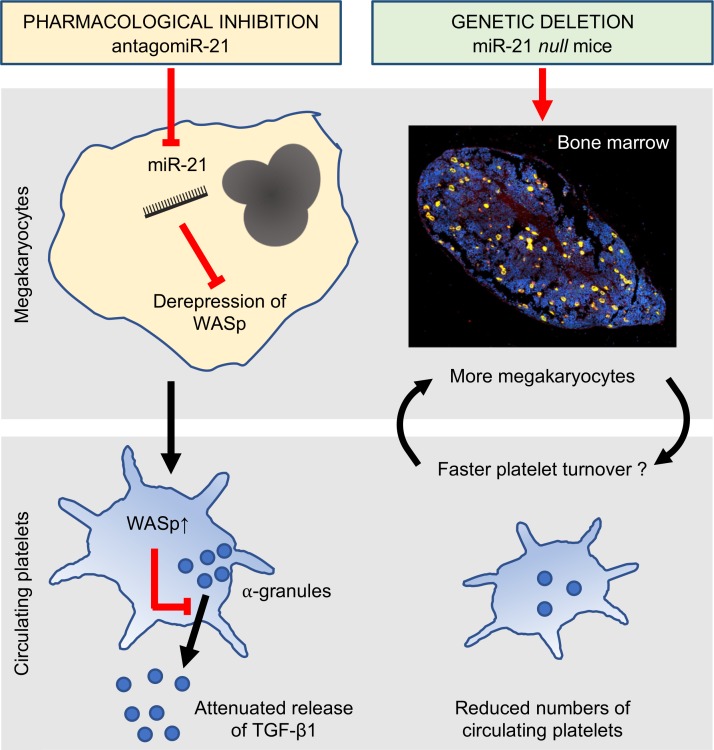

## Introduction

MicroRNAs (miRNAs) have been linked to cardiac remodeling, with miR-21 being implicated in cardiac fibrosis (1). Using different models of tissue injury, several, but not all (2), studies reported a reduction of cardiac fibrosis with systemic miR-21 inhibition (3–5). miR-21 is ubiquitously expressed in mammalian cell types. Besides myocardial fibrosis (3–6), it has been linked to fibrotic kidney (7, 8) and lung disease (9), in-stent restenosis and vein graft failure (10–12), and aortic aneurysmal disease (13). As a mechanistic explanation, studies of miR-21 have suggested an effect on cardiac fibroblast (CF) survival and proliferation that may contribute to fibrosis (2–6). miR-21 may also enhance fibrosis through modulating fibroblast senescence (14). Surprisingly, miR-21–null mice did not show a difference in cardiac function at baseline or with cardiac injury (2). Using a proteomics approach, we have previously reported the first detailed characterization of the CF secretome, identifying numerous targets of miR-29b among extracellular matrix (ECM) proteins (15). We pursued a similar approach to investigate the role of miR-21.

The “master switch” in fibrosis is widely regarded to be TGF-β1. In the heart, TGF-β1 expression is induced by an array of factors, including angiotensin II and mechanical stretch (16). In addition to cardiomyocytes and CFs, high TGF-β1 levels are also found in blood platelets, where its release is controlled by Wiskott-Aldrich syndrome protein (WASp) (17). The platelet releasate is supposed to be a key driver of the inflammatory response in cardiac remodeling (18). It has been suggested that extravasated platelets are a major source of TGF-β1 in the myocardium upon cardiac injury (16). Using megakaryocyte-specific TGF-β1–knockout mice, platelet TGF-β1 has indeed been implicated in the development of cardiac hypertrophy and fibrosis in several models of cardiac remodeling (19–21). We have previously established that many of the abundant circulating miRNAs are of platelet origin (22). Moreover, there is accumulating evidence that abundant platelet miRNAs, such as miR-223 and miR-126, also affect platelet function (23–25).

In this study, we demonstrate that genetic ablation of miR-21 reduces the number of circulating platelets, while pharmacological inhibition attenuates platelet release of TGF-β1 through miR-21–mediated targeting of WASp. In contrast, changes in miR-21 levels alter CF proliferation but have no major effect on the secretion of ECM proteins. Furthermore, we show that plasma concentrations of TGF-β1 are dependent on platelets and correlate strongly with miR-21 levels. Thus, in addition to directly acting on fibroblasts, miR-21 may contribute to fibrosis indirectly through its effects on platelets.

## Results

### Mimics and inhibitors of miR-21 affect murine CF proliferation.

**Figure 1:**
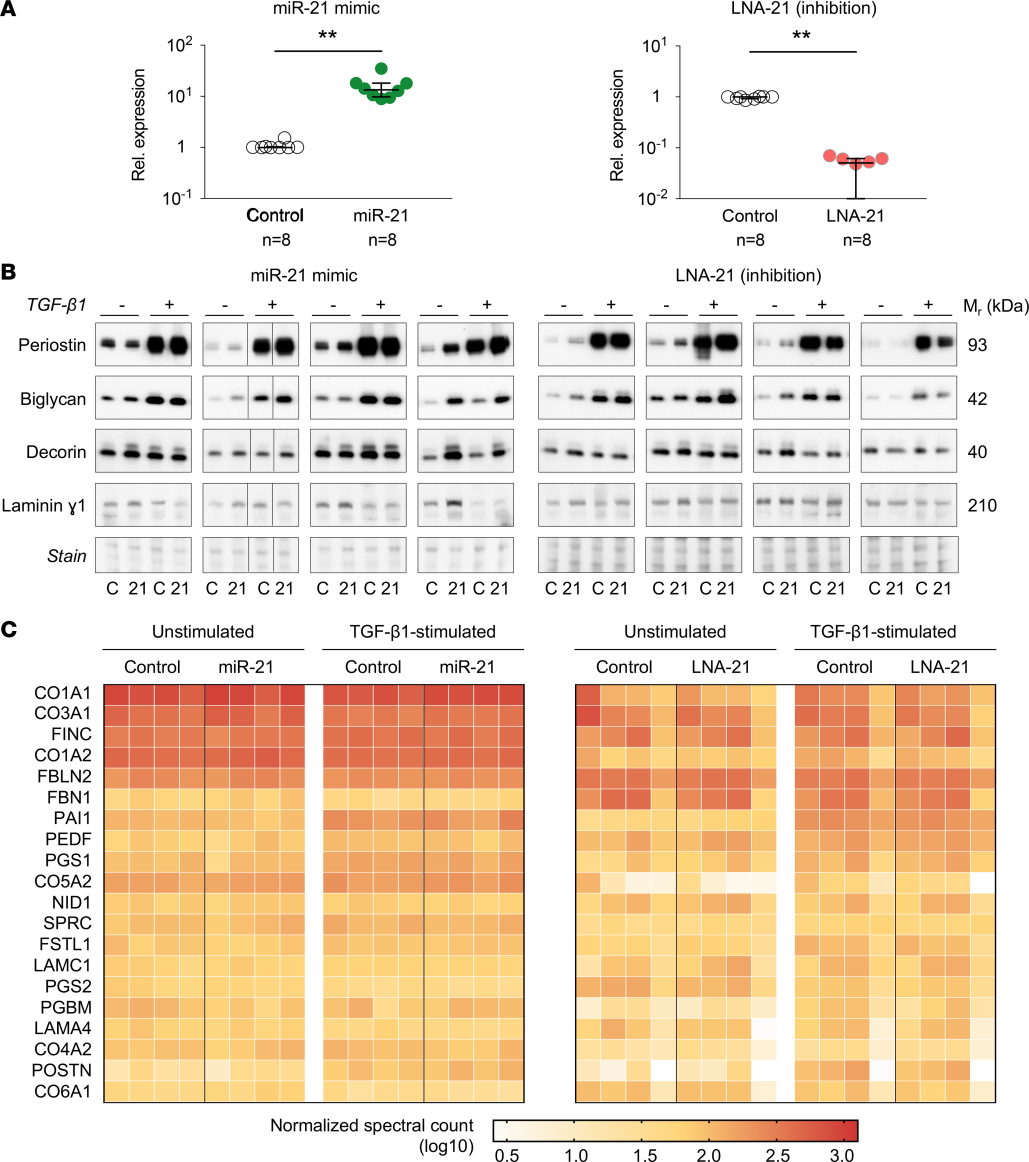
Transfections of cardiac fibroblasts with miR-21 mimic and inhibitor. (**A**) Cardiac fibroblasts (CFs) were isolated from wild-type mice and transfected with miR-21 mimic or LNA-21 (inhibitor), followed by stimulation with TGF-β1 or control treatment. Overexpression and inhibition were confirmed by qPCR (*n* = 8 for each transfection condition; Wilcoxon matched-pairs signed-rank test; lines and error bars represent median [IQR]; note that in 3 samples miR-21 was undetectable after transfection with LNA-21). (**B**) Immunoblotting for several extracellular matrix (ECM) proteins showed effects of TGF-β1 treatment but not of miR-21 mimic or inhibitor transfection (*n* = 4 for each condition). Ponceau S staining was used as loading control. C, control mimic/LNA; 21, miR-21 mimic/LNA-21; M_r_, relative mass. TGF-β1 +/– indicates treatment 48 hours prior to conditioned media collection. (**C**) Proteomic analysis of the CF secretome after transfections with miR-21 mimic or inhibitor identified no significant changes in the 20 most abundant ECM proteins. Four biological replicates were analyzed for each transfection type in the presence or absence of TGF-β1 treatment. No statistically significant difference was seen between miR-21 mimic or inhibitor and its respective control for any of the shown proteins, using a FDR < 0.05, calculated with the Empirical Bayes method.

Previous studies have reported a role of miR-21 in cardiac ECM remodeling, predominantly ascribing the attenuation of fibrosis upon miR-21 inhibition to the nonmyocyte or CF niche of the cardiac cell population (3, 4, 6, 26). To study the direct effects of miR-21 on this cell type using proteomics, CFs were isolated from hearts of 8- to 10-week-old male C57BL/6 mice as previously described (15). Cultured cells were transfected (Supplemental Figure 1) with a miR-21 mimic or inhibitor (locked nucleic acid 21 [LNA-21]) and respective controls (*n* = 4 per group). qPCR analysis of miR-21 levels confirmed a significant and specific effect of the transfections (Figure 1A and Supplemental Figure 2). To assess CF proliferation, cells were plated and monitored using an electrical impedance-based assay (xCELLigence). Real-time recording revealed an increase in proliferation within 24 hours after miR-21 mimic transfection, which was in line with previous findings (3). A concomitant reduction in proliferation was seen after miR-21 inhibitor transfection (Supplemental Figure 3).

### Mimics and inhibitors of miR-21 have a limited effect on ECM protein secretion.

To study the effects of miR-21 on the secretion of ECM proteins, isolated CFs were transfected, followed by stimulation with recombinant TGF-β1 or a vehicle control. After 48 hours of culturing in serum-free conditions, conditioned media were collected and processed for secretome analysis (Supplemental Figure 4). As expected, TGF-β1 markedly increased secretion of periostin (fold change [FC] = 4.5 and 10.3, *P* = 0.008 and 0.008 for miR-21 mimic and LNA-21–transfected cells, respectively) and biglycan (FC = 3.5 and 7.0, *P* = 0.016 and 0.008, respectively). No significant differences were observed for decorin and laminin γ1 (Figure 1B and Supplemental Figure 5).

Next, the secretome was analyzed using proteomics. Normalized spectral counts of ECM proteins identified by liquid chromatography tandem mass spectrometry (LC-MS/MS) are provided in Supplemental Table 3. Consistent with the immunoblotting results, periostin levels were markedly increased by TGF-β1 stimulation (Supplemental Figure 6A). Importantly, secretome levels for the 20 proteins with the highest number of identified spectra, which includes periostin, did not significantly differ after miR-21 mimic or inhibitor transfection (Figure 1C). Overall, a marginal effect of miR-21 on ECM secretion was observed (Supplemental Figure 7). After miR-21 mimic transfection, only insulin-like growth factor–binding protein 4 (IBP4) and granulin (GRN) showed a significant upregulation in unstimulated CFs, whereas higher levels of GRN, cathepsin L (CATL1), and the α-1 chain of collagen 11 (COBA1) were seen in TGF-β1–stimulated cells. Upon miR-21 inhibition, GRN showed a significant increase only in TGF-β1–stimulated cells, whereas galectin-3 binding protein (LG3BP) and VCAM-1 were increased in both unstimulated and TGF-β1–stimulated CFs (Supplemental Figure 8).

To complement the proteomic findings, changes in gene expression were determined. In response to TGF-β1, expression of commonly used markers of the myofibroblast-like phenotype (Supplemental Figure 6B), such as α smooth muscle actin (*Acta2*; FC = 1.6, *P* < 0.0001), periostin (*Postn*; FC = 3.2, *P* = 0.0001), and TGF-β1 itself (*Tgfb1*; FC = 1.3, *P* < 0.0001), was increased. Evaluation of transcripts corresponding to the 20 proteins with the highest number of identified spectra (Supplemental Figure 9) and those significantly changing in the secretome (Supplemental Figure 10) showed a trend toward higher expression of periostin and the transcript encoding LG3BP (*Lgals3bp*) after miR-21 mimic and inhibitor transfection, respectively. However, none of the changes in expression upon miR-21 overexpression or inhibition reached statistical significance. In summary, miR-21 had no major effect on the expression and secretion of ECM proteins by CFs in vitro.

### The cardiac ECM is unaffected in miR-21–null mice.

**Figure 2:**
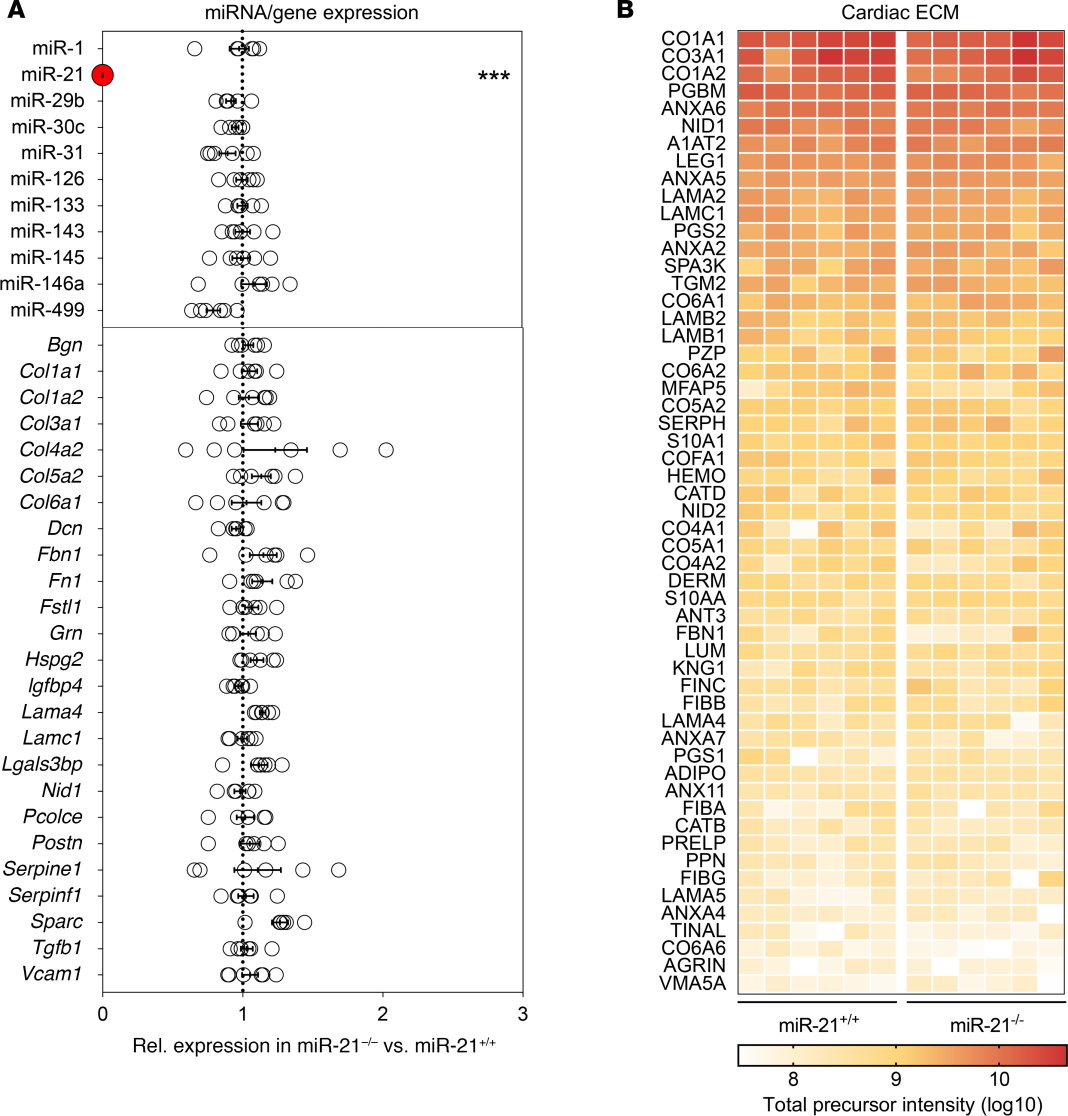
The cardiac ECM of miR-21–null mice. (**A**) Hearts from miR-21–null mice and littermate wild-type controls were used for RNA isolation. Expression levels of miRNAs (top) and selected ECM genes based on the CF transfection experiments (bottom) were determined by qPCR. Expression levels of *U6* and *Actb* were used as reference transcripts, respectively. (**B**) The same hearts were used for proteomics by combining our sequential ECM extraction procedure with analysis by mass spectrometry. Using normalized precursor intensities, no significant differences were observed between hearts from miR-21–null mice and littermate controls. An FDR-based approach was used for all 3 analyses using a *q* < 0.05 for statistical significance. For all 3 analyses, *n* = 6 miR-21-null mice vs. *n* = 6 control mice.

In contrast to the profound effects of pharmacological miR-21 inhibition on the fibrotic phenotype, previous analysis of miR-21–null mice reported a normal cardiac morphology and contractility (2). Since no comprehensive analysis of their cardiac ECM has been performed to date, we evaluated the ECM of ventricular cardiac tissue from miR-21–null mice and littermate controls (*n* = 6 per group). qPCR analysis confirmed undetectable levels of miR-21, whereas no differences were found for other abundant cardiac miRNAs (Figure 2A, top). Cardiac expression levels of genes encoding various ECM constituents were unaltered in miR-21–null mice (Figure 2A, bottom).

Next, a comprehensive proteomics analysis of the cardiac ECM was performed using our established 3-step sequential enrichment protocol (27, 28). FDR-corrected comparisons between both genotypes revealed no difference in abundance for any of the identified proteins (Figure 2B and Supplemental Table 4) (29). Thus, consistent with our in vitro results, a combined transcriptomic and proteomic analysis of the cardiac ECM revealed no major change upon genetic miR-21 deletion.

### miR-21 correlates with levels of platelet-derived profibrotic factors in the circulation.

**Figure 3:**
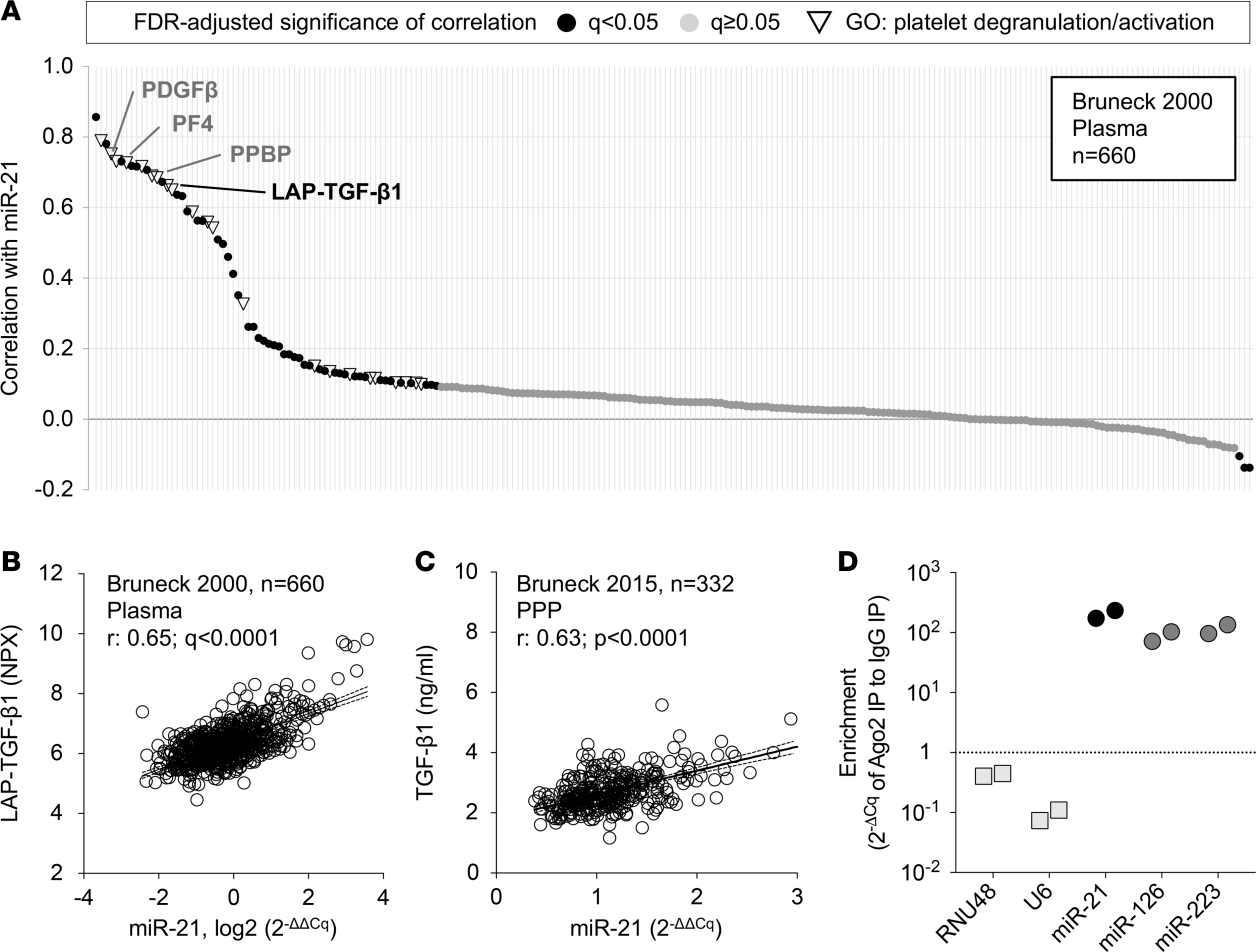
Plasma levels of miR-21 and TGF-β1. (**A**) Plasma miR-21 levels were measured using qPCR in samples from the year 2000 follow-up of the community-based Bruneck Study (*n* = 660). Pearson coefficients for the correlation of miR-21 with circulating proteins associated with cardiovascular disease and inflammation were calculated based on measurements by a combination of proximity extension assays, mass spectrometry, and ELISA. Each point indicates an individual protein, with its corresponding correlation coefficient on the *y* axis. PF4, platelet factor 4; PPBP, proplatelet basic protein; LAP-TGF-β1, latency-associated peptide of TGF-β1. Black points indicate FDR-adjusted significance < 0.05, with inverted triangles indicating significantly correlating proteins that were annotated with the “platelet degranulation” and “platelet activation” gene ontology (GO) terms. (**B**) The correlation of miR-21 and LAP-TGF-β1 in plasma of the Bruneck study, year 2000 follow-up. Solid and dashed lines indicate linear regression and 95% confidence interval, respectively. NPX, normalized protein expression. *q*, FDR-corrected *P* value for Pearson correlation. (**C**) The correlation of miR-21 and mature TGF-β1, as measured by ELISA, using platelet-poor plasma (PPP) from the 2015 evaluation of the Bruneck Study (*n* = 332). *r*, Pearson correlation coefficient; *P* value for Pearson correlation. (**D**) Argonaute 2 (Ago2) immunoprecipitation was performed in lysates from a human megakaryoblastic leukemia cell line (MEG-01) to isolate coprecipitated RNA. Analysis by qPCR showed enrichment for miR-21, along with miR-126 and -223 (25), suggesting a functional relevance for these miRNAs in megakaryocytes. *U6* and *RNU48* were used as control RNAs. *n* = 2 for each measurement.

Given the limited effects on ECM secretion by CFs, we explored additional explanations for the observed profibrotic role of miR-21. Previous studies have shown that miR-21 is abundant in hematopoietic cells and the circulation (23, 25, 30). We therefore measured 228 proteins in plasma from the community-based Bruneck Study (year 2000 evaluation, *n* = 660), using proximity extension assays, LC-MS/MS, and ELISA. These protein measurements were then correlated to circulating miR-21 levels, as determined by qPCR (Figure 3A). A significant correlation was seen between miR-21 and the latency-associated peptide of TGF-β1 (LAP-TGF-β1; r = 0.65, *P* < 0.0001; Figure 3B) as well as several other profibrotic factors, such as PDGF subunit β (PDGFβ; r = 0.73, *P* < 0.0001), suggesting a common origin. Furthermore, miR-21 levels were significantly correlated with those of markers of platelet activity, such as proplatelet basic protein (PPBP) and platelet factor 4 (PF4), as also indicated by gene ontology (GO) analysis for the terms “platelet activation” and “platelet degranulation.” These findings are in line with our previous report indicating that circulating miR-21 levels are dependent on platelets (23). The correlation between circulating miR-21 and mature TGF-β1 was confirmed by qPCR and ELISA analysis, respectively, in platelet-poor plasma (PPP) collected during the 2015 follow-up (*n* = 332) of the Bruneck Study (Figure 3C). Our previous studies have also identified miR-21 as a major platelet miRNA (23, 31). To confirm enrichment of this miRNA in megakaryocytes, argonaute 2 (Ago2) immunoprecipitation was performed in a human megakaryoblastic leukemia cell line (MEG-01). Here, enrichment of miR-21 in the Ago2 complex was found, along with 2 previously identified platelet-enriched miRNAs (Figure 3D) (25). This suggests a functional role for miR-21 in megakaryocytes and platelets. Altogether, analyses in a community-based cohort study and in MEG-01 cells imply a role of miR-21 in platelets.

### TGF-β1 is enriched in megakaryocytes, and plasma levels are dependent on platelets.

**Figure 4:**
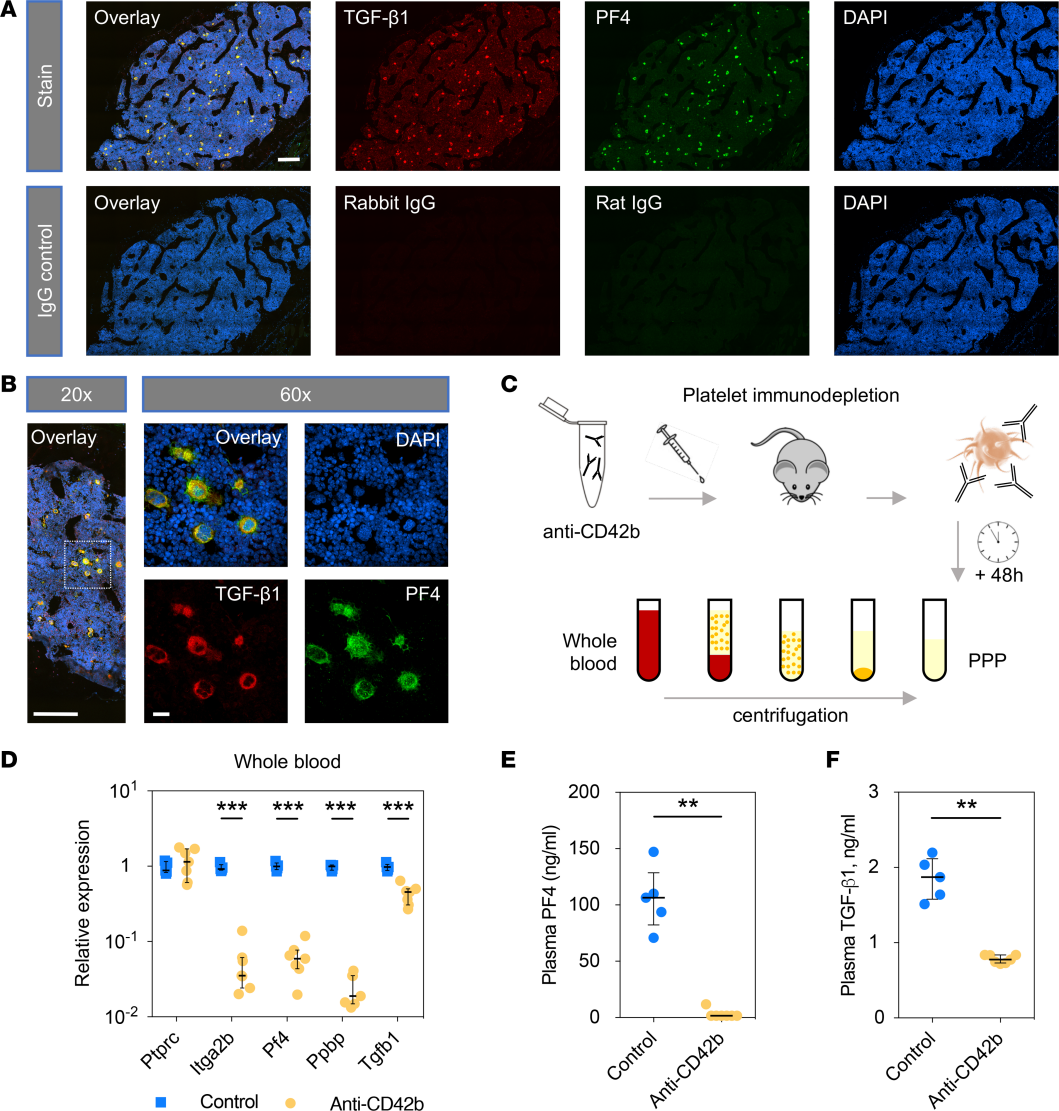
TGF-β1 in the bone marrow and circulation. (**A**) Transverse femoral sections from wild-type mice were used for immunohistochemical analysis of the bone marrow (*n* = 7; representative images shown). Sections were stained for TGF-β1 (red) and PF4 (green). DAPI was used as a nuclear counterstain (blue). Scale bar: 200 μm. (**B**) Images (original magnification, ×60) identifying the cellular and lobulated nuclear morphology characteristic of megakaryocytes. Scale bar: 200 μm and 20 μm for ×20 and ×60, respectively.(**C**) Wild-type mice were treated with a monoclonal antibody directed against GPIbα (anti-CD42b; 4 mg/kg intraperitoneally) to deplete platelets. Whole blood and platelet-poor plasma (PPP) were collected 48 hours after injection. (**D**) Expression levels of several platelet genes, as well as *Tgfb1*, were significantly lower in whole blood after platelet depletion. Expression of *Ptprc*, widely expressed in leukocytes, was not altered. *Gapdh* was used as reference gene transcript. Lines and error bars represent median (IQR). Statistical analysis was performed with Mann-Whitney test; *n* = 5 (control) versus 7 (anti-CD42b). (**E** and **F**) Effect of platelet depletion in mice on PPP levels of TGF-β1 and PF4 (Mann-Whitney test, *n* = 5 for control, *n* = 7 for anti-CD42b antibody-treated mice). Lines and error bars represent median (IQR).

Colocalization of TGF-β1 with PF4 was demonstrated by immunofluorescence staining of the murine bone marrow (Figure 4A). Further magnification of TGF-β1/PF4–positive cells showed large and lobulated nuclei characteristic of megakaryocyte morphology (Figure 4B). TGF-β1 staining beyond the areas of colocalization with PF4 was negligible, demonstrating that megakaryocytes are the major source for TGF-β1 in the bone marrow.

To study the dependency of plasma TGF-β1 levels on platelets, we used an antibody-mediated thrombocytopenia model in wild-type mice (Figure 4C) (32). Successful platelet depletion was confirmed by a significant reduction of platelet-specific gene transcripts (Figure 4D) in whole blood as well as by reduced levels of PF4 in PPP (Figure 4E). *Ptprc*, an abundant transcript in leukocytes encoding the cluster of differentiation 45 (CD45), was not affected. Importantly, levels of TGF-β1 in PPP were significantly decreased (median [interquartile range], 1.9 [1.6–2.1] and 0.78 [0.73–0.84] ng/ml after control or depletion, respectively; *P* = 0.0025; Figure 4F). These findings confirm platelets as the major source of TGF-β1 in the circulation.

### miR-21 affects the release of TGF-β1 from platelets.

**Figure 5:**
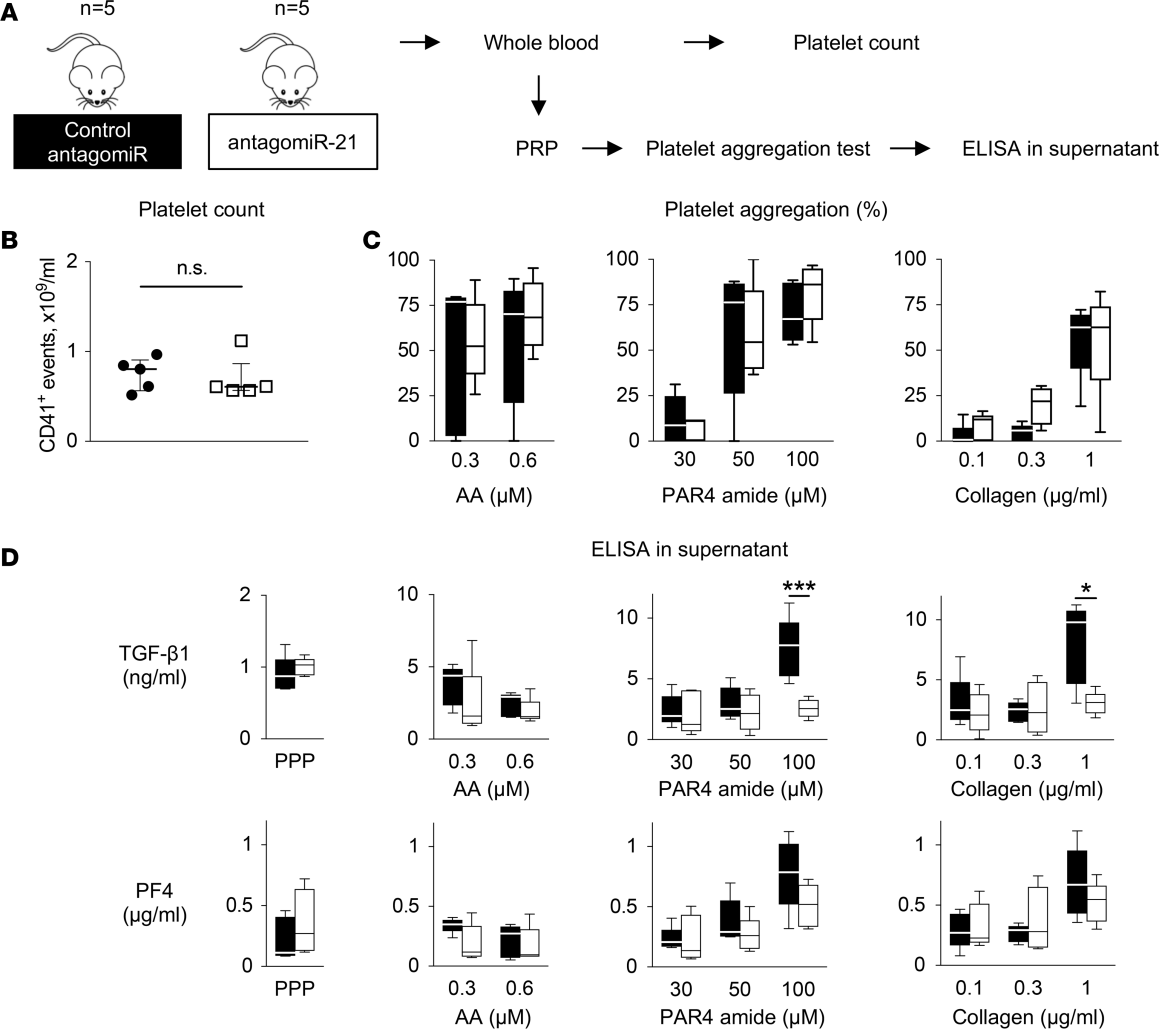
Pharmacological inhibition of miR-21 decreases platelet TGF-β1 release. (**A**) Platelet counts and their aggregation response were assessed in whole blood and platelet-rich plasma (PRP), respectively, isolated from mice treated with antagomiR-21 or control antagomiR. ELISAs were subsequently performed in the supernatant of aggregated platelets. (**B**) Platelet counts were assessed by flow cytometry using a CD41-allophycocyanin antibody on gated platelets. Statistical analysis was performed with Mann-Whitney test; *n* = 5 antagomiR-21–treated mice vs. *n* = 5 control antagomiR-treated mice. Lines and error bars represent median (IQR).(**C**) Platelet aggregation in response to arachidonic acid (AA), protease activated receptor 4 (PAR4) amide, or collagen did not show a significant difference after antagomiR-21 treatment (2-way ANOVA with post hoc Šidák’s test; *n* = 5 for each treatment type). (**D**) TGF-β1 release from platelets in response to collagen and PAR4 amide was reduced after treatment with antagomiR-21. PF4 was less affected. TGF-β1 levels in platelet-poor plasma (PPP) were unchanged. Statistical analysis was performed with 2-way ANOVA for agonists and Mann-Whitney test for PPP; *n* = 5 antagomiR-21–treated mice vs. *n* = 5 control antagomiR-treated mice for each condition; *P < 0.05; ***P < 0.001.

Next, we assessed the effect of miR-21 inhibition on platelets using antagomiR-21 treatment in mice (Figure 5A). First, platelet counts were determined by flow cytometry using allophycocyanin-CD41 staining (Supplemental Figure 11). No significant difference in platelet numbers was found between mice treated with antagomiR-21 or control (Figure 5B). miRNAs have previously been shown to alter platelet function (22, 24, 25). To determine whether miR-21 inhibition altered platelet activation, we measured the aggregation response to various agonists using a similar approach as that previously described for miR-126 (25). Unlike miR-126, pharmacological inhibition of miR-21 did not affect the platelet aggregation response to arachidonic acid, protease-activated receptor 4 (PAR4) amide, or collagen (Figure 5C). In light of the correlation between miR-21 and TGF-β1 levels in human plasma, we evaluated whether plasma levels and platelet release of TGF-β1 were affected by antagomiR-21 (Figure 5D). Interestingly, platelet TGF-β1 release was significantly reduced in response to PAR4 amide (*P* = 0.003) and collagen (*P* = 0.011). The release of PF4, a marker of platelet activation, was less affected. No differences were seen for levels of either protein in PPP.

### WASp is a direct target of miR-21.

**Figure 6:**
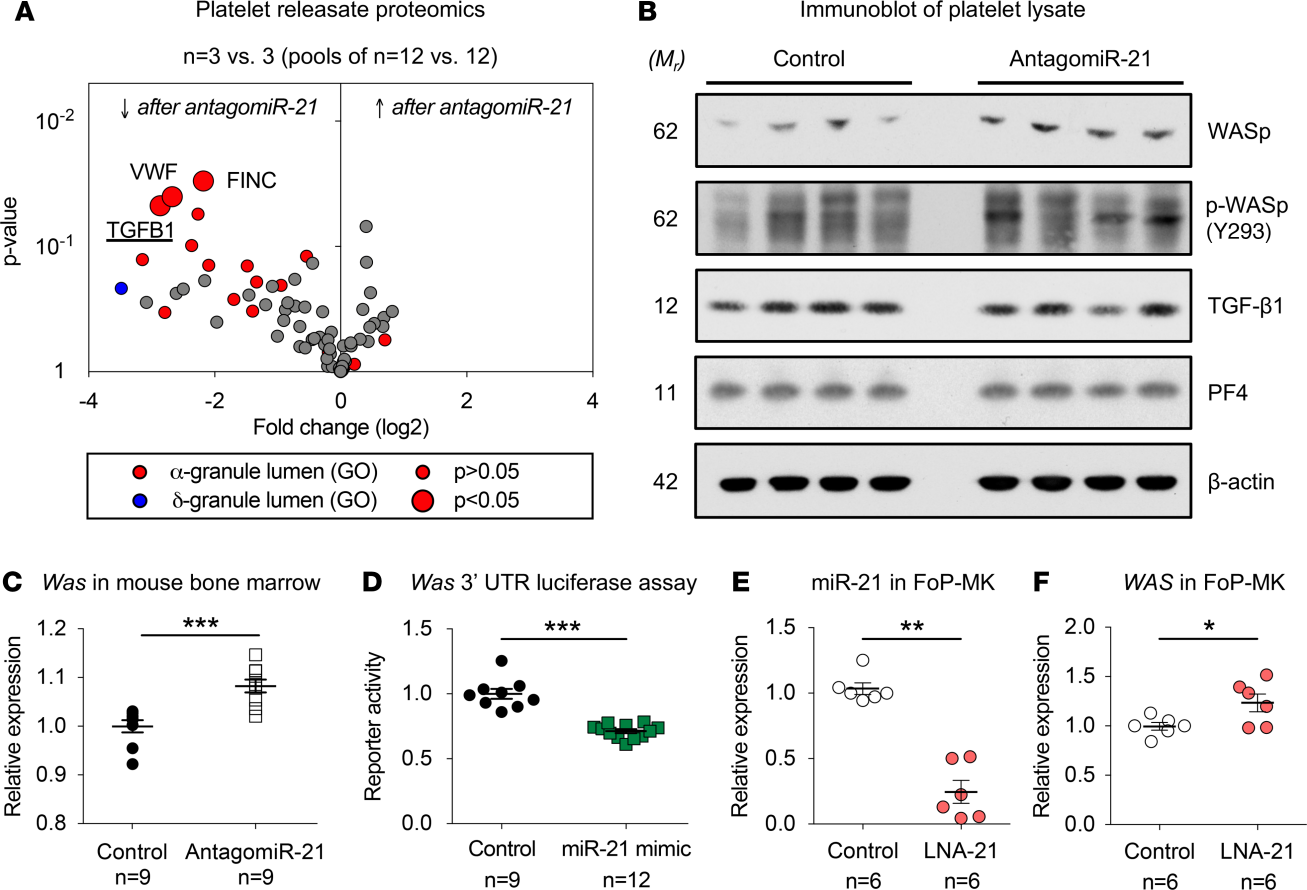
Pharmacological inhibition of miR-21 affects platelet TGF-β1 release through targeting Wiskott-Aldrich syndrome protein. (**A**) The platelet releasate was obtained by thrombin-mediated activation of washed platelets collected from antagomiR-21– or control-treated mice. Proteomics analysis uncovered an attenuated release of TGF-β1 and 2 other α-granule proteins, von Willebrand factor (VWF) and fibronectin (FINC), from platelets isolated after antagomiR-21 treatment. Statistical analysis was performed using Welch’s *t* test (*n* = 3 vs. 3 biological replicates; pooled from *n* = 12 antagomiR-21–treated mice and *n* = 12 control antagomiR–treated mice). (**B**) Platelets were isolated from mice after miR-21 inhibition. Immunoblots showed higher levels of Wiskott-Aldrich syndrome protein (WASp), while TGF-β1 content was unchanged (*n* = 4 antagomiR-21–treated mice vs. 4 control antagomiR–treated mice; statistical comparisons of densitometry using Welch’s *t* test). Similarly, WASp phosphorylation at tyrosine 293 (p-WASp; Y293) was unaffected.(**C**) Gene expression of *Was* in murine bone marrow after antagomiR-21 treatment was assessed by qPCR (*n* = 9 antagomiR-21–treated mice vs. 9 antagomiR control–treated mice; Welch’s *t* test). *Actb* was used as a normalization control. (**D**) Transfection of HEK293T cells with miR-21 mimic significantly suppressed luciferase reporter activity using vectors harboring the 3′ untranslated region of *Was* (3 independent experiments in triplicates [control] or quadruplicates [miR-21 mimic]; paired *t* test used for statistical comparison). (**E** and **F**) Megakaryocytes were produced by forward programming (FoP-MK) of human pluripotent stem cells (hPSCs). Upon transfection with a miR-21 inhibitor (LNA-21), miR-21 levels were markedly reduced. The inhibition of miR-21 was accompanied by increased gene expression levels of *WAS* (*n* = 6 LNA-control transfections vs. 6 LNA-21 transfections; Welch’s *t* test). *P < 0.05, **P < 0.01, ***P < 0.001. M_r_, relative mass.

Several platelet-derived factors, including TGF-β1, have previously been implicated in cardiac remodeling (19, 33). We therefore determined the effects of pharmacological miR-21 inhibition on the platelet protein releasate in mice. Platelets were isolated from mice treated with antagomiR-21 or control, aggregation was induced with thrombin, and the platelet releasate was used for proteomic analysis (Figure 6A). In agreement with the findings presented in Figure 5D, TGF-β1 was among the differentially regulated proteins after miR-21 inhibition (log_2_ FC = –2.86, *P* = 0.047). Other differentially regulated proteins were von Willebrand factor (VWF; log_2_ FC = –2.67, *P* = 0.040) and fibronectin (FINC; log_2_ FC = –2.18, *P* = 0.030). GO annotation identified all 3 proteins with presence in the α-granule lumen. Several other proteins with this GO term showed a similar trend of reduced release after antagomiR-21 treatment (Supplemental Table 5).

Platelet TGF-β1 release has previously been shown to be curtailed by WASp (17), an actin assembly protein selectively expressed in cells of hematopoietic origin (34). To evaluate levels of TGF-β1 and WASp by immunoblotting, platelets were isolated and lysed after antagomiR-21 or control treatment. While platelet levels of TGF-β1 (FC = 0.98, *P* = 0.909) and PF4 (FC = 1.05, *P* = 0.473) were unaltered upon pharmacological miR-21 inhibition, levels of WASp (FC = 1.90, *P* = 0.047) were significantly increased (Figure 6B and Supplemental Figure 12). Analysis of WASp phosphorylation at tyrosine 293 (Y293; p-WASp), which constitutes the active form of WASp that restricts platelet TGF-β1 release (17), showed a similar trend (FC = 1.49, *P* = 0.056). Phosphorylation did not differ after normalization for total WASp levels (Figure 6B and Supplemental Figure 12C). The effect on total WASp levels was confirmed by qPCR in the bone marrow of antagomiR-21–treated mice, showing increased *Was* gene expression levels (FC = 1.08; *P* < 0.001) (Figure 6C). Target prediction algorithms have a high false-positive rate (35), with a combination of commonly used algorithms yielding over 14,000 putative genes for canonical miR-21 targeting (36). A luciferase reporter assay was therefore used to evaluate targeting of *Was* by miR-21. This indeed suggested direct targeting (FC = 0.71; *P* = 0.003) (Figure 6D). To assess whether miR-21 inhibition can affect the expression of *WAS* in human megakaryocytes, forward-programmed human pluripotent stem cell–derived (hPSC-derived) megakaryocytes (FoP-MKs) were used (37). Two independent lines with >95% megakaryocyte purity (Supplemental Figure 13A) were transfected with a nontargeting control or a miR-21 inhibitor, resulting in a significant decrease of miR-21 levels (Figure 6E). In line with our findings in murine bone marrow and platelets, as well as the luciferase assay for the murine *Was* 3′ UTR, inhibition of miR-21 resulted in a concomitant increase of *WAS* expression levels (Figure 6F). No significant effect was seen on megakaryocyte purity or maturity (Supplemental Figure 13, B–D). Thus, our findings indicate that pharmacological miR-21 inhibition leads to derepression of WASp, which has previously been shown to affect the release of TGF-β1 from platelets (17).

### miR-21–null mice have a reduced number of circulating platelets.

**Figure 7:**
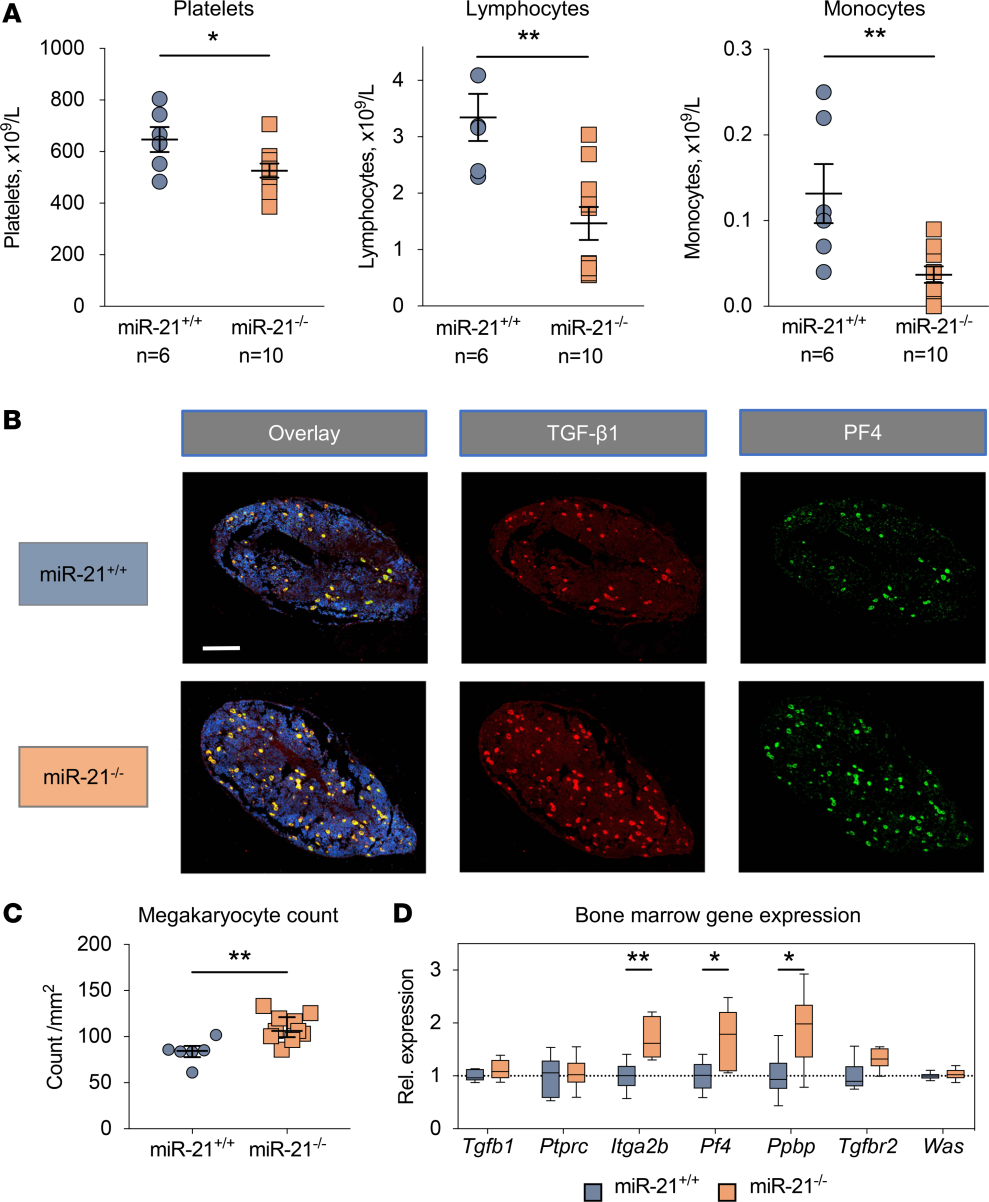
miR-21–null mice display lower platelet and leukocyte numbers but higher numbers of megakaryocytes. (**A**) Using Hemavet blood cell counting, significantly lower numbers of platelets, lymphocytes, and monocytes were found in miR-21–null mice (*n* = 6) compared with those in wild-type littermates (*n* = 10) (Welch’s *t* test). (**B**) Immunohistochemical analysis was performed for TGF-β1 (red), PF4 (green), and a nuclear counterstain (DAPI, blue), using femoral bone marrow sections of miR-21–null mice (*n* = 10). Representative images are shown. (**C**) Examination of bone marrow sections from miR-21–null mice revealed increased megakaryocyte numbers, as identified based on morphology and PF4 positivity. Statistical comparison was performed using the Mann-Whitney test; *n* = 6 vs. 10 for littermate control and miR-21–null mice, respectively. Lines and error bars represent median (IQR). (**D**) qPCR analysis revealed increased expression levels of megakaryocyte-specific genes *Itga2b*, *Pf4*, and *Ppbp* in bone marrow cells isolated from miR-21–null and littermate control mice. Transcript levels of *Tgfb1*, *Was*, and *Ptprc* (CD45) were not affected. Statistical comparisons using Welch’s *t* test; *n* = 6 vs. 6. *P < 0.05, **P < 0.01.

Previous studies into miR-21 and cardiac fibrosis have highlighted a discrepancy between pharmacological and genetic inhibition of this miRNA (2, 3). miR-21–null mice were reported to develop fibrosis in response to myocardial infarction or pressure overload that is equivalent to that of wild-type mice. ELISA measurements for TGF-β1 and PF4 in PPP showed no significant difference between wild-type and miR-21–null mice. Similarly, platelet levels of WASp and TGF-β1 were also unaffected (Supplemental Figure 14). To assess other potential changes in platelets from miR-21–null mice, we performed a whole-blood platelet count using a Hemavet hematology analyzer. In contrast to short-term pharmacological inhibition of miR-21, blood from miR-21–null mice showed significantly lower platelet counts compared with wild-type littermates (Figure 7A). Lymphocyte and monocyte numbers were also reduced but still fell within the normal reference range. To determine the megakaryocyte response to the lower platelet count, immunohistological staining of the bone marrow was performed in decalcified femoral sections (Figure 7B). A significant increase in megakaryocyte numbers was seen in miR-21–null bone marrow, with a median (interquartile range) count per mm^2^ of 84.7 (78.0–90.0) and 106.3 (99.5–121.2) for wild-type and miR-21–null mice, respectively (*P* = 0.003 for Mann-Whitney test) (Figure 7C). We next studied gene expression levels in the bone marrow of these mice (Figure 7D). No significant differences were seen for expression of *Tgfb1*, *Ptprc* (CD45), or *Was*. However, transcript levels of several megakaryocyte-specific genes were significantly higher (FC = 1.70 [*P* = 0.005] for *Itga2b*, 1.72 [*P* = 0.026] for *Pf4,* 1.90 [*P* = 0.030] for *Ppbp*). Furthermore, there was a trend toward higher expression levels for *Tgfbr2* (FC = 1.32, *P* = 0.055), a previously described direct target of miR-21 (38). Altogether, platelet and megakaryocyte numbers are altered in miR-21–null mice.

## Discussion

In this study, we identify a potential novel mechanism for the antifibrotic effects of miR-21–targeted therapy: notably, miR-21 minimally affects ECM protein secretion by CFs in vitro and in miR-21–null mice. Instead, miR-21 levels correlate with several platelet-derived profibrotic factors in the circulation, and its genetic ablation decreases platelet counts. Short-term pharmacological miR-21 inhibition attenuates the release of TGF-β1 from platelets. This suggests that pharmacological inhibition of miR-21 acts systemically in addition to its effects on CF proliferation. On the other hand, genetic deletion of miR-21 reduces platelet numbers and stimulates megakaryocyte proliferation. These findings may have implications for miR-21–based therapeutics, in particular with regard to local versus systemic delivery.

### Direct effects of miR-21 in the heart.

There is strong evidence for the contribution of miR-21 in fibrosis. Systemic inhibition could prevent and even reverse cardiac fibrosis in a pressure-overload model (3), which was confirmed in most (4, 5), but not all (2), studies. Fibroblasts are the primary contributors to fibrosis (39, 40), with the TGF-β pathway playing a central role in fibroblast activation. Despite enhancing CF proliferation, miR-21 had no major effect on cardiac ECM protein secretion. These findings are in stark contrast with previous studies that revealed major changes in ECM protein secretion using a similar approach for miR-29b (15) and hypoxia (41). Experiments in CFs were performed based on equal cell numbers prior to transfection and TGF-β1 treatment. Hence, it can be stated that, despite enhancing proliferation, this antiproliferative effect of miR-21 inhibition was insufficient to cause detectable changes in total ECM protein secretion. Previous discrepancies between studies on miR-21 and cardiac fibrosis have been ascribed to differences between pharmacological and genetic miR-21 inhibition (42). In line with our in vitro findings and with functional cardiac analysis by others (2), the composition of the cardiac ECM did not significantly differ in miR-21–null mice. A recent study by Ramanujam et al. used viral vectors with different tropism to distinguish effects of miR-21 inhibition in different cardiac cell types (26). While cardiomyocyte inhibition of miR-21 aggravated fibrosis, nonmyocyte targeting attenuated remodeling in a mouse model of cardiac overload. Importantly, effects of miR-21 on cardiac fibrosis are observed only in the presence of tissue injury (3–6).

### miR-21 as a modulator of the bone marrow.

miR-21 is ubiquitously expressed and has been implicated in fibrosis in different organs (7–9). Furthermore, miR-21 is highly abundant in circulating cells, such as monocytes and platelets (43). Recent studies on atherosclerosis identified an immunomodulatory role of miR-21 in mononuclear cells (12, 30, 44). Leukocytes and platelets are major contributors to the tissue injury response (45). Thus, the widely reported induction of miR-21 upon injury may be secondary to an immune cell infiltration or platelet deposition rather than induction of miR-21 expression in resident cells (46). In addition, expression and processing of the miR-21 precursor is enhanced by TGF-β signaling (47). A platelet contribution may explain why miR-21 inhibition has no effect on fibrosis in the absence of tissue injury. Tissue injury induces platelet activation. In addition to local fibroblast activation, platelet-derived proteins (33), including TGF-β1 (19), contribute to the onset and progression of fibrosis. Platelet inhibition or depletion attenuates cardiac remodeling after myocardial infarction (48, 49) or in response to pressure overload (18, 33). We have previously demonstrated that miR-21 is abundant in platelets and miR-21 plasma levels are decreased by antiplatelet therapy (23). Ago2 immunoprecipitation showed marked enrichment of miR-21 in a human megakaryoblastic leukemia cell line. Bone marrow cells are more readily affected by antagomiR treatment compared with cardiac tissue (50). This is in contrast with the absence of miR-21 across tissues upon genetic ablation in mice. In miR-21–null mice, we found no major effect on cardiac ECM proteins, but we observed significantly lower platelet and leukocyte counts, accompanied by a marked increase in megakaryocyte numbers. Altogether, these findings imply that miR-21 inhibition alters the bone marrow.

### Pharmacological miR-21 inhibition affects the platelet releasate.

Platelets constitute a major source of circulating TGF-β1 (19). This is reflected in the strong correlation between plasma levels of miR-21 and TGF-β1 in the Bruneck cohort and by the reduction in TGF-β1 plasma levels upon platelet depletion in mice. In the bone marrow of mice, we observed a striking colocalization of TGF-β1 with megakaryocytes. Pharmacological inhibition of miR-21 did not alter the platelet count or their aggregation response but significantly decreased the release of several α-granule proteins. Our observation of miR-21 inhibition affecting platelet TGF-β1 release suggests a feed-forward mechanism, where TGF-β1 induces miR-21 expression in megakaryocytes, which subsequently enhances platelet TGF-β1 release through inhibiting WASp. WASp has been described as a regulator of actin polymerization in neutrophils, macrophages, and lymphocytes (51), being expressed solely in hematopoietic cells (34). While WASp is present in platelets, it is not required for platelet spreading, despite the importance of cytoskeletal F-actin polymerization in this process (52). This indicates redundancy in the mechanisms that regulate actin polymerization in platelets. Consequently, miR-21–mediated titration of WASp levels may be a refined way of interfering with TGF-β signaling. Previous approaches of TGF-β1–targeting therapy have thus far failed due to major side effects (53). Altering the platelet release of profibrotic factors may provide a more targeted approach, as platelet activation occurs at sites of tissue injury. In summary, our findings provide evidence that the reported antifibrotic effects upon miR-21 inhibition may, at least in part, occur through decreasing platelet release of TGF-β1.

### Study limitations.

The culture of CFs for secretome analysis was performed in serum-free medium. This may have suppressed some of the effects of miR-21 in these cells. In addition, ECM proteins were assessed only in miR-21–null mice without cardiac injury to promote fibrosis. Correlations between miR-21 and markers of platelet activation or profibrotic factors do not rule out potential confounding by contaminating leukocyte subtypes. For TGF-β1, however, the immunohistochemistry results from the bone marrow indicate a high degree of specificity for megakaryocytes. Our findings suggest that antagomiR-21 treatment may reduce TGF-β1 release through derepressing WASp. While direct targeting was indicated by a luciferase reporter assay and by miR-21 inhibition in hPSC-derived megakaryocytes, in vivo evidence for this interaction is correlative. In addition, as suggested by the proteomic analysis of the platelet releasate, antagomiR-21 treatment may affect release of other α-granule proteins as well. Further studies are needed to determine whether miR-21 has effects on the release of other constituents of platelets and on leukocytes, given that WASp is predominantly expressed in hematopoietic cells and Wiskott-Aldrich syndrome is characterized by immune deficiency as well as thrombocytopenia (34).

### Conclusions.

This study is a timely contribution, given that miR-21–based therapies are progressing to clinical trials for Alport syndrome (7). The current assumption that miR-21 inhibition attenuates fibrosis predominantly through direct effects on fibroblasts is challenged by the marginal changes in ECM protein composition and secretion. Our findings after pharmacological and genetic miR-21 inhibition in mice indicate that other cell types, in particular bone marrow–derived cells, may also contribute. While local delivery of miRNA therapeutics may decrease the likelihood for off-target effects (10), it could also limit therapeutic efficacy if the benefit is not solely mediated by local cells. A broad appraisal of the multifaceted systemic response of targeting ubiquitously expressed miRNAs will aid the success of miRNA-based therapeutics in clinical trials.

## Methods

Detailed methods are provided in the Supplemental Methods.

### RNA isolation, reverse transcription, and PCRs.

RNA isolation, reverse transcription (RT), and preamplification as well as individual quantitative real-time PCR (qPCR) for miRNA and gene expression were performed as described previously (25). A comprehensive list of assays is provided in Supplemental Table 1.

### CF isolation and transfection for secretome analysis.

Primary mouse CFs were isolated from the hearts of male (8- to 10-week-old; *n* = 8) C57BL/6 mice (Charles River) by collagenase II–based digestion as described previously (15). Cells from different animals were maintained as separate biological replicates. Cells at passage 3 were used in all experiments. Transfections of miR-21 mimic (Dharmacon) or miR-21 inhibitor (LNA-21; Exiqon A/S) were each carried out, with corresponding controls in parallel using 4 separate biological replicates. Lipofectamine RNAiMAX (Life Technologies) was used in reduced serum medium (Opti-MEM, Life Technologies) at a final concentration of 50 nmol/l for transfection of all 4 sequences. After 24 hours, CFs were stimulated with 10 ng/ml recombinant TGF-β1 (Peprotech) in serum-free medium. After 48 hours, conditioned medium and cells were harvested for analysis. Samples were processed for qPCR analysis, immunoblotting, or proteomic analysis as described previously (15) and in the Supplemental Methods. See complete unedited blots in the supplemental material.

### Proliferation assay in transfected CFs.

A proliferation assay was performed using the xCELLigence RTCA DP Instrument (ACEA Biosciences). CFs were isolated and transfected using the same methods and concentrations as above. After 24 hours, cells (5 × 10^3^ cells/well; 4 biological replicates per condition, in duplicate) were seeded in an E-Plate VIEW 16 (ACEA Biosciences) in complete DMEM medium. Following plating, cells were allowed to attach while at room temperature for 30 minutes before placing the plate in the incubator at 37°C. Two wells per plate were loaded with media only to assess background levels. Cell index values were recorded by 150 sweeps per interval every 30 minutes using proprietary software, with the baseline recording reset at 4 hours. Data were analyzed using the difference (Δ) in cell index according to the manufacturer’s instructions with the baseline recording reset at 4 hours.

### ECM extraction and analysis.

ECM protein enrichment was performed on hearts from female miR-21–null mice or littermate controls, aged 14–16 weeks, using our previously published 3-step extraction method (28, 54). Generation of the miR-21–null mice has previously been described (2). Proteins in the sodium chloride and guanidine hydrochloride extracts were enzymatically deglycosylated, followed by denaturation, reduction, alkylation, and tryptic digestion. Peptides were purified using a C18 spin plate (Harvard Apparatus) and analyzed by LC-MS/MS. Identified proteins were annotated using the Matrisome database (29).

### Bruneck cohort.

The Bruneck Study is a community-based, prospective survey of the epidemiology and pathogenesis of atherosclerosis and cardiovascular disease (25, 55). At the 1990 baseline evaluation, the study population comprised an age- and sex-stratified random sample of all inhabitants of Bruneck (125 men and 125 women from each of the fifth through eighth decades of age, all White). In the present study, citrate plasma samples from the 2000 (*n* = 660) and 2015 (*n* = 332) follow-up were analyzed. These samples were drawn after an overnight fast and 12 hours of abstinence from smoking. During the 2000 follow-up, citrate plasma was prepared by single centrifugation and aliquots were immediately stored at –80°C. For the 2015 follow-up, platelet-rich plasma (PRP) was first isolated by centrifugation at 175 *g* for 15 minutes with slow brake. An aliquot of PRP was then supplemented with prostacyclin (PGI_2_, epoprostenol, 2 μg/ml; Tocris Bioscience) and centrifuged at 1,000 *g* for 10 minutes to obtain PPP. Samples were immediately stored at –80°C. Levels of miR-21 and a panel of 228 proteins were measured by RT-qPCR (25) and a combination of Olink Proseek Proximity Extension Assays (Proseek Multiplex CVD I and Inflammation panels, Olink) (55, 56) and mass spectrometry–based assays (PlasmaDive, Biognosys AG) (57), respectively, as described previously (25). In addition, TGF-β1 (DY240, R&D Systems), PF4 (DY795, R&D Systems), and PPBP (DY393, R&D Systems) levels were measured using DuoSet ELISA Development kits and DuoSet Ancillary Reagent Kits 1 and 2 (R&D Systems) according to the manufacturer’s instructions. Absorbance at 450 nm was measured on a plate reader (Tecan Infinite 200 Pro) using 570 nm as a reference wavelength. Results were calculated using a 4-parameter logistic fit.

### Platelet immunodepletion in mice.

Male C57BL/6J mice (Charles River) were treated with a rat anti-mouse GPIbα antibody (4 mg/kg, Emfret Analytics) or sterile PBS via intraperitoneal injection. Blood was collected after 48 hours into acid-citrate-dextrose buffer by cardiac puncture, followed by preparation of PRP and PPP using serial centrifugation at 100 *g* and 1,000 *g* for 10 minutes at room temperature, respectively. Samples were divided into aliquots, blinded, randomized, and immediately stored at –80°C. RNA isolated from whole blood was used for qPCR analysis. TGF-β1 (DY1679) and PF4 (DY595) levels were measured in PPP using DuoSet ELISA Development kits (R&D Systems) according to the manufacturer’s instructions.

### Ago2 immunoprecipitation.

Immunoprecipitation of ribonucleoprotein was carried out in MEG-01 cells (ATCC, CRL-2021), as previously described (25, 58). Briefly, 50–100 million cells were lysed in 500 μl ice-cold polysome lysis buffer (5 mM MgCl_2_, 100 mM KCl, 10 mM HEPES, pH 7.0, 0.5% Nonidet P-40), with freshly added 1 mM DTT, 100 U/ml RNase inhibitor (Life Technologies) and 1× protease inhibitor (cOmplete Mini, ethylenediaminetetraacetic acid–free [EDTA-free], Roche) for 15 minutes. Centrifugation was performed at 14,000 *g* at 4 °C for 10 minutes. The supernatant was mixed with 500 μl ice-cold NT2 buffer (50 mM Tris, pH 7.4, 150 mM NaCl, 1 mM MgCl_2_, 0.05% Nonidet P-40) containing freshly added 200 U/ml RNase inhibitor, 0.5% vanadyl ribonucleoside, 1 mM DTT, 15 mM EDTA, and 50 μl mouse anti-human Ago2-coated (Abcam) sepharose G beads (Life Technologies). Incubation was carried out overnight at 4°C on a rocking platform. The following day, beads were washed 5 times with ice-cold NT2 buffer and used for RNA isolation.

### Murine platelet and bone marrow analysis.

For pharmacological miR-21 inhibition, male C57BL/6J mice, aged 10–12 weeks (Charles River), were injected intraperitoneally with cholesterol-conjugated antagomiR constructs (Fidelity Systems) targeting miR-21 or using a nontargeting sequence, as previously described (25). Sequences were designed to target miR-21 (5′-U*C*AACAUCAGUCUGAUAAG*C*U*A*-Chol*T-3′) or to serve as nontargeting control (5′-A*A*GGCAAGCUGACCCUGAA*G*U*U*-Chol*T-3′), with the asterisks denoting phosphorothioate backbone modification and “Chol” indicating cholesterol conjugation. Constructs were reconstituted in sterile PBS, and intraperitoneal injections of 500 μl were performed on day 0, 1, and 2 in a dose of 25 mg/kg for the platelet releasate isolation and 40 mg/kg for the aggregometry experiments and the ELISA measurements in plasma. Treatment with both constructs was performed in parallel. On day 7, mice were anesthetized using pentobarbital for blood and tissue collection. Upon sample collection, samples were blinded and randomized prior to analysis. Blood and bone marrow samples were obtained from equal numbers of male and female miR-21–null mice (Mir21atm1Yoli/J, Jax 016856; The Jackson Laboratory) and littermate controls, aged 8–12 weeks. Mice were kept under constant temperature and humidity in a 12-hour-controlled dark/light cycle.

A Hemavet (Drew Scientific) blood cell counter was used for analysis of miR-21–null blood samples. Platelet counting after antagomiR-21 treatment was performed on diluted whole blood incubated with an allophycocyanin-labeled anti-mouse CD41 antibody (BioLegend), using an Accuri C6 flow cytometer (BD Biosciences). Light transmission aggregometry was performed with PRP as described previously (25). Platelets were isolated by blood collection into acid-citrate-dextrose buffer, followed by 2-step centrifugation and washing in modified Tyrode-HEPES buffer supplemented with prostaglandin E_1_. PPP was isolated by 2-step centrifugation, obtaining PRP followed by a second spin to obtain PPP. TGF-β1 and PF4 levels were measured in PPP and light transmission aggregometry supernatants using DuoSet ELISA Development kits (R&D Systems) according to the manufacturer’s instructions. Releasate was obtained by thrombin-induced platelet aggregation in platelets obtained from blood pooled from 4 mice, followed by gel-based LC-MS/MS analysis. Bone marrow cells were isolated from femora and filtered using a 100-μm cell strainer (Corning), followed by red blood cell lysis and subsequent RNA isolation for qPCR analysis. For immunohistochemistry, femora were fixed in 4% formaldehyde and then decalcified in a 0.38 mol/l EDTA solution at 4°C for 3 weeks. Bones were paraffin embedded and sectioned for staining. After primary antibody incubation (TGF-β1 and PF4; see Supplemental Table 2) overnight, sections were incubated with fluorescently labeled species-specific secondary antibodies. Staining was visualized using an inverted spinning disc confocal microscope (Nikon Ti-Eclipse, teamed with a Yokogawa CSU-X1 Andor Spinning Disc with iXon EM-CCD camera). Imaging was performed with a 1.70 NA ×20 or 1.40 NA ×60 magnification objective lens.

### Transfection of miR-21 inhibitor in human megakaryocytes.

Human megakaryocytes were obtained by forward programming of hPSCs as previously described (37). Transfections of miR-21 inhibitor or a nontargeting control (PowerLNA, Qiagen) were carried out in triplicate in 2 independent megakaryocyte lines. Conjugation to TurboFect reagent (Thermo Scientific) was used to enhance the transfection efficiency. A final concentration of 25 nmol/l was used for both oligonucleotides. After 48 hours, cells were collected for RNA isolation by centrifugation at 120 *g* and washed once in sterile PBS. Prior to transfection and during collection, an aliquot of cells was incubated with anti-CD41a-APC-H7 and anti-CD42a-APC (BD Pharmingen) to assess megakaryocyte purity and maturity, respectively, using a Gallios flow cytometer (Beckman Coulter).

### Statistics.

Gel-based MS data were quantified using normalized spectral counts. Qspec (59) was used to detect differential expression in the CF secretome analysis. Qspec utilizes a hierarchical Bayes estimation of a generalized linear mixed-effects model to share information across proteins, which increases the power to detect differential expression. The FDR was calculated using an Empirical Bayes method, and FDR < 5% was considered significant. All other statistical analyses were performed with Microsoft Excel (version 15.41) and GraphPad Prism (version 7.0d). Distribution of data was analyzed using the Shapiro-Wilk normality test, where *P* > 0.05 was considered to indicate normal distribution. When at least 1 sample group within an experiment showed nonnormal distribution, nonparametric tests were used for the analysis. For unpaired data, 2-tailed Welch’s *t* test or Mann-Whitney rank tests were performed for parametric and nonparametric distribution, respectively. For paired data, paired *t* tests or Wilcoxon’s matched-pairs signed-ranks tests were used, respectively. Where appropriate, a 2-way ANOVA was used, with post hoc analysis of individual effects by Šidák’s test. Time course data of the CF proliferation assay were analyzed using the nonparametric Friedman test with post hoc analysis of differences between each targeting and nontargeting transfection type using Dunn’s multiple comparisons test. Comparison of gene expression and ECM protein abundance between miR-21–null or wild-type mice was performed using an FDR approach with the 2-stage step-up method of Benjamini, Krieger, and Yekutieli, with an FDR for significant discovery set to 5%. Relationships between the concentrations of plasma proteins and the relative quantities of miRNAs were investigated using Pearson correlations with *P* value adjustment (producing *q* values) using FDR adjustment according to Benjamini, Krieger, and Yekutieli. Data are shown as mean ± SEM unless stated otherwise. Where box-and-whisker plots are shown, the line indicates the median, bounds of boxes indicate quartiles, and whiskers indicate the range. *P* < 0.05 or *q* < 0.05 was considered significant.

### Study approval.

The Bruneck study protocol was reviewed and approved by the ethics committees of Verona and Bolzano, Italy, and all participants provided written informed consent before entering the study. All animal studies were performed under protocols in strict accordance with the UK Animals (Scientific Procedures) Act 1986 or the Institutional Animal Care Use Committee of Yale University School of Medicine.

## Author contributions

TB was involved with the study design, performed and analyzed CF and miR-21–null sample experiments; collected and analyzed samples for the Bruneck 2015 evaluation; designed and analyzed samples from antagomiR treatment and platelet depletion experiments in mice; performed statistical analysis; and drafted the manuscript. SE designed and performed experiments in CFs for secretome and proliferation analysis. UM and ML performed antagomiR and platelet depletion experiments in mice. RL performed immunohistological staining and analysis and the Ago2 immunoprecipitation experiment. PCA and MVC performed functional analysis in murine platelets and supported the Bruneck 2015 evaluation sample collection. MS, MFF, and YS collected and analyzed blood and bone marrow samples from miR-21–null mice. TM and TB performed experiments in FoP-MKs. JBB supported the design and immunoblot experiments of CF secretome analysis and provided ECM expertise. ML and CS performed ECM and qPCR analysis in miR-21–null samples, respectively. XY and FB performed LC-MS/MS analysis. RP and SRL performed statistical analysis. AZ supported the experimental design and performed the luciferase reporter experiment. PS, MW, SK, and JW were involved in the design and running of the Bruneck study. RP, MS, and JG provided support in the miR-21–null mouse heart analysis. AMS, CG, TDW, and CFH secured funding and supported interpretation of the study. MM conceived and designed the analysis, secured funding, and edited the manuscript.

## Supplementary Material

Supplemental data

## Version 1 11/02/2018

Electronic publication
