# Supplementary material for: Inhibition of profibrotic microRNA-21 affects platelets and their releasate
Source: JCI Insight. 2018 Nov 2;3(21):e123335. doi: 10.1172/jci.insight.123335 (PMC6238735; doi:10.1172/jci.insight.123335)
Supplement: Supplemental data [file jciinsight-3-123335-s130.pdf]

ONLINE SUPPLEMENT

Inhibition of Pro-Fibrotic MicroRNA-21 Affects Platelets and their Releasate

Temo Barwari[1], Seda Eminaga[1], Ursula Mayr,[1] Ruifang Lu[1], Paul C. Armstrong[2], Melissa V. Chan[2], Mahnaz Sahraei[3], Marta Fernández-Fuertes[3], Thomas Moreau[4], Javier Barallobre-Barreiro[1], Marc Lynch[1], Xiaoke Yin[1], Christian Schulte[1], Ferheen Baig[1], Raimund Pechlaner[5], Sarah R. Langley[6,7], Anna Zampetaki[1], Peter Santer[8], Martin Weger[9], Roberto Plasenzotti[10], Markus Schosserer[11], Johannes Grillari[11], Stefan Kiechl[4], Johann Willeit[4], Ajay M. Shah[1], Cedric Ghevaert[4], Timothy D. Warner[2], Carlos Fernández-Hernando[3], Yajaira Suárez[3], Manuel Mayr[1]

From the [1] King's British Heart Foundation Centre, King's College London, London, United Kingdom; [2] Blizard Institute, Barts and The London School of Medicine & Dentistry, Queen Mary University of London, London, United Kingdom; [3] Department of Comparative Medicine and the Vascular Biology and Therapeutics Program, Yale University School of Medicine, New Haven, Connecticut, USA; [4] Department of Haematology, University of Cambridge, National Health Blood Service Centre, Cambridge, United Kingdom; [5] Department of Neurology, Medical University Innsbruck, Innsbruck, Austria; [6] Duke-NUS Medical School, Singapore; [7] National Heart Centre Singapore, Singapore; [8] Department of Laboratory Medicine, Bruneck Hospital, Bruneck, Italy; [9] Department of Internal Medicine, Bruneck Hospital, Bruneck, Italy; [10] Medical University of Vienna, Institute of Biomedical Research, Vienna, Austria; [11] Christian Doppler Laboratory on Biotechnology of Skin Aging, Department of Biotechnology, BOKU - University of Natural Resources and Life Sciences, Vienna, Austria.

Supplemental material

Supplemental Methods: **pp. 3-19**

Supplemental Figure 1-14: **pp. 20-29**

Supplemental Table 1-5: **pp. 30-39**

Supplemental Methods

MicroRNA transfection

Cells were plated at 60-70% confluency on the day before transfection. miR-21 (5'-UAGCUUAUCAGACUGAUGUUGA-3') and a matching control (sequence not specified) were synthesized by Dharmacon. For miR-21 inhibition, Locked Nucleic Acid (LNA)-modified sequences were synthesized by Exiqon (LNA-21: 5'-CAACATCAGTCTGATAAGCT-3'; LNA-control: 5'-TAACACGTCTATACGCCCA-3'; Exiqon A/S). Transfections were carried out using Lipofectamine RNAiMAX (Life Technologies) in reduced serum medium (Opti-MEM, Life Technologies), according to manufacturer's instructions, at a final concentration of 50 nM for all four sequences. Efficiency of transfection was tested using a fluorescently labelled control miRNA (miRIDIAN microRNA Mimic Transfection Control with Dy547, Dharmacon, GE Healthcare), showing intracytoplasmatic localization by fluorescence microscopy after 24 h (**Supplemental Figure 1**). The day following transfection, CFs were carefully washed in serum-free medium and then stimulated with recombinant TGF-β1 (Peprotech) at a concentration of 10 ng/ml in serum-free medium. After 48 h, conditioned medium and RNA were harvested for analysis.

Conditioned media processing for analysis

The conditioned media were collected and centrifuged at 3,000 x *g* for 10 min to remove cell debris. The supernatant was desalted using Zeba Spin desalting columns (Thermo Scientific), concentrated using filter columns with a 3 kDa cutoff (Millipore) and

precipitated with 100% ethanol. The precipitate was vacuum-dried and then resuspended in 60 µL of deglycosylation buffer (50 mM sodium acetate, 50 mM Tris, pH 6.8, 25 mM EDTA), containing N-glycosidase F (1:200), β-1,4 galactosidase (1:200), β-N-acetylglucosaminidase (1:200), endo-α-N-acetylgalactosaminidase (1:200), recombinant α-2-3,6,8,9-neuraminidase (1:200), chondroitinase (1:100), heparanase (1:500) and endo-β-galactosidase (keratinase; 1:500). Samples were deglycosylated by incubation at 25°C for 2 h followed by 37°C for 48 h in agitation. After adding 30 µl of sample loading buffer (100 mM Tris pH 6.8, 40% glycerol, 0.2% SDS, 2% β-mercaptoethanol and 0.02% bromophenol blue), 45 µl of sample were used for SDS-PAGE for subsequent mass spectrometry (MS) analysis, and 55 µl for immunoblotting.

Cardiac extracellular matrix protein enrichment

Hearts were obtained from miR-21 null mice and wild-type littermates (n=6 vs. 6, female, aged 14-16 weeks) housed at Vienna Medical University, Vienna, Austria. Samples were blinded and randomized prior to analysis. 50 mg of ventricular heart tissue per sample were diced and immediately placed in ice-cold PBS with 25 mM EDTA to remove plasma contaminants. Protease inhibitor cocktail (Roche) was added to all buffers. After five washes, diced tissue pieces were incubated with 500 µl 0.5 M NaCl buffer (0.5 M NaCl, 10 mM Tris, 25 mM EDTA, pH 7.5). Samples were gently vortexed for 1 h at room temperature. NaCl buffer was then removed and stored for later analysis. Subsequently, tissue samples were incubated with 500 µl 0.1% SDS buffer (0.1% SDS, 25 mM EDTA) at room temperature for 16 h with gentle vortexing. SDS solution was then removed and stored for later use. Finally, the samples were briefly washed in PBS and then placed in

250 µl guanidine hydrochloride (GuHCl) buffer (4 M GuHCl, 50 mM sodium acetate, 20 mM EDTA, pH 5.8). Incubation was performed for 48 h at room temperature and vortexed vigorously. GuHCl extracts were then collected and stored until later analysis. Protein concentrations in all three fractions were then determined by Bradford assay (BioRad) according to manufacturer's instructions. Proteins in the NaCl and GuHCl fractions were then prepared for MS analysis as follows: 15 µg of protein were precipitated by adding a ten-fold volume of 100% ethanol to GuHCl samples and 100% acetone to NaCl samples, followed by overnight incubation at -20°C. Proteins were then precipitated with centrifugation at 16000 x *g* for 40 min at 0°C. Protein precipitates were fully dried using a SpeedVac Concentrator (ThermoFisher Scientific). Protein pellets were then resuspended in 20 µl deglycosylation buffer as described earlier, but without N-glycosidase F. Samples were briefly vortexed and centrifuged, followed by incubation at 25°C for 2 h and 37°C for 24 h. Samples were then centrifuged and dried using the SpeedVac Concentrator, followed by resuspension in 20 µl of 1:100 N-glycosidase F in $H_2{}^{18}O$. After incubation at 37°C for 48 h, samples were processed for HPLC-MS/MS analysis by in-solution digestion. Identified proteins were annotated using the Matrisome database (1).

Gel-LC-MS/MS

Samples were denatured with sample loading buffer and incubation at 96°C for 5 min. Gel separation was performed using Bis-Tris discontinuous 4-12% polyacrylamide gradient gels (NuPage, Invitrogen) as described previously (2). Gels were fixed and silver-stained to visualize proteins. Each gel lane was divided into 12 pieces without leaving any

gap in between, followed by de-staining and in-gel tryptic digestion using an Investigator ProGest (Digilab) robotic digestion system. Eluted peptides were lyophilized under vacuum at -55°C for approximately 5 h (Christ Alpha 1-2 LD Freeze Dryer) and resuspended in 40 µl of 2% acetonitrile, 0.05% trifluoroacetic acid (TFA) in H_2O. Tryptic peptides were separated on a reversed phase nanoflow HPLC system (Dionex PepMap C18, 25cm x 75µm, Dionex Ultimate3000 RSLCnano) and eluted with a 70-min gradient (0-3 min, 2-10% B; 3-37 min, 10-35% B; 37-40 min, 35-40% B; 40-50 min, 99% B; 50-70 min, 2% B; where A=0.1% formic acid in HPLC-grade H_2O and B=80% acetonitrile, 0.1% formic acid in HPLC-grade H_2O). The sequentially eluted peptides were directly analyzed by an Orbitrap mass analyzer (LTQ Orbitrap XL, Thermo Scientific) using full ion scan mode over the mass-to-charge (m/z) range of 400-1600, resolution 60000 (at m/z 400). Tandem MS (MS/MS) was performed using collision-induced dissociation (CID) in ion trap on the 6 most abundant ions in each full MS scan with dynamic exclusion. Raw files were searched against UniProt/SwissProt mouse and bovine databases (2015_02, 22689 protein entries), using Mascot software (version 2.3.01, Matrix Science). A mass tolerance of 10 ppm was selected for the precursor ions and at 0.8 Da for fragment ions. Carbamidomethylation of cysteine was set as a fixed modification and oxidation of methionine, proline and lysine as variable modifications. Two missed cleavages were allowed. Search results were loaded into Scaffold (Proteome Software Inc., version 4.3.2) to validate the MS/MS-based peptide and subsequent protein identification and to calculate the spectral count (3, 4). Peptide identifications were accepted if they could be established at a probability >95% as specified by the Peptide Prophet Algorithm (3). Only

tryptic peptides were included in the analysis. Protein identifications were accepted if they could be established at a probability >99% with at least two unique peptides (4).

In-solution digestion and HPLC-MS/MS analysis

Deglycosylated GuHCl/NaCl extracts were denatured by the addition of 9 M urea, 3 M thiourea in a 1:2 ratio (final concentration 6 M urea, 2 M thiourea). Samples were then reduced by adding 100 mM dithiothreitol (DTT; final concentration 10 mM) followed by incubation at 37°C for 1 h. Samples were then alkylated using 500 mM iodoacetamide (IAA, final concentration 50 mM) followed by incubation in the dark for 1 h at room temperature. Proteins were then precipitated using pre-chilled acetone in a 6:1 volume ratio with overnight storage at -20°C. Dried protein pellets were obtained as described in the previous section. For protein digestion, samples were resuspended in 30 µl trypsin solution, containing 0.01 µg/µl trypsin in 0.1 M triethylammonium bicarbonate (TEAB), pH 8.2. Proteins were digested overnight at 37°C under agitation (240 rpm). The digestion was stopped by acidification of the samples with 10% v/v trifluoroacetic acid (TFA; final concentration 1%). Peptide samples were purified using a 96-well C18 spin plate (MicroSpin, Harvard Apparatus) according to manufacturer's instructions. Dried peptide samples were then resuspended in 0.05% TFA in 2% ACN and separated on a nano-flow LC system (Dionex UltiMate 3000 RSLC-nano). Samples were injected onto a trap column (Acclaim® PepMap100 C18 Trap, 5mm x 300µm, 5µm, 100Å), at a flow rate of 25 µL/min for 3 min, using 2% ACN, 0.1% FA in H_2O. The following nano-LC gradient was then used to separate the peptides at 0.3 µL/min: 0−10 min, 2-10% B; 10−200min, 10-30% B; 200−210min, 30−40% B; 210-220min, 99%B, 220-250min 2%B, where A=0.1%

formic acid (FA) in H_2O, B=80% ACN, 0.1%FA in H_2O. The nano-column (Acclaim® PepMap100 C18, 50 cm x 75 µm, 3µm, 100 Å) was set at 40°C and coupled to a nanospray source (Picoview, New Objective). Spectra were collected from a Q Exactive Plus (Thermo Fisher Scientific) using full MS mode (resolution of 70,000 at 200 m/z) over the mass-to-charge (m/z) range 350–1600. Data-dependent MS2 scan was performed using the top fifteen ions in each full MS scan (resolution of 17,500 at 200 m/z) with dynamic exclusion enabled. Thermo Scientific Proteome Discoverer software (version 2.1.1.21) was used to search raw data files against the human database (UniProtKB/Swiss-Prot version 2017_02, 17,511 protein entries) using Mascot (version 2.6.0, Matrix Science). The mass tolerance was set at 10 ppm for precursor ions and 20 mmu for fragment ions. Trypsin was used as the enzyme with up to two missed cleavages being allowed. Carbamidomethylation of cysteine was chosen as a fixed modification; oxidation of methionine, lysine and proline, and deamidation of asparagine in the presence of ^{18}O water were chosen as variable modifications. Scaffold (version 4.3.2, Proteome Software Inc.) was used to validate MS/MS-based peptide and protein identifications with the following filters; a peptide probability of greater than 95.0% (as specified by the Peptide Prophet algorithm), a protein probability of greater than 99.0%, and at least two independent peptides per protein. The normalized total precursor intensity was used for quantification.

RNA isolation

Total RNA was extracted using the miRNeasy Mini kit (Qiagen) according to the manufacturer's recommendations, with some modifications. For isolation from heart

tissue, a 1-2 mm piece was cut from the apex. The tissue piece was directly placed into a tube containing Lysing Matrix D beads (MP Biomedicals) and 700 µl QIAzol reagent. Lysis was performed in a FastPrep-24 Homogeniser (MP Biomedicals) at 6000 rpm for two rounds of 20 sec. Cells or 50 µl of whole blood were lysed in 700 µl of QIAzol reagent. For RNA isolation from plasma, samples were centrifuged for 10 min at 4000 x *g* at 4°C. 500 µl of QIAzol reagent were then combined with 100 µl of plasma. Upon vigorous mixing and incubation at room temperature, 200 µl of spiking mixture (4 µl of 25 amol/L exogenous cel-miR-39-3p RNA [Qiagen], 1.25 µl of carrier RNA from bacteriophage MS2 [Roche] and 194.75 µl of QIAzol reagent). Upon vigorous mixing and incubation, lysed cells, tissue, blood or plasma were combined with 140 µl of chloroform and the solution was mixed vigorously. Samples were then centrifuged at 12,000 x *g* for 15 min at 4°C. 280 µl of upper (aqueous) phase were carefully mixed with 420 µl of 100% ethanol and then applied to columns and washed according to the manufacturer's protocol. Total RNA was eluted in 35 µl of nuclease-free H_2O by centrifugation at 9000 x *g* for 1 min at 4°C. Concentration of cellular and tissue RNA was determined by spectrophotometry based on absorbance at 260 nm using NanoDrop 2000c (Thermo Scientific).

Reverse transcription and pre-amplification

For relative quantification of miRNA in plasma by qPCR, 100 ng of RNA (or 3 µl for plasma RNA) were used as input in each reverse transcription (RT) reaction. RT reactions (and a pre-amplification step for plasma RNA) were set up according to the company's recommendations. Briefly, miRNAs were reverse-transcribed using the High Capacity cDNA Reverse Transcription Kit (Applied Biosystems), combining 100 ng RNA in 3.5 µl,

1 µl 10x RT Buffer, 0.3 µl 100 mM dNTPs with dTTP, 1.2 µl 25 mM $MgCl_2$, 0.2 µl RNase Inhibitor, 1 µl of 10x Megaplex RT Primers (Rodent Pool A v2.0 or Human Pool A v2.1, Applied Biosystems), 2 µl Multiscribe Reverse Transcriptase and 0.8 µl of nuclease-free H_2O. The RT-PCR reaction was set up as follows: 16°C for 2 min, 42°C for 1 min and 50°C for 1 sec for 40 cycles and then incubation at 85°C for 5 min using a Veriti Thermal Cycler (Applied Biosystems). Pre-amplification was performed using Megaplex PreAmp Primers (Human Pool A v2.1), using 1 µl of RT product, 5 µl Pre-amplification Mastermix (2x), 3 µl nuclease-free H_2O and 1 µl of Megaplex PreAmp Primers (10x). The pre-amplification reaction was performed by heating the samples at 95°C for 10 min, followed by 12 cycles of 95°C for 15 sec and 60°C for 4 min. Finally, samples were heated at 95°C for 10 min to ensure enzyme inactivation. Pre-amplification reaction products were diluted to a final volume of 40 µl. For gene expression levels in CF, bone marrow or cardiac RNA, RT was performed using the SuperScript VILO cDNA Synthesis Kit (Invitrogen). Per sample, 2 µl of VILO RT Master Mix were combined with 8 µl of sample in a 25-100 ng/µl dilution. Thermal cycler stages were set as follows: incubation at 25°C for 10 min and 42°C for 120 min, followed by termination of the reaction at 85°C for 5 min. RT-PCR and pre-amplification products were stored at -80ºC.

Real-time PCR

TaqMan miRNA or gene expression hydrolysis assays (**Supplemental Table 1**) were used to assess their relative expression levels, as previously described (5). 2.25 µl of diluted RT or pre-amplification product were combined with 0.25 µl of Taqman miRNA Assay (20X) (Applied Biosystems) and 2.5 µl of the Taqman Universal PCR Master Mix

No AmpErase UNG (2X) to a final volume of 5 µl. Reactions were loaded using a Bravo Automated Liquid Handling Platform (Agilent). qPCR was performed on a ViiA7 Real-Time PCR System (Applied Biosystems) at 95ºC for 10 min, followed by 40 cycles of 95ºC for 15 sec and 60ºC for 1 min. Relative quantification was performed using the $2^{-\Delta\Delta Cq}$ method with proprietary Viia7 software (Applied Biosystems). For cellular and tissue RNA, the reference gene transcript was selected based on analysis of stability using the online RefFinder tool (6). For plasma RNA, the exogenous cel-miR-39-3p spike-in was used as the reference transcript.

Immunoblotting

Samples were mixed with 4X denaturing sample buffer, heated at 95°C for 10 min and separated on Bis-Tris discontinuous 4-12% polyacrylamide gradient gels (NuPAGE, Invitrogen). Proteins were transferred to nitrocellulose membranes. Membranes were blocked with 5% fat-free milk powder in PBS containing 0.1% Tween20 (PBS-T) and probed overnight at 4ºC with primary antibodies (**Supplemental Table 2**) in 5% bovine serum albumin in PBS-T at 4°C while shaking. The membranes were incubated with light chain-specific secondary horseradish peroxidase (HRP)-conjugated antibodies (**Supplemental Table 2**) in 5% fat-free milk powder in PBS-T for 1 h. For analysis of phosphorylated Wiskott-Aldrich Syndrome protein (p-WASp), PBS-T was replaced with Tris-Buffered Saline with 0.1% Tween20 (TBS-T), with blocking and primary/secondary antibody incubation being performed using 5% bovine serum albumin in TBS-T. Blots were imaged using enhanced chemiluminescence (ECL, GE Healthcare) on a Xograph processor. Densitometry was performed using ImageJ software (v.1.48v, NIH, USA).

Platelet counting

For flow cytometry-based platelet counting, ACD-anticoagulated blood was diluted 25x with modified Tyrode-HEPES buffer at 37ºC. Samples were incubated with 5 µl of 0.2 mg/ml allophycocyanin (APC)-labelled anti-mouse CD41 antibody in the dark for 15 min at room temperature, followed by further dilution to 1:1000 with modified Tyrode-HEPES buffer. Unstained control samples were prepared by treatment with an equal amount of modified Tyrode-HEPES buffer. Samples were analyzed using an Accuri C6 flow cytometer (BD Biosciences) with slow flow. A set volume of each sample was analyzed to allow for absolute concentration calculation. Events corresponding to platelets were identified according to forward (FSC) and side scatter (SSC) as well as by identification of APC-positive events. Subsequent quantification of events was performed post-acquisition using FlowJo v7.4 (Tree Star). For miR-21 null mice and littermates, blood cell counting was performed using a Hemavet 950FS (Drew Scientific) using proprietary software according to manufacturer's instructions.

Platelet function testing

Blood was collected from the inferior vena cava using syringes containing lepirudin (Refludan, 25 µg/ml; Celgene) for aggregometry experiments and ELISA measurements. PRP was isolated as previously described (7). Briefly, whole blood was diluted 1:1 with modified HEPES-Tyrode's buffer (137 mM NaCl, 20 mM HEPES, 5.6 mM glucose, 1 g/l BSA, 1 mM $MgCl_2$, 2.7 mM KCl, 3.3 mM NaH_2PO_4) before centrifugation (100 x *g*, 8 min, room temperature). Platelet function tests were carried out as described previously (8). Half-area 96-well microtiter plates (Greiner Bio-One) were pre-coated with hydrogenated

gelatin (0.75% w/v; Sigma-Alrich) in PBS to block non-specific activation of blood. 4 µl of vehicle or agonist solution was then added to each well: arachidonic acid (AA; 0.03–0.6 mM; Sigma-Aldrich), Horm collagen (0.1–3 µg/ml; Nycomed) and the PAR-4 activating peptide AYPGKF amide (PAR4-AP, 30–100 µM; Bachem). To each well, 35 µl of PRP or whole blood was added and the plate was then placed onto a heated plate shaker (Bioshake IQ, Q Instruments) at 37°C for 5 min mixing at 1200 rpm. Light transmission of each well was determined using a 96-well plate reader (SunriseTM, Tecan) at 595 nm.

Platelet releasate isolation and analysis

Blood was collected into Acid-Citrate-Dextrose (ACD; 85 mM trisodium citrate dehydrate, 66.6 mM citric acid monohydrate, 111 mM anhydrous D-glucose) by cardiac puncture and pooled per four mice, followed by centrifugation at 150 x *g* for 10 min at room temperature. PRP was carefully transferred and combined with 10 µM indomethacin (Sigma-Aldrich) and 1 µM prostaglandin E_1 (PGE_1, Sigma-Aldrich). Platelet pellets were obtained by centrifugation of PRP at 500 x *g* for 10 min at room temperature. Supernatant PPP was collected, and the platelet pellet was washed twice with modified Tyrode-HEPES buffer (NaCl 134 mM, KCl 2.9 mM, Na_2HPO_4 0.34 mM, $NaHCO_3$ 12 mM, HEPES 20 mM, $MgCl_2$ 1 mM, Glucose 5 mM), supplemented with 1 µM PGE_1 and 10 µM indomethacin, with centrifugation at 500 x *g* for 10 min at room temperature in between. The platelet pellet was then resuspended in 200 µl of modified Tyrode-HEPES buffer without PGE_1 and indomethacin, and platelets were activated with 1U/ml thrombin from human plasma (Sigma-Aldrich), followed by incubation for 5 min at 37°C under constant shaking. After 5 min, ice-cold protease inhibitor (cOmplete Mini, EDTA-free, Roche) was added.

Subsequent centrifugation for 5 min at 1000 x *g* and 1 h at 20000 x *g*, both at 4°C, were performed to isolate the platelet releasate. Samples were concentrated using an Amicon 3K filter device (Merck Millipore) according to manufacturer's instructions. Protein concentration was measured using the CBQCA Protein Quantitation Kit (Molecular Probes) according to manufacturer's instructions. Proteins were separated and digested as described above. Injection volumes for each sample were further adjusted based on quantitative densitometry of the silver-stained gel. Mass spectrometry analysis of the peptides was performed as described above, with the following differences: raw files were searched against UniProt/SwissProt mouse database (2015_02); only oxidation of methionine was set as a variable modification; peptide identifications were accepted if they could be established at a probability > 85% and protein identifications were accepted if they could be established at a probability > 99.9% with at least two unique peptides.

Platelet isolation and lysis

Blood was collected into ACD by cardiac puncture, followed by a 1:1 dilution with filtered Tyrode-HEPES buffer. PRP was obtained by centrifugation at 100 x *g* for 8 min without brake. The top two-third of supernatant was transferred and supplemented with 1 µl/mg PGE_1, immediately followed by centrifugation at 800 x *g* for 10 min without brake. Supernatant PPP was transferred and the platelet pellet was washed twice by re-suspending in modified Tyrode-HEPES buffer supplemented with 1 µl/mg PGE_1, each time followed by centrifugation at 800 x *g* for 10 min without brake. Platelets were then lysed by a freeze-thaw and by subsequent lysis in ice-cold cell lysis buffer (Cell Signaling) supplemented with protease inhibitor (cOmplete Mini, EDTA-free, Roche) and

phosphatase inhibitor (PhosSTOP, Roche). Protein concentration was quantified using a BCA Protein Assay kit (Pierce, Thermo Scientific) according to manufacturer's instructions.

Murine bone marrow cell analysis

Bone marrow cells were isolated from femora using a syringe pre-filled with sterile PBS after cutting the epiphysis at either end of the bone. Cells were flushed out of the bone onto a 100 µm cell strainer (Corning), followed by centrifugation for 5 min at 350 x *g* at 4ºC. The supernatant was discarded and samples were resuspended in sterile PBS, followed by centrifugation at similar conditions. Supernatant was again removed and cells were resuspended in red cell lysis buffer (0.31 M ammonium chloride supplemented with sodium bicarbonate and EDTA) and incubated at 37ºC for 3 min, followed by adding 5x volume of PBS supplemented with 2% FBS and 0.1% sodium azide. Samples were then centrifuged for 5 min at 350 x *g* at 4ºC, followed by discarding of the supernatant and resuspension and lysis in 700 µl QIAzol reagent for RNA isolation.

Murine bone marrow immunohistochemistry

Femora were cleaned and placed in 4% formaldehyde and stored at 4°C for three days. Bones were then washed in PBS twice and decalcified in 0.38M EDTA in H_2O, pH 7.0 at 4°C for three weeks. Bone tissues were then dehydrated through a series of graded ethanol baths and embedded in paraffin for subsequent microtome sectioning (5 µm). After placing on glass slides, sections were baked at 60°C for 2 h. Epitope unmasking was achieved using hot sodium citrate buffer incubation for 20 min. Sections were washed

three times with PBS-T and incubated with primary antibodies (**Supplemental Table 2**) or species-matched isotopes overnight at 4°C after blocking with 10% FBS in PBS-T for 1 h. Following three 5-minute washes in PBS-T, sections were incubated for 1 h at room temperature with secondary antibody (**Supplemental Table 2**) in 10% FBS/PBS-T, according to the source of the primary antibody. Nuclei were stained with DAPI (1:1000 dilution) for 10 min. Sections were then mounted on a Vecta Mount (Vector Laboratories, cat. no. H-5000). Sections were visualized with a 20X objective using an inverted Nikon NI-E microscope equipped with a Yokogawa CSU-X1 spinning disk confocal unit and an Andor iXon 3 EM-CCD camera. Images were acquired using NIS-elements 4.0 software. Megakaryocyte counting was performed after blinding of immunostained sections using Fiji ImageJ software, version 2.0.0-rc-65/1.51w.

Luciferase reporter assays

The 3'-untranslated region of the mouse Was gene was cloned into the XhoI and PmeI linkers of the dual-luciferase reporter vector psiCHECK-2 (Promega) as described previously (9). The following primer set was used:

WAS F: AGTCATCTTCCTTCCAGCAA;

WAS R: TCTCTGTATAGCCCTGGCTG.

The reporter constructs (200 ng) were transfected together with miR-21 mimic (5 nmol/L) or mimic control in quadruplicate into HEK293T cells, previously plated (post 12 h) in 12-well plates using Lipofectamine RNAiMAX (Invitrogen) as above. After 48 h, cells were harvested in 200 µL Glo Lysis Buffer (Promega) and the activities of both Renilla and firefly were measured. Each lysate (20 µl) was analyzed using Dual-Glo Luciferase

reagents (Promega). Renilla luciferase activity was normalized to constitutive firefly luciferase activity for each well. Three independent experiments were performed.

Transfections in hPSC forward programming-derived megakaryocytes

In vitro production of megakaryocytes from hPSCs was carried out using forward programming (FoP) as described previously (10). Both hPSC lines were obtained from the Cambridge Biomedical Research Centre iPSC Core Facility. In short, hPSCs were transduced with replication-deficient lentiviral vectors to overexpress the 3 transcription factors FLI1, TAL1 and GATA1. After 2 days in a mesoderm promoting medium (Essential-6, Gibco) containing bone morphogenetic protein 4 (BMP4, 10ng/mL, Bio-Techne) and fibroblast growth factor 2 (FGF2, 20ng/mL, Bio-Techne) the cells were cultured in a megakaryocyte culture medium (CellGro, CellGenix) containing thrombopoietin (TPO, 20ng/mL, Bio-Techne) and stem cell factor (SCF, 25ng/mL, Gibco). The medium was refreshed by 50% exchange every 72 hours. Forward-programmed megakaryocytes (FoP-MK) were used after 20 or more days in culture when the population of megakaryocytes (as indicated by surface expression of CD41) was >90%. Before plating, cells were centrifuged at 120g and resuspended in megakaryocyte medium. Live cells were counted using a hemocytometer. FoP-MK were seeded at $3x10^5$ cells/ml in 6-well plates and combined with the transfection mix, consisting of TurboFect reagent (ThermoFisher Scientific) and reconstituted oligonucleotides prepared in RPMI medium following the manufacturer's instructions. A final concentration of 25 nM was used for both control and LNA-21 (PowerLNA, Qiagen).

References

1. Naba A, et al. The extracellular matrix: tools and insights for the “omics” era. *Matrix Biol* 2016;49:10–24.

2. Abonnenc M, et al. Extracellular matrix secretion by cardiac fibroblasts: role of microRNA-29b and microRNA-30c. *Circ Res* 2013;113(10):1138–1147.

3. Keller A, Nesvizhskii AI, Kolker E, Aebersold R. Empirical statistical model to estimate the accuracy of peptide identifications made by MS/MS and database search. *Anal Chem* 2002;74(20):5383–5392.

4. Nesvizhskii AI, Keller A, Kolker E, Aebersold R. A statistical model for identifying proteins by tandem mass spectrometry. *Anal Chem* 2003;75(17):4646–4658.

5. Kaudewitz D, et al. Association of microRNAs and yRNAs with platelet function. *Circ Res* 2016;118(3):420–432.

6. Xie F, Xiao P, Chen D, Xu L, Zhang B. miRDeepFinder: a miRNA analysis tool for deep sequencing of plant small RNAs. *Plant Mol Biol* 2012;80(1):75–84.

7. Woulfe D, et al. Defects in secretion, aggregation, and thrombus formation in platelets from mice lacking Akt2. *J Clin Invest* 2004;113(3):441–450.

8. Armstrong PCJ, et al. Novel whole blood assay for phenotyping platelet reactivity in mice identifies ICAM-1 as a mediator of platelet-monocyte interaction. *Blood* 2015;126(10):e11–e18.

9. Zampetaki A, et al. Role of miR-195 in aortic aneurysmal disease. *Circ Res* 2014;115(10):857–866.

10. Moreau T, et al. Large-scale production of megakaryocytes from human pluripotent stem cells by chemically defined forward programming. *Nat Commun* 2016;7:11208.

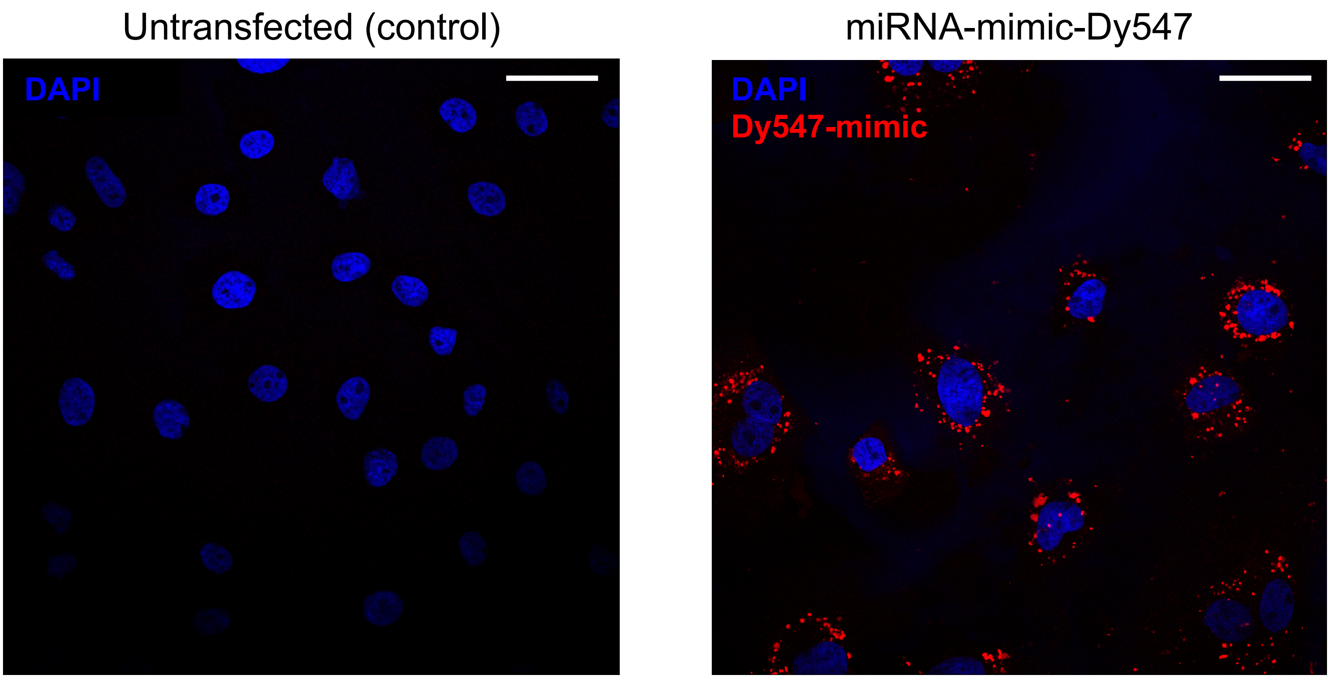

Supplemental Figure 1. Transfection of cardiac fibroblasts. Isolated murine cardiac fibroblasts were transfected with a microRNA labelled with red fluorescent dye (mimic-Dy547) to confirm transfection efficiency by microscopy. Nuclei were visualized by DAPI staining. Transfected cells displayed signal around the nuclei, indicating intracytoplasmatic localization. Shown images are representative of the n=2 for this transfection. Scale bar denotes 50 µm.

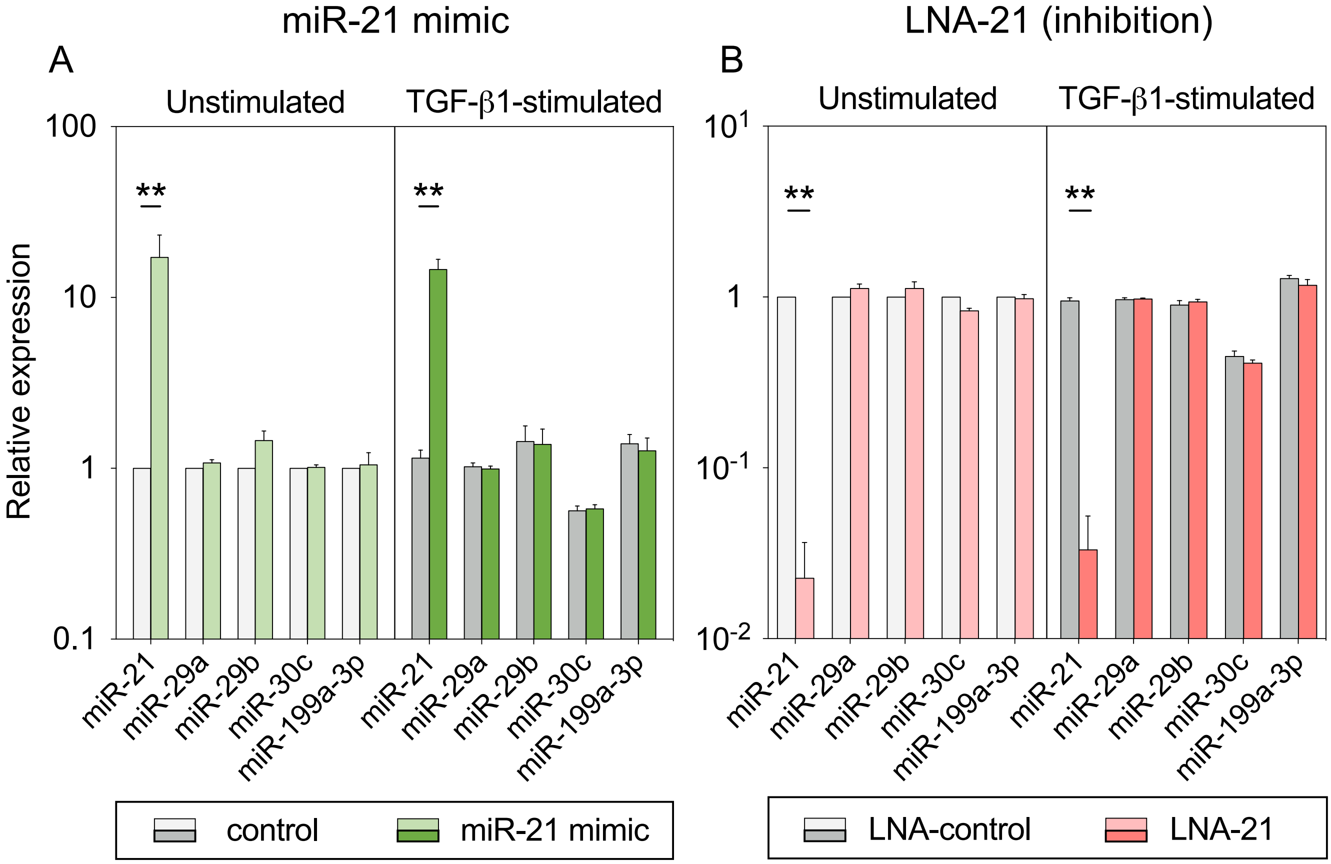

Supplemental Figure 2. MicroRNA levels after cardiac fibroblast transfections. RNA was isolated from cardiac fibroblasts, transfected with miR-21 mimic or control (**A**) and LNA-21 or LNA-control (**B**), 48 hours after TGF-β1 treatment. MicroRNA levels were determined by qPCR, using sno202 as a reference gene. Statistical analysis was performed using Wilcoxon's matched-pairs signed ranks test, n=4 for each condition. ** p<0.01. n=4 for each condition.

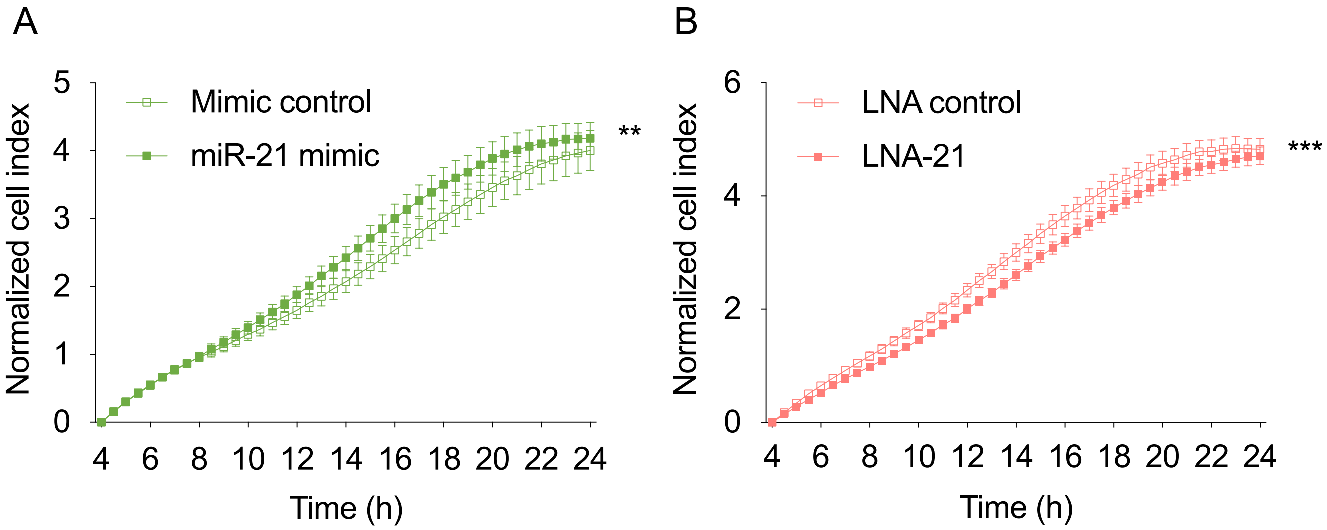

Supplemental Figure 3. Cardiac fibroblast proliferation after transfections. Primary murine cardiac fibroblasts were transfected with miR-21 mimic or inhibitor (LNA-21) or their respective controls and plated in an impedance-based proliferation assay plate (xCELLigence). This method records a cell index value that is considered a readout of cell number, size and attachment. Normalized cell index readings over time, recorded each 30 minutes, showed significantly increased and reduced proliferation with miR-21 mimic (**A**) and inhibitor (**B**) transfection, respectively (Friedman test with post-hoc Dunn's multiple comparisons test). Four biological replicates were analyzed for each condition. ** $p<0.01$, *** $p<0.001$. n=4 for each condition.

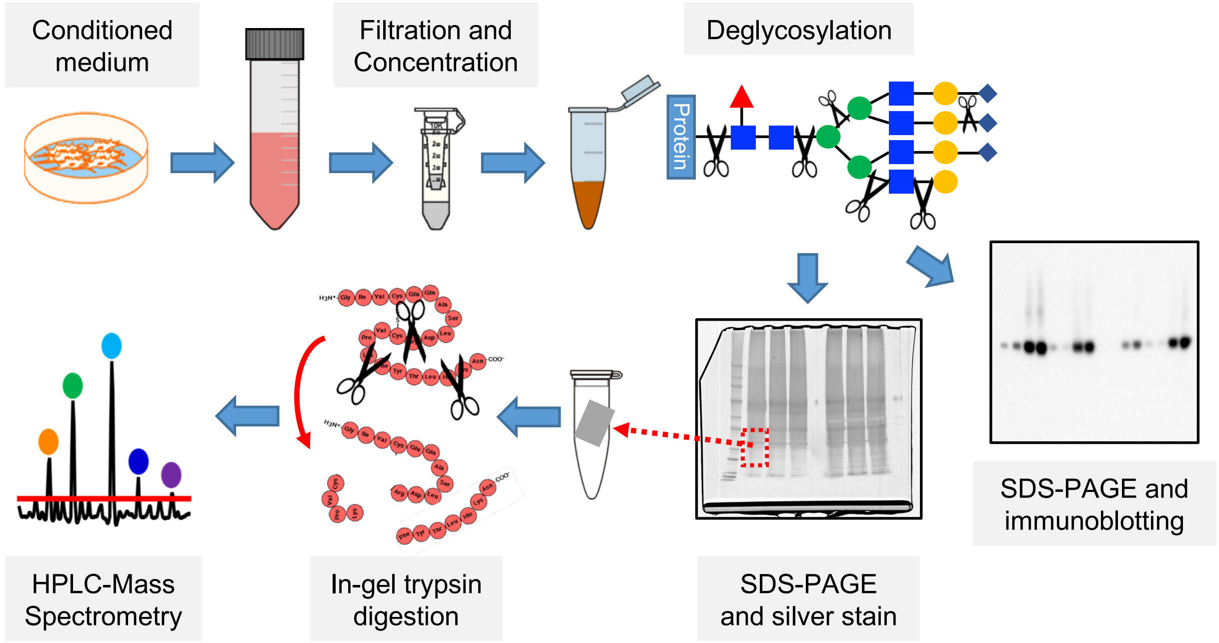

Supplemental Figure 4. Workflow for the analysis of fibroblast conditioned media. Conditioned media were collected from cardiac fibroblasts after transfections and subsequent TGF-β1 or control treatment. After removing cell debris by centrifugation, samples were filtered and concentrated using 3 kDa cutoff filter columns. Proteins were then deglycosylated by sequential incubation with deglycosylating enzymes. Samples were then reduced and denatured, following separation by polyacrylamide gel electrophoresis (SDS-PAGE). For immunoblotting, proteins were transferred onto nitrocellulose membranes. For mass spectrometry analysis, proteins in the gel were visualized by silver staining to support subsequent gel cutting. Each lane was cut into 12 gel bands without any gap in between. Proteins inside the gel pieces were digested using trypsin, followed by injection into an HPLC-coupled mass spectrometer for identification and quantification.

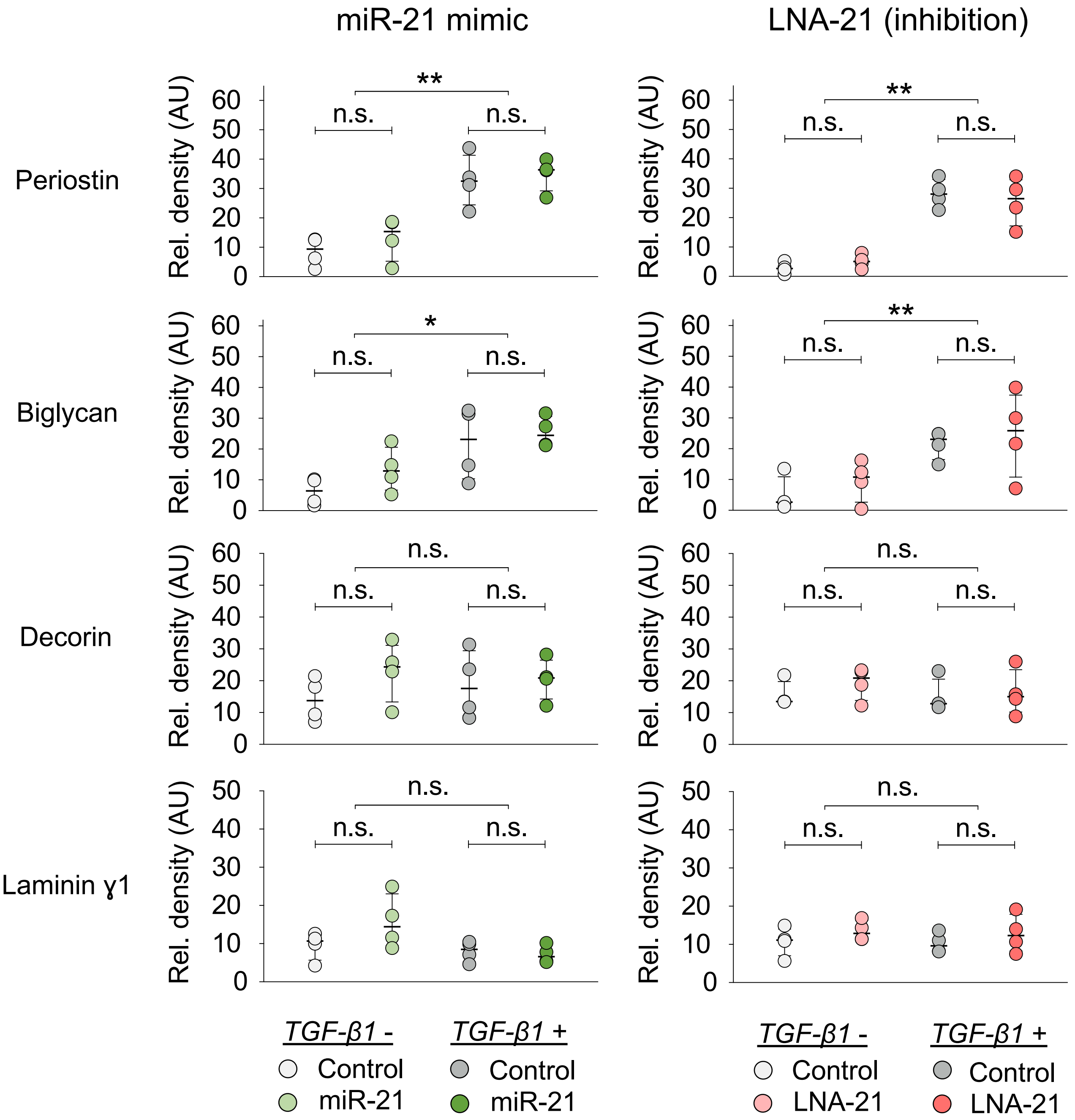

Supplemental Figure 5. Densitometry of cardiac fibroblast secretome immunoblots. Relative differences in optical density were determined in the immunoblot analysis of the fibroblast secretome. While a marked effect of TGF-β1 treatment was seen for periostin and biglycan, no significant differences were found between miR-21/LNA-21 and their respective controls. A paired statistical analysis was performed using the Wilcoxon matched-pairs signed ranks test, n=4 for each condition. TGF-β1 + / - indicates treatment 48 hours prior to conditioned media collection. AU, artificial units; n.s., not significant; * $p<0.05$, ** $p<0.01$.

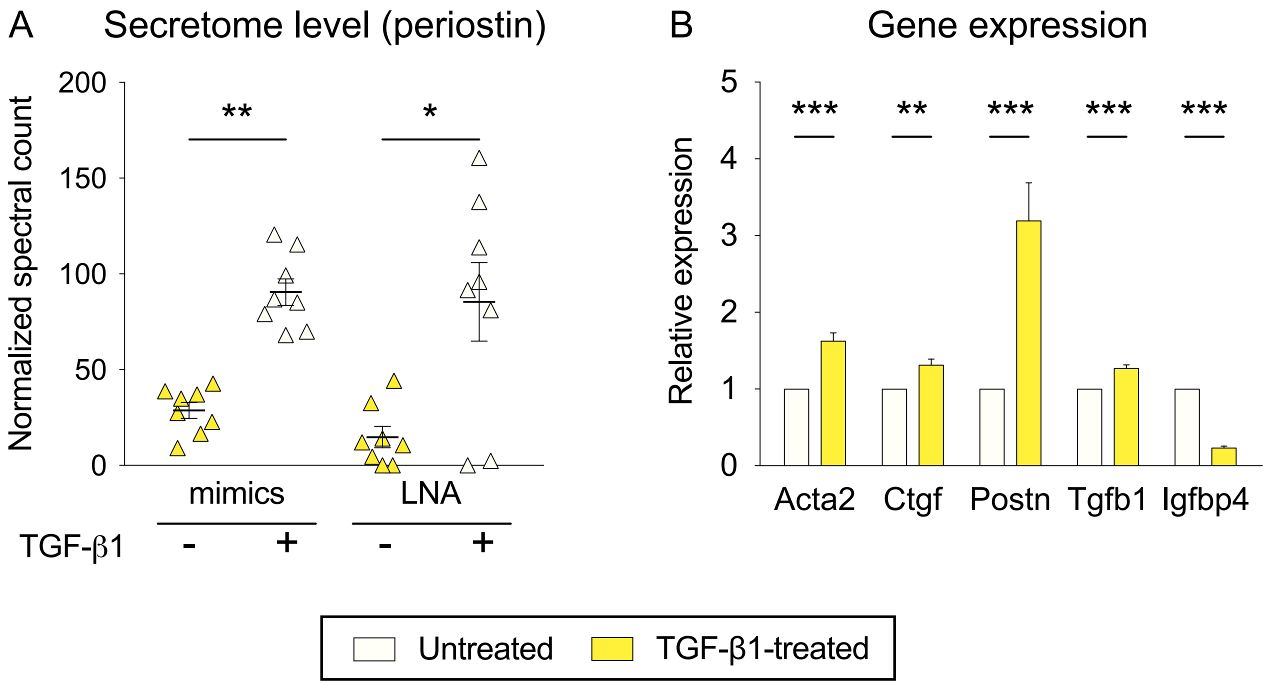

Supplemental Figure 6. Effects of TGF-β1 treatment. A: In transfected cardiac fibroblasts, mass spectrometric analysis for periostin in the secretome revealed markedly higher levels after TGF-β1 treatment. A paired statistical analysis was performed using the Wilcoxon matched-pairs signed ranks test, n=8 for each condition. **B:** Gene expression levels for several markers of TGF-β signaling and a myofibroblast-like phenotype were significantly induced by TGF-β1 treatment in transfected cardiac fibroblasts. *Ppia* was used as reference gene transcript. A paired t-test was used for statistical analysis, n=16 for each condition. * $p<0.05$, ** $p<0.01$, *** $p<0.001$.

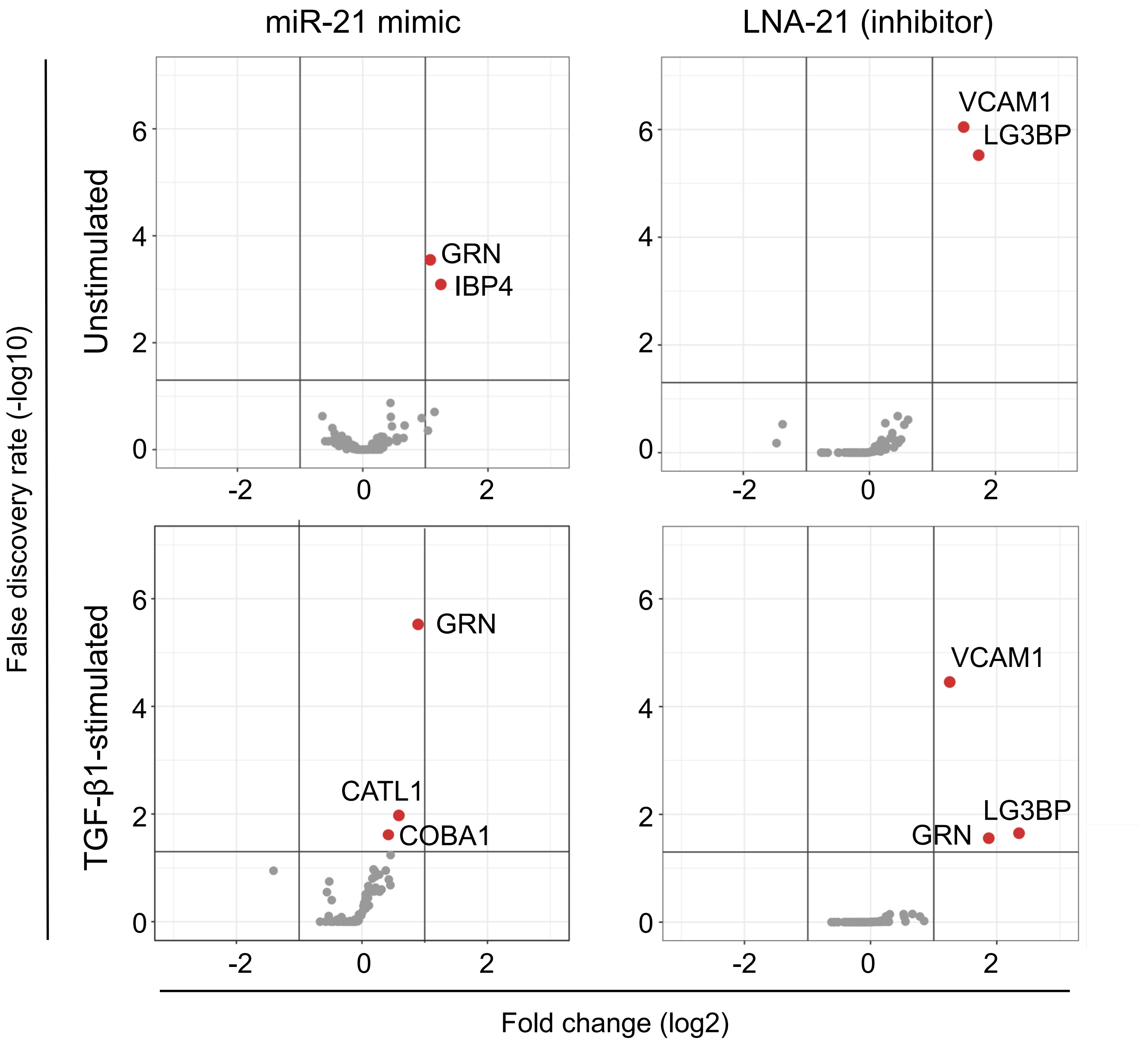

Supplemental Figure 7. Differential regulation of proteins in the secretome of transfected cardiac fibroblasts. Murine cardiac fibroblasts were transfected with miR-21 mimic or LNA-21 (inhibitor), followed by stimulation with TGF-β1 or control treatment, followed by HPLC-MS/MS analysis of their secretome. Volcano plots show fold changes, representing the ratios after miR-21 overexpression or inhibition compared with a corresponding control transfection; false discovery rate (FDR) is calculated using an empirical Bayes method and an FDR< 0.05 is considered significant. n=4 for each condition. CATL1, cathepsin L; COBA1, α1 chain of collagen XI; GRN, granulin; IBP4, insulin-like growth factor-binding protein 4; LG3BP, galectin-3-binding protein; VCAM1, vascular cell adhesion protein-1.

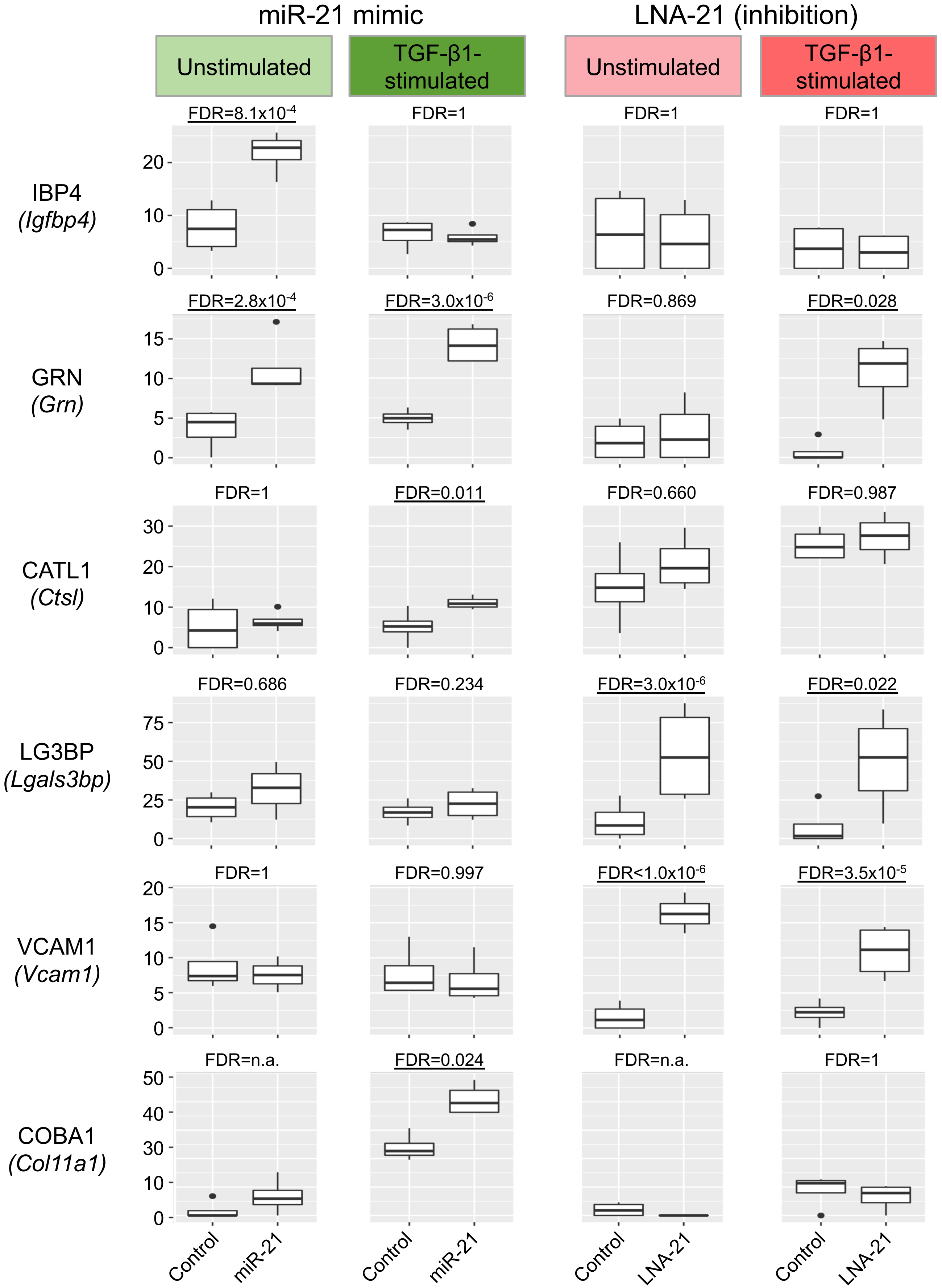

Supplemental Figure 8. Differentially expressed proteins in cardiac fibroblast secretome after miR-21 overexpression or inhibition. Box plots for proteins that were identified as differentially expressed in at least one of the studied conditions. The Y-axis represents the normalized spectral count. False discovery rate (FDR) <0.05 was considered statistically significant. n=4 for each condition. Gene IDs are listed in italic below the protein abbreviation to support comparison with **Supplemental Figure 10**. n.a., not applicable.

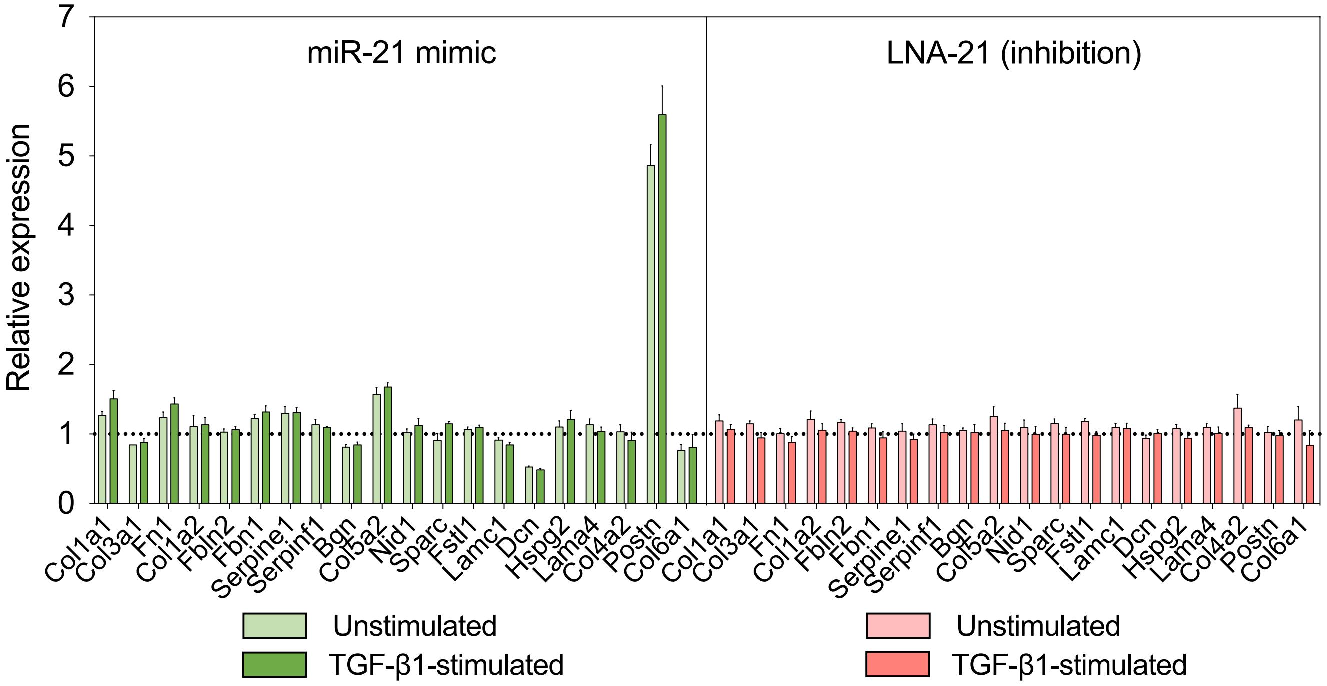

Supplemental Figure 9. Gene expression after miR-21 transfections in cardiac fibroblasts. Gene expression levels in cardiac fibroblasts were determined by qPCR to evaluate effects of miR-21 overexpression or inhibition. Genes encoding the twenty most abundantly detected proteins in the secretome were assessed. *Ppia* was used as reference gene transcript, with expression shown relative to the corresponding control transfection sample. Statistical analysis was performed using the Wilcoxon matched-pairs signed ranks test, n=4 for each condition.

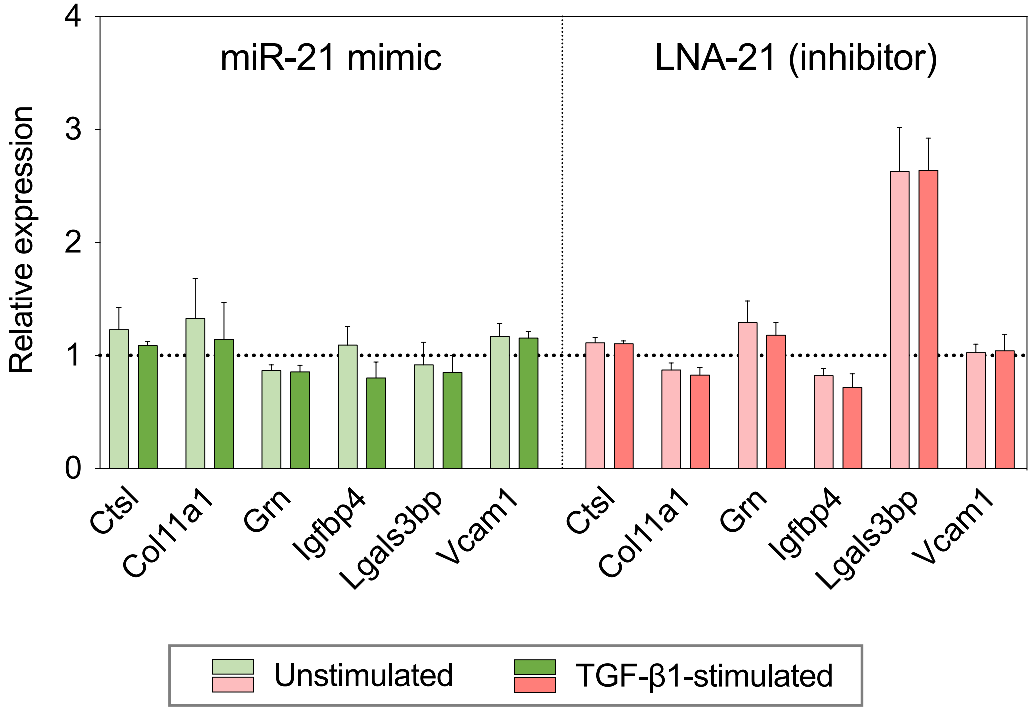

Supplemental Figure 10. Gene expression validation in transfected cardiac fibroblasts. Gene expression levels were determined by qPCR analysis in transfected fibroblasts for transcripts encoding significantly changing proteins in the secretome analysis. *Ppia* was used as a reference transcript, with expression shown relative to the corresponding control transfection sample. Statistical analysis was performed using the Wilcoxon matched-pairs signed ranks test, n=4 for each condition.

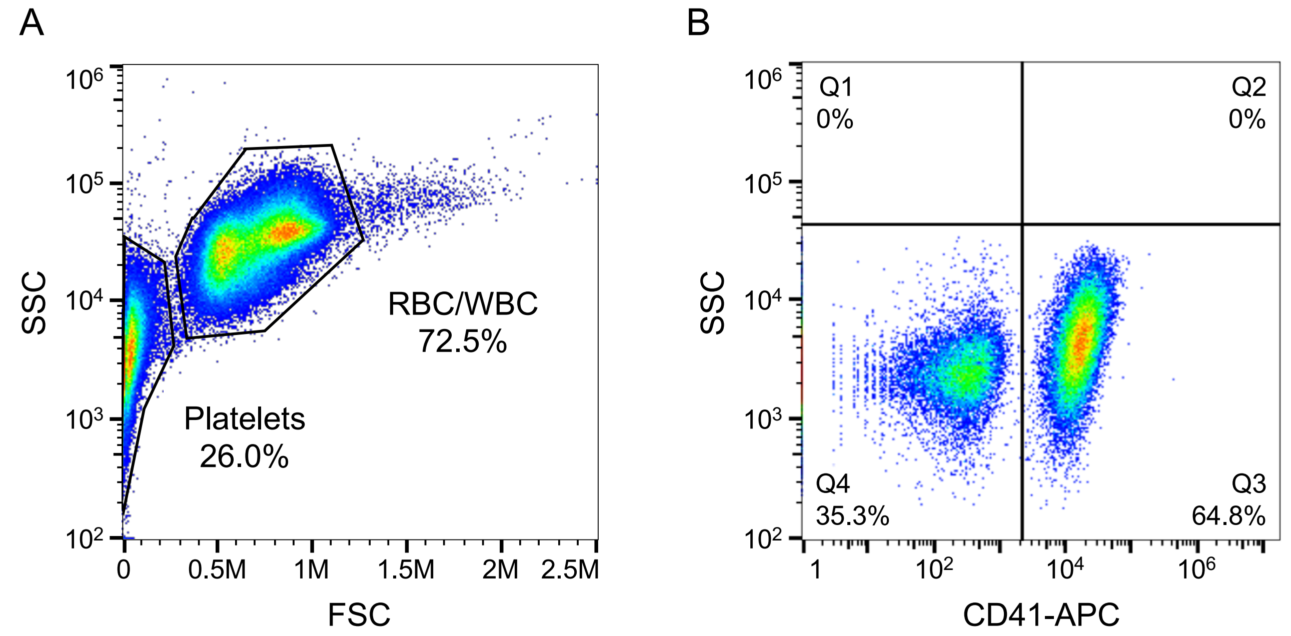

Supplemental Figure 11. Platelet count after pharmacological miR-21 inhibition. Blood was taken from mice after systemic treatment with antagomiR-21 or antagomiR-control (n=5 vs. 5). Platelets were stained by incubation with an APC-conjugated anti-CD41 antibody (CD41-APC). **A:** A gating window was selected for platelets and for red and white blood cells (RBC/WBC) based on forward (FSC) and side scatter (SSC). Median percentages across all samples are shown to indicate the proportion of gated events in relation to the total events. **B:** Gated platelets were analyzed for CD41-APC intensity, with quadrant 3 and 4 (Q3 and Q4) corresponding to stained and unstained platelets, respectively. Median percentages relating to the total gated platelet population are shown in each quadrant.

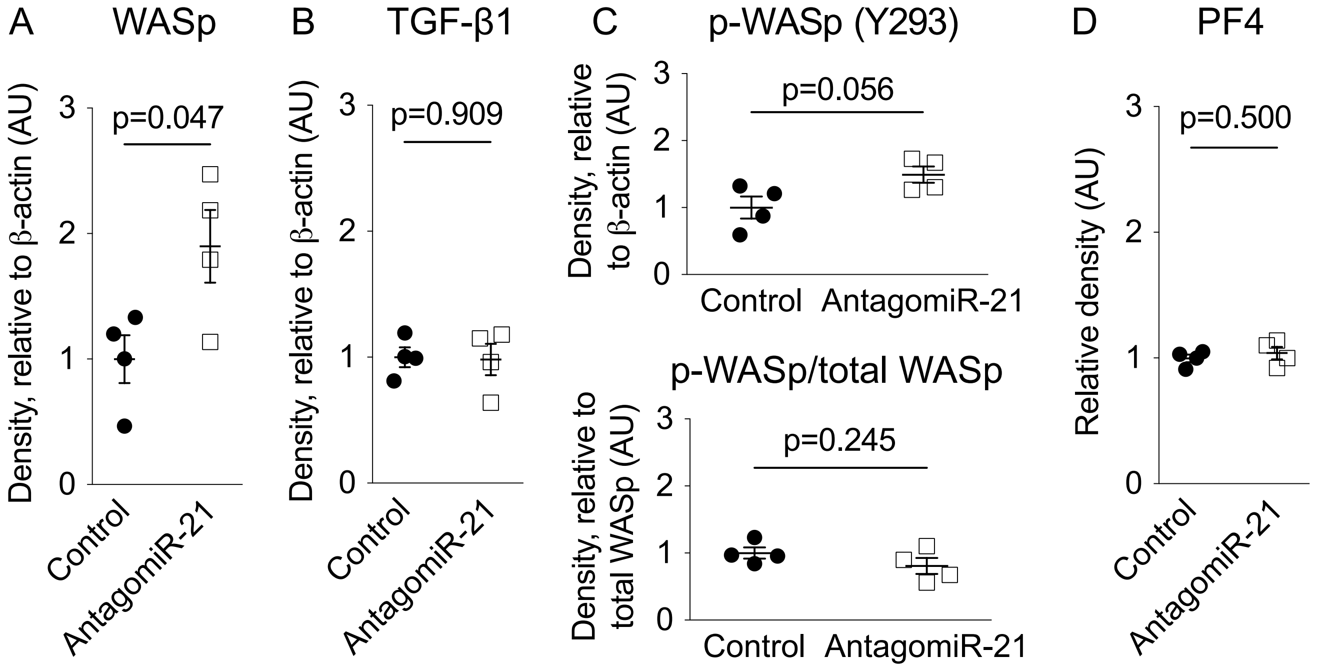

Supplemental Figure 12. Densitometry analysis for immunoblotting of lysed platelets isolated after pharmacological miR-21 inhibition. Immunoblotting was performed to determine levels of WASp (**A**), TGF-β1 (**B**), WASp phosphorylated at Tyrosine 293 (Y293; p-WASp; **C**) and PF4 (**D**). Densitometric analysis showed significantly higher levels of WASp in platelets from antagomiR-21-treated mice, whilst no difference was found for TGF-β1 or PF4. Signal intensity for WASp and TGF-β1 was normalized to the signal of β-actin. Intensity of p-WASp was normalized to either β-actin (**C**, top panel) or to total WASp intensity (**C**, bottom panel). Statistical testing was performed using Welch's t test. AU, arbitrary units. n=4 for each condition.

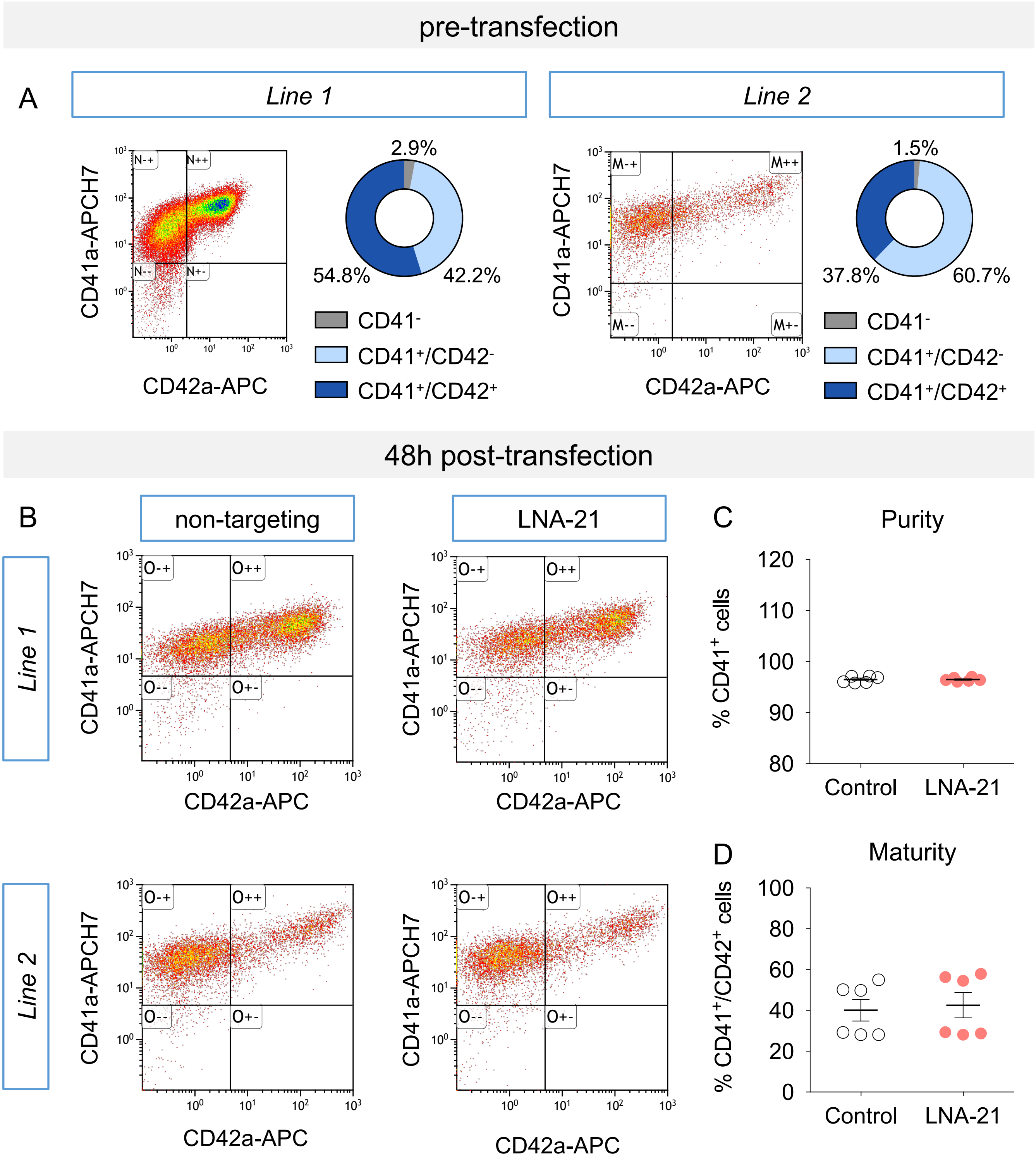

Supplemental Figure 13. Effect of miR-21 inhibition on megakaryocyte maturity. Megakaryocytes (MK) were produced using forward programming (FoP) of hPSCs as previously described (10). Two independent FoP-MK lines were transfected with a non-targeting LNA oligonucleotide (control) or miR-21 inhibitor (LNA-21), conjugated to a cationic polymer (TurboFect, ThermoFisher) to enhance transfection efficiency. **A:** Flow cytometry showing MK purity (as indicated by CD41 positivity) of >95%, of which >50% and >35% were mature MK (as indicated by CD42a positivity) in the first and second line, respectively. **B:** No significant difference in purity or maturity was seen between cells transfected with control or LNA-21 in either cell line. Transfections performed in triplicate for each condition. Representative flow cytometry plots are provided, with individual data for purity and maturity shown in **C** and **D**, respectively. Statistical comparison was performed with a paired t-test.

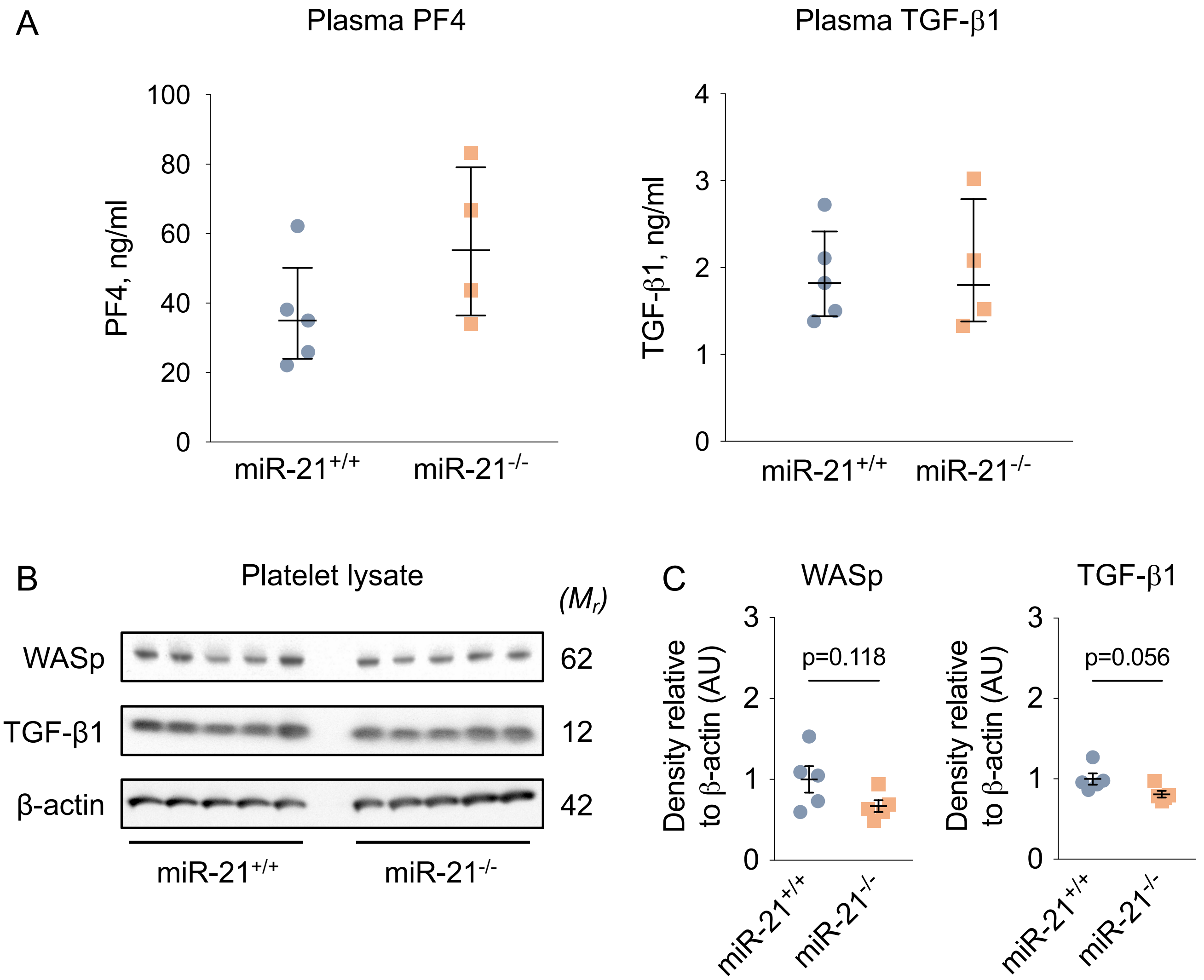

Supplemental Figure 14. Levels of WASp, PF4 and TGF-β1 in miR-21 null mice. Blood was taken from miR-21 null mice and wildtype littermates into ACD buffer, followed by preparation of platelet-rich plasma (PRP) and platelet-poor plasma (PPP) by centrifugation. **A:** Levels of PF4 and TGF-β1 in PPP were determined by ELISA (n=5 vs. 4), showing no significant difference for either protein. **B:** Platelets were isolated from miR-21 null mice and washed, followed by lysis and subsequent immunoblotting. Immunoblots showed unaltered levels of Wiskott-Aldrich Syndrome protein (WASp) and TGF-β1 content. **C:** Corresponding densitometry analysis of immunoblots, showing no significant differences. Signal intensity for TGF-β1 and WASp was normalized to the signal of β-actin. Statistical testing was performed using Welch's t test. AU, arbitrary units. n=5 for each condition.

Supplemental Table 1. TaqMan hydrolysis assays for use in qPCR.

Gene target	Corresponding protein ID	Assay ID
Acta2	ACTA_MOUSE	Mm01546133_m1
Actb	ACTB_MOUSE	Mm02619580_g1
Bgn	PGS1_MOUSE	Mm01191753_m1
Col11a1	COBA1_MOUSE	Mm00483387_m1
Col1a1	CO1A1_MOUSE	Mm00801666_g1
Col1a2	CO1A2_MOUSE	Mm00483888_m1
Col3a1	CO3A1_MOUSE	Mm00802300_m1
Col4a2	CO4A2_MOUSE	Mm00802386_m1
Col5a2	CO5A2_MOUSE	Mm00483675_m1
Col6a1	CO6A1_MOUSE	Mm00487160_m1
Ctgf	CTGF_MOUSE	Mm01192933_g1
Ctsl	CATL1_MOUSE	Mm00515597_m1
Dcn	PGS2_MOUSE	Mm00514535_m1
Fbln2	FBLN2_MOUSE	Mm00484266_m1
Fbn1	FBN1_MOUSE	Mm00514908_m1
Fn1	FINC_MOUSE	Mm01256744_m1
Fstl1	FSTL1_MOUSE	Mm00433371_m1
Gapdh	G3P_MOUSE	Mm99999915_g1
Grn	GRN_MOUSE	Mm00433848_m1
Hspg2	PGBM_MOUSE	Mm01181173_g1
Igfbp4	IBP4_MOUSE	Mm00494922_m1
Itga2b	ITA2B_MOUSE	Mm00439741_m1
Lama4	LAMA4_MOUSE	Mm01193660_m1
Lamc1	LAMC1_MOUSE	Mm00711820_m1
Lgals3bp	LG3BP_MOUSE	Mm00478303_m1
Ltbp1	LTBP1_MOUSE	Mm00498234_m1
Nid1	NID1_MOUSE	Mm00477827_m1
Pf4	PLF4_MOUSE	Mm00451315_g1
Postn	POSTN_MOUSE	Mm01284919_m1
Ppbp	CXCL7_MOUSE	Mm00470163_m1
Ppia	PPIA_MOUSE	Mm02342430_g1
Ptprc	PTPRC_MOUSE	Mm01293577_m1
Serpine1	PAI1_MOUSE	Mm00435858_m1
Serpinf1	PEDF_MOUSE	Mm00441270_m1
Sparc	SPRC_MOUSE	Mm00486332_m1
Tgfb1	TGFB1_MOUSE	Mm01178820_m1
Tgfbr2	TGFR2_MOUSE	Mm03024091_m1
Vcam1	VCAM1_MOUSE	Mm01320970_m1
Was	WASP_MOUSE	Mm00494167_m1

miRNA target	Assay ID
cel-miR-39-3p	000200
U6	001973
RNU48	001006
miR-1	002222
miR-21	000397
miR-29a	002112
miR-29b	000413
miR-30c	000419
miR-31	002279
miR-126	002228
miR-133	002246
miR-143	002249
miR-145	002278
miR-146a	000468
miR-199a-3p	002304
miR-499	001352

Supplemental Table 2. Details of used antibodies.

**Target protein**	**Host species**	**Application**	**Company, catalogue number**
Laminin-ɣ1	Rat	Immunoblotting (1:500)	Abcam, ab17792
Periostin	Rabbit	Immunoblotting (1:1000)	Novus Biologicals, NBP1-30042
Biglycan	Rabbit	Immunoblotting (1:500)	Abcam, ab94460
Decorin	Rabbit	Immunoblotting (1:130)	Santa Cruz, sc-22753
Argonaute2	Mouse	Immunoprecipitation	Abcam, ab57113
Anti-CD41	Rat	Flow cytometry, APC-labelled (1:10 to whole blood)	BioLegend, 133913
Anti-CD42b	Rat	Immunodepletion (4 mg/kg)	Emfret Analytics, R300
PF4	Rat	Immunohistochemistry; immunoblotting (1:1000)	R&D Systems, MAB595
WASp	Rabbit	Immunoblotting (1:1000)	Cell Signaling, 4860
p-WASp	Rabbit	Immunoblotting (1:500)	Abcam, ab59278
TGF-β1	Rabbit	Immunoblotting (1:1000); immunohistochemistry	Abcam, ab179695
β-actin	Mouse	Immunoblotting (1:10000)	Sigma, A1978
HRP-anti-rabbit	Mouse	Immunoblotting (secondary antibody, 1:5000)	Jackson ImmunoResearch, 211032171
HRP-anti-mouse	Goat	Immunoblotting (secondary antibody, 1:5000)	Jackson ImmunoResearch, 115035174
HRP-anti-rat	Goat	Immunoblotting (secondary antibody, 1:2000)	Jackson ImmunoResearch, 112055175

Supplemental Table 3. ECM proteins identified in unstimulated or TGF-β1-stimulated murine CF secretome following miR-21 mimic or inhibitor (LNA-21) transfection. Secretome was analysed by gel LC-MS/MS analysis using an LTQ-Orbitrap XL. Values shown are mean±SEM of normalized spectral counts, based on four biological replicates for each condition. Differential expression was determined using a hierarchical Bayes estimation of generalized linear mixed effects model. FDR is calculated using an empirical Bayes method, with an FDR<0.05 considered significant. Proteins that yielded an FDR<0.05 for at least one condition are marked in bold.

				miR-21 mimic - Unstimulated cardiac fibroblasts				miR-21 mimic - TGF-β1-stimulated cardiac fibroblasts				miR-21 inhibitor (LNA) - Unstimulated cardiac fibroblasts				miR-21 inhibitor (LNA) - TGF-β1-stimulated cardiac fibroblasts			
				Mean ± SEM				Mean ± SEM				Mean ± SEM				Mean ± SEM			
Full protein name	Accession ID	Gene	MW (kDa)	Control	miR-21	FDR	log2 FC	Control	miR-21	FDR	log2 FC	Control	miR-21	FDR	log2 FC	Control	miR-21	FDR	log2 FC
Adipocyte enhancer-binding protein 1	AEBP1	Aebp1	128	10.3±1.4	8.0±2.5	0.898	-0.24	18.7±2.4	15.2±1.8	1.000	-0.24	6.5±1.4	4.9±3.4	0.993	-0.33	6.5±2.3	9.6±3.5	0.984	0.18
Agrin	AGRIN	Agrn	208	0.4±0.5	4.4±1.5	0.198	1.15	0.0±0.0	4.3±0.8	n.a.	n.a.	0.0±0.0	3.5±1.5	n.a.	n.a.	0.0±0.0	9.3±4.5	n.a.	n.a.
Annexin A2	ANXA2	Anxa2	39	6.5±2.5	3.6±1.2	0.623	-0.45	6.8±2.2	4.9±2.2	0.993	-0.28	3.1±2.2	2.8±2.8	n.a.	n.a.	2.9±1.8	6.2±3.5	0.974	0.55
Apolipoprotein E	APOE	Apoe	36	4.7±1.8	6.2±2.4	1.000	0.24	2.0±1.2	3.2±1.9	0.274	0.19	11.6±4.4	4.8±1.7	0.996	-0.76	0.6±0.6	1.6±1.0	n.a.	n.a.
A disintegrin and metalloproteinase with thrombospondin motifs 5	ATS5	Adamts5	102	10.6±2.6	12.7±3.2	0.997	0.28	1.5±0.9	1.4±0.5	0.734	-0.05	0.0±0.0	0.0±0.0	n.a.	n.a.	0.0±0.0	0.0±0.0	n.a.	n.a.
Bone morphogenetic protein 1	BMP1	Bmp1	112	15.2±3.7	26.0±6.8	0.623	0.58	19.7±2.4	27.1±3.4	0.145	0.21	1.8±1.8	2.6±2.6	n.a.	n.a.	19.1±12.1	18.9±9.1	0.994	0.03
Cathepsin B	CATB	Ctsb	37	14.2±1.6	16.9±1.4	0.776	0.15	11.6±0.9	10.8±1.4	0.996	-0.21	20.2±4.2	20.3±4.4	1.000	-0.16	21.6±4.4	24.3±4.2	0.981	0.17
Cathepsin D	CATD	Ctsd	45	19.6±3.8	22.2±4.8	1.000	0.08	30.1±5.1	22.9±6.1	1.000	-0.46	11.5±3.6	14.9±0.6	0.536	0.33	23.9±3.6	26.5±2.2	0.943	0.16
**Cathepsin L1**	**CATL1**	**Ctsl**	**38**	**5.2±3.1**	**6.5±1.3**	**1.000**	**0.18**	**5.2±2.1**	**11.1±0.8**	**0.011**	**0.59**	**14.8±4.6**	**20.8±3.4**	**0.660**	**0.46**	**25.4±2.0**	**27.4±2.8**	**0.987**	**0.09**
Cathepsin Z	CATZ	Ctsz	34	3.2±1.3	4.2±1.7	0.998	0.23	1.1±1.1	0.7±0.7	n.a.	n.a.	10.1±4.9	16.0±5.4	0.570	0.50	17.2±1.6	18.7±1.9	0.994	0.01
C-C motif chemokine 2	CCL2	Ccl2	16	2.6±1.0	0.8±0.5	0.700	-0.54	0.0±0.0	0.0±0.0	n.a.	n.a.	0.0±0.0	1.0±1.0	n.a.	n.a.	0.0±0.0	1.5±1.5	n.a.	n.a.
Collagen alpha-1(I) chain	CO1A1	Col1a1	138	728.9±67.5	821.2±87.5	0.998	0.01	643.2±42.3	782.7±17.9	0.763	-0.01	211.3±109.8	114.1±29.8	1.000	-0.15	227.1±55.7	184.8±39.2	0.979	0.13
Collagen alpha-2(I) chain	CO1A2	Col1a2	130	427.1±20.6	488.3±29.6	0.888	-0.12	404.1±10.3	433.7±16.1	0.312	0.06	85.5±22.7	77.1±5.8	0.933	0.04	110.6±27.5	89.7±18.3	1.000	-0.10
Collagen alpha-1(III) chain	CO3A1	Col3a1	139	407.3±34.4	489.7±53.2	1.000	0.07	318.8±21.1	359.1±13.0	0.311	0.07	335.3±130.3	250.7±63.7	1.000	-0.30	291.3±71.6	228.1±59.9	1.000	-0.12
Collagen alpha-1(IV) chain	CO4A1	Col4a1	161	52.0±2.0	47.1±4.2	0.814	-0.17	56.1±5.3	49.3±2.0	1.000	-0.19	24.2±7.7	9.2±6.0	0.297	-1.38	43.5±10.4	34.9±9.8	1.000	-0.36
Collagen alpha-2(IV) chain	CO4A2	Col4a2	167	86.1±3.6	87.0±12.3	0.998	0.02	99.4±6.0	83.4±9.0	0.995	-0.17	21.7±3.4	24.7±6.6	1.000	-0.17	27.0±7.2	26.2±6.6	0.996	-0.01
Collagen alpha-1(V) chain	CO5A1	Col5a1	184	31.3±2.7	28.6±1.3	0.653	-0.24	33.5±3.4	38.0±3.7	0.370	0.07	17.1±9.6	7.5±2.6	0.997	-0.50	29.6±9.2	18.3±8.5	1.000	-0.58
Collagen alpha-2(V) chain	CO5A2	Col5a2	145	149.7±8.4	159.4±7.5	1.000	0.00	176.0±13.0	194.3±13.5	0.343	0.07	33.6±25.4	12.8±8.3	1.000	-0.72	55.1±17.8	35.1±12.4	1.000	-0.39
Collagen alpha-1(VI) chain	CO6A1	Col6a1	108	45.0±1.9	57.7±5.6	0.610	0.23	36.2±4.4	30.8±3.2	0.998	-0.29	72.9±20.6	69.9±21.3	1.000	-0.24	66.2±19.0	56.9±17.6	1.000	-0.15

Supplemental Table 3. (continued)

				miR-21 mimic - Unstimulated cardiac fibroblasts				miR-21 mimic - TGF-β1-stimulated cardiac fibroblasts				miR-21 inhibitor (LNA) - Unstimulated cardiac fibroblasts				miR-21 inhibitor (LNA) - TGF-β1-stimulated cardiac fibroblasts			
				Mean ± SEM				Mean ± SEM				Mean ± SEM				Mean ± SEM			
**Full protein name**	**Accession ID**	**Gene**	**MW (kDa)**	**Control**	**miR-21**	**FDR**	**log2 FC**	**Control**	**miR-21**	**FDR**	**log2 FC**	**Control**	**miR-21**	**FDR**	**log2 FC**	**Control**	**miR-21**	**FDR**	**log2 FC**
Collagen alpha-2(VI) chain	CO6A2	Col6a2	110	14.8±0.5	13.0±0.9	0.856	-0.13	9.6±0.7	6.1±0.8	0.179	-0.52	21.5±7.9	19.6±7.4	0.992	-0.23	16.5±7.3	9.5±3.9	1.000	-0.25
Collagen alpha-1(VIII) chain	CO8A1	Col8a1	74	2.1±0.8	3.5±0.5	0.579	0.33	0.0±0.0	0.0±0.0	n.a.	n.a.	0.0±0.0	0.0±0.0	n.a.	n.a.	4.3±2.6	2.6±1.7	1.000	-0.42
**Collagen alpha-1(XI) chain**	**COBA1**	**Col11a1**	**181**	**1.7±1.7**	**6.8±3.2**	**n.a.**	**n.a.**	**23.9±2.4**	**40.8±2.8**	**0.024**	**0.44**	**2.1±1.2**	**0.0±0.0**	**n.a.**	**n.a.**	**8.9±3.0**	**6.5±2.4**	**1.000**	**-0.22**
Collagen alpha-1(XII) chain	COCA1	Col12a1	340	27.4±3.3	33.7±6.3	1.000	0.07	48.6±7.4	58.7±6.0	0.349	0.09	26.2±15.5	17.2±8.6	0.989	-0.02	79.6±32.2	53.3±23.5	1.000	-0.15
Collagen alpha-1(XIV) chain	COEA1	Col14a1	193	0.6±0.6	4.0±1.5	n.a.	n.a.	1.1±1.1	0.9±0.9	n.a.	n.a.	3.6±1.5	3.0±1.2	0.993	-0.27	5.1±2.5	3.0±1.8	1.000	-0.32
Collagen alpha-1(XV) chain	COFA1	Col15a1	140	2.3±1.3	2.1±1.4	1.000	-0.03	2.2±0.8	3.6±1.7	0.501	0.11	2.7±1.6	1.4±1.4	n.a.	n.a.	5.9±2.2	5.1±2.0	1.000	-0.09
Macrophage colony-stimulating factor 1	CSF1	Csf1	61	8.3±0.6	8.9±1.7	1.000	0.10	15.4±2.3	15.7±3.2	0.993	-0.13	4.6±0.6	7.1±2.6	0.877	0.11	11.3±4.2	11.3±4.8	1.000	-0.10
Versican core protein	CSPG2	Vcan	367	2.0±2.0	4.9±1.7	n.a.	n.a.	2.0±1.2	3.6±1.3	0.273	0.20	1.5±1.5	3.5±2.3	n.a.	n.a.	8.5±3.4	0.0±0.0	n.a.	n.a.
Connective tissue growth factor	CTGF	Ctgf	38	14.1±0.4	9.0±1.6	0.498	-0.45	30.9±2.7	25.9±1.7	0.825	-0.33	9.8±1.4	12.8±2.7	0.941	0.05	43.7±4.3	59.6±7.8	0.715	0.30
Collagen triple helix repeat-containing protein 1	CTHR1	Cthrc1	26	1.9±1.2	3.6±1.5	0.915	0.33	3.5±1.4	5.8±1.8	0.277	0.28	2.1±1.3	0.6±0.6	n.a.	n.a.	3.1±1.1	3.2±1.1	0.987	0.08
Cytochrome c, somatic	CYC	Cycs	12	4.1±0.9	3.4±1.4	0.998	0.02	5.1±0.7	3.6±0.6	0.982	-0.38	6.0±2.5	4.0±2.4	0.995	-0.34	1.8±1.9	1.8±1.8	n.a.	n.a.
Cystatin-C	CYTC	Cst3	16	12.3±4.3	18.5±3.0	0.793	0.24	17.0±2.4	21.2±1.8	0.107	0.18	12.5±6.2	9.7±5.8	0.996	-0.39	8.1±4.7	3.0±1.8	1.000	-0.62
Dystroglycan	DAG1	Dag1	97	4.0±0.7	2.5±1.1	0.926	-0.22	2.5±1.0	1.1±1.1	0.997	-0.50	4.5±1.6	3.0±1.8	0.999	-0.27	5.9±2.3	6.9±2.3	0.976	0.15
Dermatopontin	DERM	Dpt	24	12.1±3.0	16.9±2.9	0.731	0.41	12.3±1.4	11.5±1.0	1.000	-0.14	23.3±6.6	16.3±6.8	0.994	-0.37	7.7±3.1	5.0±2.3	1.000	-0.33
Extracellular matrix protein 1	ECM1	Ecm1	63	37.3±2.3	48.6±2.6	0.737	0.21	27.0±2.9	33.4±1.9	0.114	0.20	70.8±18.3	59.8±14.7	0.996	-0.21	55.1±13.7	56.0±13.2	0.996	-0.02
EMILIN-1	EMIL1	Emilin1	108	2.4±1.4	5.9±1.6	0.597	0.54	3.1±1.2	3.1±1.1	0.926	-0.10	3.6±1.6	4.6±2.7	0.991	-0.03	6.0±2.6	5.7±2.1	0.994	0.03
Fibulin-1	FBLN1	Fbln1	78	12.0±2.4	12.6±2.2	1.000	0.15	5.8±1.3	2.5±1.6	1.000	-0.67	23.3±9.8	29.5±11.2	0.870	0.12	10.5±4.3	4.7±1.9	1.000	-0.52
Fibulin-2	FBLN2	Fbln2	132	226.8±18.1	239.1±19.1	0.999	0.00	215.5±7.5	220.6±8.7	0.366	0.06	307.7±45.1	309.2±46.9	0.769	0.09	291.1±44.8	283.0±37.2	0.991	0.02
EGF-containing fibulin-like extracellular matrix protein 1	FBLN3	Efemp1	55	21.0±2.2	35.8±2.5	0.245	0.45	1.3±0.9	3.4±2.0	0.210	0.45	51.8±17.7	43.8±14.8	1.000	-0.29	11.1±5.7	8.8±3.8	0.996	0.01
EGF-containing fibulin-like extracellular matrix protein 2	FBLN4	Efemp2	49	22.3±3.2	28.2±0.4	0.805	0.23	15.9±1.7	15.1±4.9	0.995	-0.33	27.2±8.3	29.4±7.9	0.927	0.13	23.2±7.8	25.7±7.1	0.987	0.28
Fibulin-5	FBLN5	Fbln5	50	24.4±2.0	22.4±2.3	0.851	-0.16	35.4±3.4	29.4±6.0	1.000	-0.29	0.0±0.0	1.7±1.0	n.a.	n.a.	12.1±7.0	9.2±6.7	1.000	-0.23
Fibrillin-1	FBN1	Fbn1	312	57.2±6.8	71.7±9.3	0.571	0.29	44.2±5.5	48.7±3.6	0.524	0.03	292.4±88.2	262.6±75.4	1.000	-0.07	258.2±65.1	243.1±68.4	1.000	-0.11
Fibronectin	FINC	Fn1	273	330.0±15.4	323.2±26.4	1.000	0.04	401.7±18.6	379.0±30.1	1.000	-0.10	232.4±68.4	239.6±66.9	0.877	0.09	247.6±61.7	237.2±84.9	1.000	-0.04
Fibromodulin	FMOD	Fmod	43	14.0±2.7	10.6±4.1	0.903	-0.26	8.1±1.3	7.4±3.0	1.000	-0.37	0.9±0.9	0.0±0.0	n.a.	n.a.	0.0±0.0	0.0±0.0	n.a.	n.a.
Follistatin-related protein 1	FSTL1	Fstl1	35	84.0±8.0	69.6±5.5	0.993	-0.08	85.7±3.4	84.8±3.3	0.512	0.02	50.5±7.0	54.5±9.4	1.000	-0.08	88.1±16.8	82.8±17.3	1.000	-0.08
Gelsolin	GELS	Gsn	86	59.8±7.0	66.2±13.4	1.00	0.01	14.3±2.2	8.3±3.3	1.00	-0.50	87.0±29.8	75.9±26.3	1.00	-0.33	43.1±19.7	35.8±16.1	1.00	-0.19

Supplemental Table 3. (continued)

Full protein name	Accession ID	Gene	MW (kDa)	miR-21 mimic - Unstimulated cardiac fibroblasts, Mean ± SEM Control	miR-21	FDR	log2 FC	miR-21 mimic - TGF-β1-stimulated cardiac fibroblasts, Mean ± SEM Control	miR-21	FDR	log2 FC	miR-21 inhibitor (LNA) - Unstimulated cardiac fibroblasts, Mean ± SEM Control	miR-21	FDR	log2 FC	miR-21 inhibitor (LNA) - TGF-β1-stimulated cardiac fibroblasts, Mean ± SEM Control	miR-21	FDR	log2 FC
**Granulins**	**GRN**	**Grn**	**63**	**3.7±1.3**	**11.2±2.0**	**2.8E-04**	**1.08**	**4.9±0.6**	**14.2±1.3**	**3.0E-06**	**0.89**	**2.1±1.3**	**3.2±2.0**	**0.87**	**0.26**	**0.7±0.8**	**10.8±2.2**	**0.03**	**1.87**
Insulin-like growth factor-binding protein 2	IBP2	Igfbp2	33	3.4±1.6	0.6±0.7	n.a.	n.a.	9.0±1.9	10.1±1.5	0.51	0.07	0.0±0.0	0.0±0.0	n.a.	n.a.	1.9±2.0	1.2±1.2	n.a.	n.a.
**Insulin-like growth factor-binding protein 4**	**IBP4**	**Igfbp4**	**28**	**7.8±2.3**	**21.8±2.0**	**8.1E-04**	**1.25**	**6.5±1.4**	**5.9±0.9**	**1.00**	**-0.17**	**6.8±4.0**	**5.5±3.3**	**1.00**	**-0.19**	**3.8±2.2**	**3.1±1.8**	**1.00**	**-0.26**
Insulin-like growth factor-binding protein 7	IBP7	Igfbp7	29	49.6±5.8	53.8±7.3	1.00	0.02	65.8±5.8	54.3±5.1	1.00	-0.26	31.1±5.6	33.3±2.4	0.97	0.02	50.9±5.2	43.0±6.7	1.00	-0.14
Plasma protease C1 inhibitor	IC1	Serping1	56	13.3±2.0	10.6±3.7	0.84	-0.30	8.5±1.3	13.2±3.1	0.25	0.14	32.3±13.2	47.1±12.1	0.58	0.40	20.2±9.1	28.6±8.1	0.71	0.66
Laminin subunit alpha-2	LAMA2	Lama2	344	10.4±1.7	12.2±1.0	0.99	0.23	15.2±3.3	24.9±2.1	0.06	0.46	15.8±6.7	12.5±6.2	1.00	-0.25	46.8±18.5	39.6±15.8	1.00	-0.03
Laminin subunit alpha-4	LAMA4	Lama4	202	67.3±5.7	60.7±5.4	0.85	-0.14	49.4±5.1	44.4±1.8	0.99	-0.17	70.2±23.7	61.8±19.9	1.00	-0.66	66.4±22.0	62.1±23.8	1.00	-0.09
Laminin subunit beta-1	LAMB1	Lamb1	197	21.0±4.2	18.7±4.1	1.00	-0.10	21.4±2.6	16.3±1.2	0.91	-0.39	15.1±6.5	18.2±9.2	0.98	0.00	30.0±11.0	26.0±11.0	1.00	-0.01
Laminin subunit beta-2	LAMB2	Lamb2	197	53.1±4.3	54.7±2.9	1.00	0.01	38.1±1.4	48.3±4.3	0.22	0.10	42.5±16.6	55.3±17.3	0.80	0.39	44.8±13.5	37.8±15.2	1.00	-0.21
Laminin subunit gamma-1	LAMC1	Lamc1	177	61.9±6.2	62.6±4.8	1.00	0.02	62.2±0.8	58.4±0.3	0.98	-0.07	57.7±18.6	84.2±27.3	0.74	0.27	86.7±17.4	85.0±23.0	0.98	0.11
Galectin-1	LEG1	Lgals1	15	7.5±1.8	7.0±0.9	1.00	0.03	5.3±1.0	5.9±1.4	0.65	0.01	5.1±2.4	5.4±2.5	1.00	-0.04	4.0±2.6	4.3±2.6	0.99	0.15
**Galectin-3-binding protein**	**LG3BP**	**Lgals3bp**	**64**	**20.2±4.4**	**31.8±8.1**	**0.69**	**0.41**	**17.1±3.7**	**22.4±5.0**	**0.23**	**0.22**	**11.2±6.2**	**54.5±15.7**	**3.0E-06**	**1.73**	**7.7±6.6**	**49.5±16.3**	**0.02**	**2.35**
Lysyl oxidase homolog 2	LOXL2	Loxl2	87	10.2±2.0	5.3±2.6	0.70	-0.60	10.9±3.4	14.0±0.8	0.11	0.38	0.0±0.0	0.0±0.0	n.a.	n.a.	1.2±1.2	1.3±0.8	n.a.	n.a.
Latent-transforming growth factor beta-binding protein 2	LTBP2	Ltbp2	196	0.0±0.0	0.0±0.0	n.a.	n.a.	0.7±0.7	6.5±1.9	n.a.	n.a.	0.0±0.0	0.0±0.0	n.a.	n.a.	18.7±5.7	15.6±6.0	1.00	-0.12
Latent-transforming growth factor beta-binding protein 4	LTBP4	Ltbp4	179	0.0±0.0	0.0±0.0	n.a.	n.a.	0.0±0.0	0.0±0.0	n.a.	n.a.	3.6±2.1	0.9±0.9	n.a.	n.a.	13.9±5.6	6.3±2.6	1.00	-0.53
Lumican	LUM	Lum	38	10.2±1.2	17.0±1.3	0.13	0.44	4.8±2.4	5.0±1.9	0.57	0.06	28.3±7.1	19.5±7.5	1.00	-0.49	16.2±3.4	8.7±4.1	1.00	-0.57
Protein-lysine 6-oxidase	LYOX	Lox	47	14.8±2.6	10.0±3.5	0.68	-0.48	15.8±3.9	13.6±3.3	1.00	-0.23	0.0±0.0	0.0±0.0	n.a.	n.a.	0.7±0.8	0.0±0.0	n.a.	n.a.
Matrilin-2	MATN2	Matn2	107	3.0±0.6	3.9±0.8	0.80	0.32	0.0±0.0	0.0±0.0	n.a.	n.a.	6.5±3.0	2.9±2.2	1.00	-0.75	0.0±0.0	2.0±1.2	n.a.	n.a.
72 kDa type IV collagenase	MMP2	Mmp2	74	15.2±3.1	12.5±4.3	0.98	-0.26	18.6±1.0	18.0±2.3	1.00	-0.13	5.1±2.7	4.6±3.5	1.00	-0.31	3.1±1.8	1.9±1.2	1.00	-0.31
Stromelysin-1	MMP3	Mmp3	54	19.5±6.1	37.0±8.0	0.35	0.67	21.3±6.4	34.6±11.9	0.16	0.43	10.9±3.9	12.2±3.1	0.96	0.06	1.2±1.3	4.2±1.9	n.a.	n.a.
Nidogen-1	NID1	Nid1	137	72.9±5.4	73.7±7.0	1.00	-0.03	72.3±4.1	64.7±3.9	1.00	-0.12	76.2±14.7	94.4±20.3	0.69	0.16	81.7±19.7	85.1±22.5	0.98	0.10
Nidogen-2	NID2	Nid2	154	12.6±1.6	9.3±1.5	0.55	-0.34	0.7±0.7	2.9±1.2	n.a.	n.a.	0.0±0.0	0.0±0.0	n.a.	n.a.	0.0±0.0	0.0±0.0	n.a.	n.a.
Plasminogen activator inhibitor 1	PAI1	Serpine1	45	108.6±3.8	89.8±5.8	0.96	-0.09	203.8±16.3	210.7±32.6	1.00	-0.15	47.3±4.1	57.7±4.8	0.75	0.14	201.9±17.1	193.5±20.6	1.00	0.00
Procollagen C-endopeptidase enhancer 1	PCOC1	Pcolce	50	55.1±8.1	85.0±10.1	0.37	0.47	55.1±4.9	60.3±6.3	0.38	0.06	47.3±11.9	50.6±15.3	1.00	-0.18	37.5±9.9	35.4±6.7	1.00	-0.03
Protein disulfide-isomerase A3	PDIA3	Pdia3	57	30.5±6.0	15.4±2.0	0.24	-0.64	25.0±3.2	14.6±1.2	0.28	-0.56	64.3±7.5	58.8±11.6	1.00	-0.19	53.4±7.3	49.3±7.8	0.99	0.11
Protein disulfide-isomerase A6	PDIA6	Pdia6	48	5.1±1.7	5.0±0.8	0.78	0.25	6.5±0.6	4.7±0.4	0.40	-0.48	8.4±3.0	5.9±2.5	0.99	-0.33	7.8±2.6	6.4±1.5	1.00	-0.16

Supplemental Table 3. (continued)

				miR-21 mimic - Unstimulated cardiac fibroblasts				miR-21 mimic - TGF-β1-stimulated cardiac fibroblasts				miR-21 inhibitor (LNA) - Unstimulated cardiac fibroblasts				miR-21 inhibitor (LNA) - TGF-β1-stimulated cardiac fibroblasts			
				Mean ± SEM				Mean ± SEM				Mean ± SEM				Mean ± SEM			
**Full protein name**	**Accession ID**	**Gene**	**MW (kDa)**	**Control**	**miR-21**	**FDR**	**log2 FC**	**Control**	**miR-21**	**FDR**	**log2 FC**	**Control**	**miR-21**	**FDR**	**log2 FC**	**Control**	**miR-21**	**FDR**	**log2 FC**
Pigment epithelium-derived factor	PEDF	Serpinf1	46	68.8±5.1	72.9±12.8	1.00	-0.04	93.1±3.8	87.3±8.1	0.94	-0.05	105.6±16.0	125.4±19.9	0.28	0.25	139.3±17.8	146.9±13.2	0.93	0.20
Basement membrane-specific heparan sulfate proteoglycan core protein	PGBM	Hspg2	398	102.9±8.6	71.3±9.9	0.63	-0.33	81.0±18.0	95.3±8.6	0.24	0.15	20.1±6.6	19.3±6.1	1.00	-0.12	66.4±16.4	52.0±19.7	1.00	-0.40
Biglycan	PGS1	Bgn	42	95.8±7.1	86.7±15.5	0.97	-0.15	147.6±5.1	140.4±12.6	0.45	0.04	59.8±12.7	75.7±14.0	0.58	0.19	104.6±24.8	107.8±18.3	0.79	0.25
Decorin	PGS2	Dcn	40	61.0±6.5	55.8±2.3	0.99	-0.09	42.1±3.0	38.0±2.7	1.00	-0.22	110.9±22.1	93.7±18.4	1.00	-0.19	67.7±12.8	63.9±11.4	0.99	0.05
Procollagen-lysine,2-oxoglutarate 5-dioxygenase 1	PLOD1	Plod1	84	4.3±0.9	3.3±1.2	1.00	-0.06	2.2±1.4	3.8±1.9	n.a.	n.a.	0.0±0.0	0.0±0.0	n.a.	n.a.	0.0±0.0	2.0±1.2	n.a.	n.a.
Procollagen-lysine,2-oxoglutarate 5-dioxygenase 2	PLOD2	Plod2	84	4.7±0.8	3.1±0.6	0.84	-0.21	4.9±0.8	2.7±1.7	1.00	-0.40	0.0±0.0	0.0±0.0	n.a.	n.a.	1.4±1.4	0.0±0.0	n.a.	n.a.
Procollagen-lysine,2-oxoglutarate 5-dioxygenase 3	PLOD3	Plod3	85	5.8±0.6	7.6±1.7	0.67	0.30	6.2±1.0	10.1±1.4	0.13	0.27	2.7±0.9	6.2±2.8	0.30	0.55	11.9±4.3	12.3±4.4	0.99	0.11
Periostin	POSTN	Postn	93	27.4±5.6	30.0±7.0	1.00	0.05	81.0±7.4	100.0±10.5	0.16	0.17	12.3±7.2	17.2±9.5	0.95	0.18	92.2±33.2	78.6±28.8	1.00	-0.30
Peptidyl-prolyl cis-trans isomerase A	PPIA	Ppia	18	16.2±5.3	9.4±2.7	0.86	-0.38	8.9±2.6	8.2±1.0	0.81	-0.05	6.4±3.7	11.2±0.9	0.21	0.45	7.1±1.6	8.2±1.2	0.96	0.22
Peptidyl-prolyl cis-trans isomerase B	PPIB	Ppib	24	13.6±2.4	8.3±1.3	0.40	-0.48	10.9±2.0	7.6±1.4	0.79	-0.53	17.3±3.6	16.2±1.7	1.00	-0.11	10.7±3.3	17.0±2.0	0.71	0.52
Peroxiredoxin-1	PRDX1	Prdx1	22	17.7±3.9	17.1±3.2	1.00	0.05	15.7±4.3	11.2±2.5	1.00	-0.38	18.0±3.5	20.2±3.4	0.96	0.03	15.0±3.1	26.3±6.2	0.81	0.54
Peroxiredoxin-2	PRDX2	Prdx2	22	5.4±0.8	3.8±0.8	0.76	-0.29	3.6±1.0	3.4±0.6	0.86	-0.07	5.5±1.9	7.4±1.6	0.86	0.11	7.6±1.0	7.8±1.2	1.00	0.00
Peroxiredoxin-6	PRDX6	Prdx6	25	3.9±2.5	4.4±2.4	1.00	0.14	6.9±1.7	3.8±1.4	1.00	-0.58	6.2±1.1	8.2±2.9	0.99	-0.03	2.1±1.3	7.3±4.2	0.95	0.85
Prolargin	PRELP	Prelp	43	8.9±3.9	0.0±0.0	0.59	-8.26	3.4±1.3	2.0±1.2	1.00	-0.29	5.9±3.5	11.1±4.0	0.63	0.24	0.8±0.9	0.7±0.8	n.a.	n.a.
Pentraxin-related protein PTX3	PTX3	Ptx3	42	36.2±5.1	49.6±4.0	0.91	0.18	26.8±1.7	33.3±3.1	0.26	0.11	24.5±2.9	41.4±7.8	0.43	0.36	22.6±2.4	30.9±4.8	0.88	0.28
Extracellular superoxide dismutase [Cu-Zn]	SODE	Sod3	27	28.4±4.0	27.7±4.1	0.95	-0.17	27.3±2.6	24.9±3.0	1.00	-0.19	15.8±5.5	18.1±7.6	1.00	-0.11	22.8±6.1	23.2±5.9	0.99	0.05
Serine protease inhibitor A3N	SPA3N	Serpina3n	47	36.4±8.9	42.5±5.6	1.00	0.07	27.9±4.3	26.8±5.6	1.00	-0.14	40.6±11.3	57.3±16.7	0.91	0.13	39.7±11.9	25.5±7.7	1.00	-0.41
SPARC	SPRC	Sparc	34	92.7±5.4	98.8±12.7	0.94	0.12	117.7±11.2	106.6±7.4	1.00	-0.12	41.6±4.2	44.8±4.4	1.00	-0.10	58.6±3.3	54.5±2.7	1.00	0.00
Tenascin	TENA	Tnc	232	26.0±4.4	20.0±5.5	0.76	-0.44	35.8±4.9	35.1±5.4	1.00	-0.14	12.5±5.9	23.2±9.7	0.24	0.61	23.5±10.0	30.4±14.2	0.98	0.29
Tetranectin	TETN	Clec3b	22	3.0±1.0	2.5±0.9	0.96	-0.16	0.0±0.0	0.0±0.0	n.a.	n.a.	7.1±3.2	4.8±1.8	1.00	-0.22	0.0±0.0	0.0±0.0	n.a.	n.a.
Metalloproteinase inhibitor 1	TIMP1	Timp1	23	10.4±0.7	8.7±3.3	1.00	-0.09	20.1±4.9	26.4±3.9	0.24	0.21	9.6±4.6	6.9±5.6	1.00	-0.76	10.2±5.4	15.0±5.4	0.79	0.78
Metalloproteinase inhibitor 2	TIMP2	Timp2	24	9.5±3.2	12.6±2.0	0.96	0.21	12.3±1.7	13.5±1.2	0.36	0.10	11.3±4.6	6.9±2.6	0.99	-0.39	1.6±0.9	0.0±0.0	n.a.	n.a.
Thrombospondin-1	TSP1	Thbs1	130	7.1±1.2	4.4±0.7	0.79	-0.34	19.6±3.2	25.0±5.6	0.56	0.04	4.2±3.2	3.3±2.2	1.00	-0.22	9.8±2.4	10.1±2.1	0.99	0.07
Thrombospondin-2	TSP2	Thbs2	130	3.6±1.5	9.4±1.7	0.26	0.94	0.9±0.9	7.7±1.2	n.a.	n.a.	12.6±6.1	1.4±0.9	0.67	-1.47	3.1±1.1	3.3±2.1	0.99	0.12
**Vascular cell adhesion protein 1**	**VCAM1**	**Vcam1**	**81**	**8.8±2.0**	**7.6±1.1**	**1.00**	**-0.01**	**7.8±1.8**	**6.8±1.7**	**1.00**	**-0.28**	**1.6±1.0**	**16.3±1.3**	**<1.0E-06**	**1.50**	**2.2±0.9**	**10.8±1.9**	**3.5E-05**	**1.25**
Vinculin	VINC	Vcl	117	8.3±4.7	1.9±1.9	n.a.	n.a.	8.0±2.3	0.7±0.7	0.11	-1.41	13.0±3.3	14.3±4.3	1.00	-0.09	15.5±4.8	17.3±4.6	0.99	0.22

Supplemental Table 4. MiR-21 null heart ECM protein analysis. Analysis was performed using the 3-step extraction method, involving sequential incubation in NaCl, SDS and GuHCl to enrich for ECM proteins. Combined analysis of NaCl and GuHCl fractions by LC-MS/MS was annotated using the Matrisome database for murine ECM and ECM-associated proteins. Values indicate normalized total precursor intensity. FDR-adjusted p-values (q-values) were calculated using the 2-stage step-up method of Benjamini, Krieger and Yekutieli. Q for significant discovery was set to 5%.

Protein ID	Gene	miR-21$^{+/+}$	miR-21$^{-/-}$	p-value	q-value
CO1A1_MOUSE	Col1a1	2.5E+10	2.2E+10	0.6351	>0.9999
CO3A1_MOUSE	Col3a1	2.5E+10	2.2E+10	0.7349	>0.9999
CO1A2_MOUSE	Col1a2	1.4E+10	1.2E+10	0.6783	>0.9999
PGBM_MOUSE	Hspg2	1.4E+10	1.2E+10	0.3143	>0.9999
ANXA6_MOUSE	Anxa6	9.5E+09	9.2E+09	0.7367	>0.9999
NID1_MOUSE	Nid1	7.5E+09	6.7E+09	0.5165	>0.9999
A1AT2_MOUSE	Serpina1b	6.1E+09	6.8E+09	0.5223	>0.9999
LEG1_MOUSE	Lgals1	4.8E+09	5.0E+09	0.7817	>0.9999
ANXA5_MOUSE	Anxa5	3.9E+09	4.4E+09	0.1604	>0.9999
LAMA2_MOUSE	Lama2	3.5E+09	3.2E+09	0.6058	>0.9999
LAMC1_MOUSE	Lamc1	3.5E+09	3.1E+09	0.5586	>0.9999
PGS2_MOUSE	Dcn	3.1E+09	2.9E+09	0.7459	>0.9999
ANXA2_MOUSE	Anxa2	3.0E+09	3.3E+09	0.6398	>0.9999
SPA3K_MOUSE	Serpina3k	2.7E+09	2.8E+09	0.8040	>0.9999
TGM2_MOUSE	Tgm2	2.6E+09	2.7E+09	0.7984	>0.9999
CO6A1_MOUSE	Col6a1	2.2E+09	2.5E+09	0.5046	>0.9999
LAMB2_MOUSE	Lamb2	1.7E+09	1.7E+09	0.9022	>0.9999
LAMB1_MOUSE	Lamb1	1.6E+09	1.4E+09	0.3810	>0.9999
PZP_MOUSE	Pzp	1.6E+09	1.6E+09	0.9815	>0.9999
CO6A2_MOUSE	Col6a2	1.5E+09	1.6E+09	0.8764	>0.9999
MFAP5_MOUSE	Mfap5	1.3E+09	8.0E+08	0.2806	>0.9999
CO5A2_MOUSE	Col5a2	1.2E+09	1.2E+09	0.8809	>0.9999
SERPH_MOUSE	Serpinh1	1.2E+09	1.5E+09	0.3230	>0.9999
S10A1_MOUSE	S100a1	1.1E+09	1.1E+09	0.7563	>0.9999
COFA1_MOUSE	Col15a1	1.1E+09	1.1E+09	0.9553	>0.9999
HEMO_MOUSE	Hpx	1.0E+09	1.0E+09	0.9673	>0.9999
CATD_MOUSE	Ctsd	1.0E+09	9.7E+08	0.8106	>0.9999
NID2_MOUSE	Nid2	9.7E+08	7.9E+08	0.2888	>0.9999
CO4A1_MOUSE	Col4a1	9.2E+08	7.8E+08	0.7734	>0.9999
CO5A1_MOUSE	Col5a1	8.2E+08	8.4E+08	0.9057	>0.9999
CO4A2_MOUSE	Col4a2	7.8E+08	5.9E+08	0.5286	>0.9999
DERM_MOUSE	Dpt	7.2E+08	6.3E+08	0.4697	>0.9999
S10AA_MOUSE	S100a10	7.1E+08	8.3E+08	0.2888	>0.9999
ANT3_MOUSE	Serpinc1	5.5E+08	5.2E+08	0.8099	>0.9999
FBN1_MOUSE	Fbn1	5.5E+08	5.0E+08	0.8727	>0.9999
LUM_MOUSE	Lum	5.2E+08	6.0E+08	0.4485	>0.9999
KNG1_MOUSE	Kng1	5.1E+08	4.7E+08	0.7729	>0.9999
FINC_MOUSE	Fn1	4.4E+08	6.8E+08	0.3155	>0.9999
FIBB_MOUSE	Fgb	3.9E+08	5.1E+08	0.4691	>0.9999
LAMA4_MOUSE	Lama4	3.8E+08	4.2E+08	0.6975	>0.9999
ANXA7_MOUSE	Anxa7	3.5E+08	2.1E+08	0.0790	>0.9999
PGS1_MOUSE	Bgn	3.4E+08	3.0E+08	0.8318	>0.9999
ADIPO_MOUSE	Adipoq	3.0E+08	3.3E+08	0.3310	>0.9999
ANX11_MOUSE	Anxa11	2.7E+08	3.1E+08	0.4079	>0.9999
FIBA_MOUSE	Fga	2.7E+08	3.0E+08	0.8466	>0.9999
CATB_MOUSE	Ctsb	2.5E+08	1.9E+08	0.3092	>0.9999
PRELP_MOUSE	Prelp	2.3E+08	2.0E+08	0.5583	>0.9999
PPN_MOUSE	Papln	2.0E+08	2.1E+08	0.9080	>0.9999
FIBG_MOUSE	Fgg	1.7E+08	2.9E+08	0.4163	>0.9999
LAMA5_MOUSE	Lama5	1.4E+08	1.5E+08	0.8689	>0.9999
ANXA4_MOUSE	Anxa4	1.4E+08	1.3E+08	0.8081	>0.9999
TINAL_MOUSE	Tinagl1	1.3E+08	8.4E+07	0.2736	>0.9999
CO6A6_MOUSE	Col6a6	1.1E+08	4.5E+07	0.0244	>0.9999
AGRIN_MOUSE	Agrn	8.4E+07	6.7E+07	0.5389	>0.9999
VMA5A_MOUSE	Vwa5a	7.4E+07	9.6E+07	0.3572	>0.9999

Supplemental Table 5. Proteins identified in the thrombin-induced releasate of washed platelets, isolated from mice treated with antagomiR-21 or -control. The platelet releasate was analyzed by gel LC-MS/MS using an LTQ-Orbitrap XL. Values shown are mean±SEM of normalized spectral counts (NSpC), based on three biological replicates (each replicate consisting of pooled platelets from 4 mice) for each condition. Identified releasate proteins with an average NSpC >5 for at least one sample category are shown. FC reflect the ratio between the average NSpC in samples after antagomiR-21 treatment and after control-antagomiR with a t-test evaluating statistical significance.

Accession ID	Gene ID	MW (kDa)	Control (NSpC)	AntagomiR -21 (NSpC)	P-value	log 2 FC	α-granule lumen (GO:0031093)	δ-granule lumen (GO:0031089)
1433Z_MOUSE	Ywhaz	28	85.6±12.1	113.5±6.1	0.1338	0.41		
6PGD_MOUSE	Pgd	53	16.6±8.6	22.5±2.4	0.5743	0.43		
A1AT1_MOUSE	Serpina1a	46	61.8±19.1	64.4±5.9	0.9080	0.06	x	
A2M_MOUSE	Pzp	166	40.3±34.8	46.8±5.9	0.8698	0.22	x	
ACTN1_MOUSE	Actn1	103	99.9±11.7	87.2±26.9	0.6963	-0.20	x	
ALDOA_MOUSE	Aldoa	39	79.2±10.1	86.1±7.4	0.6128	0.12	x	
ANT3_MOUSE	Serpinc1	52	5.6±2.3	1.3±1.3	0.1871	-2.16		
ANXA5_MOUSE	Anxa5	36	30.0±7.7	26.7±2.2	0.7202	-0.17		
CAP1_MOUSE	Cap1	52	94.4±4.3	97.2±3.1	0.6228	0.04		
CATA_MOUSE	Cat	60	4.5±1.4	7.2±2.3	0.3679	0.69		
CLIC1_MOUSE	Clic1	27	15.4±3.2	16.3±1.6	0.8259	0.08		
COF1_MOUSE	Cfl1	19	21.2±5.1	16.8±3.9	0.5301	-0.34		
COR1A_MOUSE	Coro1a	51	84.7±7.0	87.4±9.2	0.8294	0.04		
COR1C_MOUSE	Coro1c	53	7.3±4.4	4.7±2.4	0.6304	-0.65		
ECM1_MOUSE	Ecm1	63	8.4±4.3	0.8±0.8	0.2163	-3.48		x
EMIL1_MOUSE	Emilin1	108	6.1±3.1	5.4±4.0	0.9056	-0.16		
ENOA_MOUSE	Eno1	47	61.8±11.0	69.2±8.5	0.6225	0.16		
ENPL_MOUSE	Hsp90b1	92	24.2±4.5	17.7±8.7	0.5541	-0.45		
FA10_MOUSE	F10	54	10.4±2.1	6.3±2.7	0.2996	-0.72		
FHL1_MOUSE	Fhl1	32	13.1±3.8	5.7±4.7	0.2914	-1.19		
FIBA_MOUSE	Fga	87	154.7±44.1	55.4±14.3	0.1430	-1.48	x	
FIBB_MOUSE	Fgb	55	111.5±35.5	44.2±9.8	0.1924	-1.34	x	
FIBG_MOUSE	Fgg	49	110.0±36.5	25.8±8.9	0.1412	-2.09	x	
**FINC_MOUSE**	**Fn1**	**273**	**9.4±1.7**	**2.1±1.0**	**0.0300**	**-2.18**	**x**	
G3P_MOUSE	Gapdh	36	24.7±4.6	39.3±15.0	0.4351	0.67		
GDIB_MOUSE	Gdi2	51	37.3±10.6	46.2±8.3	0.5483	0.31		
GDN_MOUSE	Serpine2	44	28.8±10.0	15.7±2.9	0.3205	-0.88	x	

Accession ID	Gene ID	MW (kDa)	Control (NSpC)	AntagomiR -21 (NSpC)	P-value	log 2 FC	α-granule lumen (GO:0031093)	δ-granule lumen (GO:0031089)
GELS_MOUSE	Gsn	86	38.5±6.4	21.5±11.4	0.2804	-0.84		
GP1BA_MOUSE	Gp1ba	80	67.8±7.7	61.2±1.4	0.4832	-0.15		
GPV_MOUSE	Gp5	63	14.7±5.4	9.4±1.9	0.4346	-0.65		
GRP78_MOUSE	Hspa5	72	54.6±20.0	78.9±17.0	0.4103	0.53		
HPSE_MOUSE	Hpse	60	8.6±3.3	4.6±2.4	0.3904	-0.91		
HS105_MOUSE	Hsph1	96	7.1±3.6	3.5±3.5	0.5204	-1.01		
HS74L_MOUSE	Hspa4l	94	5.5±2.8	4.8±4.8	0.9020	-0.21		
HS90A_MOUSE	Hsp90aa1	85	28.3±6.4	19.1±4.2	0.3041	-0.57		
HS90B_MOUSE	Hsp90ab1	83	19.9±10.1	3.3±3.3	0.2370	-2.61		
HSP74_MOUSE	Hspa4	94	20.7±2.5	20.7±5.5	0.9983	0.00		
HSP7C_MOUSE	Hspa8	71	90.8±9.2	120.2±7.3	0.0693	0.40		
ILEUA_MOUSE	Serpinb1a	43	35.5±8.1	30.8±3.2	0.6366	-0.20		
ILK_MOUSE	Ilk	51	18.1±12.6	4.6±4.1	0.4016	-1.97		
ITA2B_MOUSE	Itga2b	113	6.7±2.4	0.8±0.8	0.1274	-3.15	x	
KNG1_MOUSE	Kng1	73	11.5±3.1	11.8±4.2	0.9622	0.03	x	
KPYM_MOUSE	Pkm	58	53.7±10.7	32.4±0.9	0.1830	-0.73		
LEGL_MOUSE	Lgalsl	19	8.3±1.0	6.8±0.9	0.3317	-0.29		
LTBP1_MOUSE	Ltbp1	187	5.6±3.2	0.0±0.0	n.a.	n.a.		
LYZ2_MOUSE	Lyz2	17	31.2±5.9	26.1±6.9	0.6056	-0.26		
PARVB_MOUSE	Parvb	42	15.7±6.2	5.7±1.0	0.2438	-1.46		
PDIA3_MOUSE	Pdia3	57	59.2±2.9	81.4±23.2	0.4391	0.46		
PDIA4_MOUSE	Pdia4	72	8.0±4.3	7.3±3.9	0.9098	-0.13		
PDLI1_MOUSE	Pdlim1	36	7.4±0.8	5.5±0.1	0.1360	-0.45		
PLEK_MOUSE	Plek	40	46.9±8.7	52.4±0.6	0.5873	0.16		
PLF4_MOUSE	Pf4	11	28.4±12.0	8.8±8.8	0.2638	-1.69	x	
PLMN_MOUSE	Plg	91	26.5±6.2	5.5±3.8	0.0552	-2.26	x	
PLSI_MOUSE	Pls1	70	8.5±5.8	0.0±0.0	n.a.	n.a.		
PPIA_MOUSE	Ppia	18	43.1±5.7	42.5±4.9	0.9404	-0.02		
PPIB_MOUSE	Ppib	24	20.4±4.4	15.3±6.3	0.5422	-0.42		
PRDX1_MOUSE	Prdx1	22	13.3±1.8	13.2±4.0	0.9851	-0.01		
PRDX6_MOUSE	Prdx6	25	17.3±0.9	17.5±3.2	0.9574	0.02		
PROF1_MOUSE	Pfn1	15	69.2±7.4	88.3±14.1	0.3183	0.35		
PROS_MOUSE	Pros1	75	6.9±5.5	0.0±0.0	n.a.	n.a.	x	
PSA_MOUSE	Npepps	103	8.8±4.1	6.0±4.0	0.6444	-0.57		
PSA6_MOUSE	Psma6	27	7.7±1.0	6.7±1.2	0.5554	-0.20		

Accession ID	Gene ID	MW (kDa)	Control (NSpC)	AntagomiR -21 (NSpC)	P-value	log 2 FC	α-granule lumen (GO:0031093)	δ-granule lumen (GO:0031089)
PUR9_MOUSE	Atic	64	16.7±2.7	14.4±7.2	0.7836	-0.22		
QSOX1_MOUSE	Qsox1	83	16.8±7.7	6.4±4.8	0.3272	-1.40	x	
SODC_MOUSE	Sod1	16	13.2±2.7	18.3±2.5	0.2341	0.47		
SPA3K_MOUSE	Serpina3k	47	24.0±13.4	24.3±5.4	0.9816	0.02	x	
SPA3N_MOUSE	Serpina3n	47	5.5±2.8	8.9±4.4	0.5556	0.70	x	
SRC_MOUSE	Src	61	7.4±0.8	13.0±4.4	0.3290	0.82		
SRGN_MOUSE	Srgn	17	16.8±5.0	3.3±3.3	0.0982	-2.36	x	
TAGL2_MOUSE	Tagln2	22	32.9±3.6	34.3±3.6	0.7964	0.06		
TERA_MOUSE	Vcp	89	47.1±5.3	41.7±9.6	0.6545	-0.18		
**TGFB1_MOUSE**	**Tgfb1**	**44**	**9.2±2.2**	**1.3±1.3**	**0.0472**	**-2.86**	**x**	
TPIS_MOUSE	Tpi1	32	22.0±3.0	22.5±1.1	0.9013	0.03		
TSP1_MOUSE	Thbs1	130	421.9±54.4	289.4±31.2	0.1198	-0.54	x	
TYB4_MOUSE	Tmsb4x	6	6.5±0.6	3.4±1.7	0.2042	-0.94	x	
URP2_MOUSE	Fermt3	76	19.1±10.6	0.0±0.0	n.a.	n.a.	x	
**VWF_MOUSE**	**Vwf**	**309**	**22.4±4.9**	**3.5±3.5**	**0.0400**	**-2.67**	**x**	
WDR1_MOUSE	Wdr1	66	66.8±5.7	66.7±3.5	0.9909	0.00		
ZYX_MOUSE	Zyx	61	7.9±2.3	3.7±0.4	0.2085	-1.08		
